# Repurposing strategies on pyridazinone-based series by pharmacophore- and structure-driven screening

**DOI:** 10.1080/14756366.2020.1760261

**Published:** 2020-05-05

**Authors:** Giuseppe Floresta, Letizia Crocetti, Maria Paola Giovannoni, Pierfrancesco Biagini, Agostino Cilibrizzi

**Affiliations:** a Institute of Pharmaceutical Science, King’s College London, London, UK; b NEUROFARBA, Pharmaceutical and Nutraceutical Section, University of Florence, Sesto Fiorentino (Fi), Italy

**Keywords:** Pyridazinones, pharmacophore model, structure-based screening, drug repurposing, aspartate aminotransferase

## Abstract

We report here *in silico* repurposing studies on 52 new pyridazinone-based small-molecules through inverse virtual screening (iVS) methodologies. These analogues were originally designed as formyl peptide receptor (FPR) ligands. As it is sometimes the case in drug discovery programmes, subsequent biological screening demonstrated the inefficacy of the molecules in binding FPRs, failing in the identification of new hits. Through a focussed drug-repurposing approach we have defined a variety of potential targets that are suitable to interact with this library of pyridazinone-based analogues. A two-step approach has been conducted for computational analysis. Specifically, the molecules were initially processed through a pharmacophore-based screening. Secondly, the resulting features of binding were investigated by docking studies and following molecular dynamic simulations, in order to univocally confirm “pyridazinone-based ligand-target protein” interactions. Our findings propose aspartate aminotransferase as the most favourable repurposed target for this small-molecule series, worth of additional medicinal chemistry investigations in the field.

## Graphical Abstract

**Figure:**
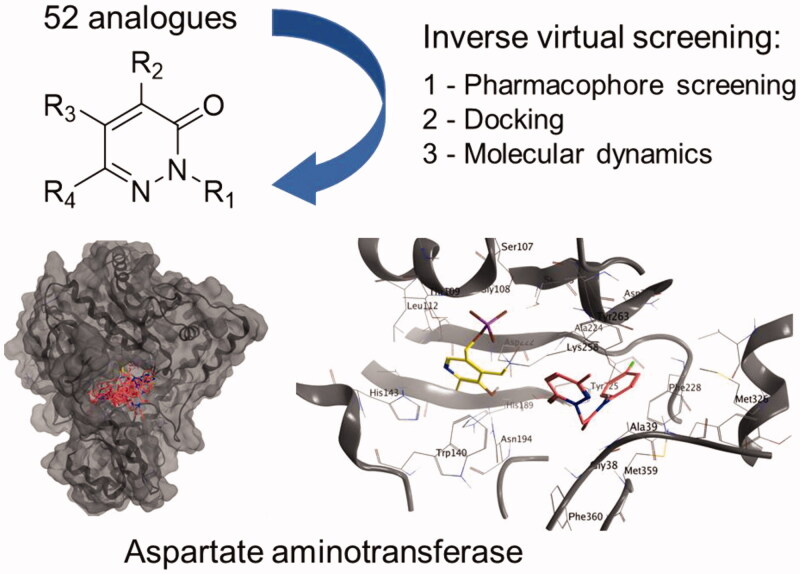

## 1. Introduction

Heterocyclic-based small molecules possess the ability to mimic the structure of endogenous ligands and reversibly bind various biologically relevant targets, determining a wide range of possible biological activities^1–3^. In medicinal chemistry, one important benefit associated to heterocycles is the possibility to create such a desired library of analogues based on a specific *core*, allowing rapid screening protocols and structure-activity relationships (SARs) for a particular target of interest^4^. However, very frequently the molecular design does not correlate with positive outcomes in biological tests. With the increase of new chemical entities discovered, there is a proportional boost of the number of molecules that fail during the twisted path of drug development. Clearly, this raises concerns with regard to the optimisation of the use of economic resources and time. As an example, by taking into accounts common failures in the process, it has been estimated that the average cost of developing a new drug ranges from two to three billion dollars and not less than 13–15 years are required to introduce the medicine into the market^5^. In line with this, only 10% of the drugs that enter into clinical trials are approved by regulatory agencies, while the remaining 90% fails due to toxicity issues or inefficacy linked to limited predictive value of preclinical studies. Specifically, more the 60% of molecules fail in phase II and 45% fail in phase III^6^. There is consensus in the medicinal chemistry community that the high percentage of failures in drug development can be a direct consequence of the R&D efficacy in identifying the appropriate drug response on the target of interest, due to limited availability of suitable preclinical disease models^7^.

Lately explored strategies of “drug repurposing” (or “drug repositioning”) have demonstrated a useful tool at the different stages of drug development, by assessing and validating new possible therapeutic effects for existing drug candidates, as well as by identifying alternative targets for abandoned compound series^8^^,^^9^. Interestingly, repositioned drugs currently represent almost the 30% of all the new medicines that reach their first market^10^^,^^11^.

In this context, *in silico* methods, such as machine learning, data-mining, and network-based approaches, offer an unprecedented and cost friendly opportunity to predict possible drug repositioning candidates, by employing large and heterogeneous data sources during the entire process of drug development^12^^,^^13^. Thus, computer-aided molecular screening is a crucial tool in drug design/discovery and computational techniques represent an important resource for prompt evaluation of possible links between compounds and biological/pharmacological effects^14^. Various approaches, such as structure-based, ligand-based, virtual screening and inverse virtual screening (iVS), are constantly adopted in various drug discovery settings, spanning from the hit identification to the lead optimisation stages^15–17^. Pyridazinone-based small-molecules are a validated scaffold for the development of enzyme and G-protein coupled receptor (GPCR) binders^18^^,^^19^. All of the 52 new molecules reported here were designed as agonists of formyl peptide receptors (FPRs)^20–22^, a small family of GPCRs. As the biological evaluation on this series demonstrated the lack of efficacy for all the terms to establish interactions with FPRs, we have focussed our attention on the possible repurposing of these same molecules towards alternative biological targets. To this end, we report here the *in silico* investigation carried out on the pyridazinone-based library through a two-step iVS analysis. The compounds were firstly screened in a pharmacophore-based iVS against 23236 proteins covering 16159 pharmacophore models formerly predicted as drug able binding sites. Subsequently, the binding capabilities of the 52 terms have been processed through docking studies. Further exploratory analyses on the binding poses of selected hits were performed with molecular dynamic simulations, to validate the outcomes of the docking analysis and confirm effective ligand/protein interactions. Our findings define the aspartate aminotransferase as a valid biological target for efficient repurposing opportunities of this heterocyclic small-molecule series.

## 2. Results and discussion

### 2.1. Heterocyclic small-molecule dataset

The dataset of compounds is composed by 52 terms (Tables 1–3) which have been obtained through standard synthetic methodologies (see Section 1, Supporting Information). The experimental procedures and characterisation data of all new intermediates and final compounds are reported in Supporting Information (Section 2)^18^^,^^23–34^.

**Table 1.** Structures of analogues **1**–**9**.


Compd	*R*_1_	*R*_2_	*R*_3_	*R*_4_
**1a**	C_6_H_4_-OCH_3_ (p)	C_6_H_4_-O(CH_2_)_3_CH_3_ (p)	OCH_3_	H
**1b**	C_6_H_4_-OCH_3_ (m)	C_6_H_4_-O(CH_2_)_3_CH_3_ (p)	OCH_3_	H
**2a**	C_6_H_4_-OCH_3_ (p)	Cl	C_6_H_4_-O(CH_2_)_3_CH_3_ (p)	H
**2b**	C_6_H_4_-OCH_3_ (m)	Cl	C_6_H_4_-O(CH_2_)_3_CH_3_ (p)	H
**3a**	CO-NH-C_6_H_4_-F (p)	H	H	CH_3_
**3b**	CO-NH-C_6_H_4_-3,4-methlyenedioxy	H	H	CH_3_
**4a**	C_6_H_5_	NO_2_	COCH_3_	C_6_H_5_
**4b**	C_6_H_4_-CN (m)	NO_2_	COCH_3_	C_6_H_5_
**5a**	C_6_H_5_	Cl	COCH_3_	C_6_H_5_
**5b**	C_6_H_4_-CN (m)	Cl	COCH_3_	C_6_H_5_
**6a**	C_6_H_5_	Br	COCH_3_	C_6_H_5_
**6b**	C_6_H_4_-CN (m)	Br	COCH_3_	C_6_H_5_
**7**	C_6_H_5_	COCH_3_	NH_2_	C_6_H_5_
**8**	CO-NH-C_6_H_4_-Br (p)	CH_2_-C_6_H_4_-(m)CO-NH-C_6_H_4_-Br (p)	H	CH_3_
**9**	CO-NH-C_6_H_4_-Br (p)	N-(C_6_H_4_-OCH_3_)_2_ (p)	H	CH_3_

**Table 2.** Structures of analogues **10**–**12**.


Compd	R	R_1_
**10a**	Ph	
**11**	Ph	
**10b**	Ph	
**10c**	Ph	
**12**	Ph	
**10d**	Ph	
**10e**	Ph	
**10f**	CH_3_	
**10g**	CH_3_	

**Table 3.** Structures of analogues **13**–**21**.


Compd	R	R_1_	R_2_	R_3_
**13a**	Ph	Ph	NH_2_	CONHC_3_H_7_
**13c**	Ph	Ph	NH_2_	COOcC_6_H_11_
**13b**	Ph	Ph	NH_2_	
**17c**	4-F-Ph	Ph	NHCOiC_3_H_7_	H
**18**	Ph	CH_2_Ph	NH_2_	CONHtC_4_H_9_
**19**	Ph	H	NH_2_	COOCH_2_Ph
**20**	Ph	CH_2_Ph	NH_2_	COOCH_2_Ph
**14a**	Ph	CH_3_	NH_2_	COCH = CHPh
**15a**	Ph	CH_3_	NH_2_	COCH_2_CH_2_Ph
**14b**	Ph	C_3_H_7_	NH_2_	COCH = CHPh
**15b**	Ph	C_3_H_7_	NH_2_	COCH_2_CH_2_Ph
**14c**	4-F-Ph	CH_3_	NH_2_	COCH = CHPh
**15g**	Ph	C_2_H_5_	NH_2_	
**15i**	Ph	C_3_H_7_	NH_2_	
**15h**	Ph	C_3_H_7_	NH_2_	
**15l**	Ph	Ph	NH_2_	
**15f**	Ph	Ph	NH_2_	COCH_2_CH_2_Ph
**17b**	Ph	Ph	NHCOiC_3_H_7_	H
**17a**	Ph	Ph	NHCOC_4_H_9_	H
**15e**	cC_6_H_11_	C_3_H_7_	NH_2_	COCH_2_CH_2_Ph
**15c**	Ph	C_4_H_9_	NH_2_	COCH_2_CH_2_Ph
**15d**	Ph	iC_3_H_7_	NH_2_	COCH_2_CH_2_Ph
**16b**	4-F-Ph	Ph	NH_2_	H
**21a**	Ph	CH_2_CONHnC_3_H_7_	H	Ph
**21b**	Ph	CH_2_CONHiC_3_H_7_	H	Ph
**21c**	Ph	CH_2_CONHcC_5_H_9_	H	Ph
**21d**	Ph	CH_2_CONHnC_3_H_7_	Ph	H
**21e**	Ph	CH_2_CONHiC_3_H_7_	Ph	H

### 2.2. Computational repurposing

As representative compounds, 17 compounds (**1–3a**, **5a**, **7–9**, **10b**,**g**, **11**, **13a**, **15a**,**f**, **17a**, **20**, **21a**, **21d**) were selected for following molecular modelling studies. The compound library was firstly screened in a pharmacophore mapping strategy based on a PharmMapper (http://lilab-ecust.cn/pharmmapper/) to identify potential molecular targets for the synthesised compounds^35^^,^^36^. PharmMapper is backed up by a large, *in-house* repertoire of pharmacophore database extracted from all the targets in different database such as TargetBank, DrugBank, BindingDB and PDTD. 16159 receptor-based pharmacophore models were used during our PharmMapper screening. The results of the pharmacophore map iVS are reported in Tables S1–S17 (Supporting Information). With the aim of finding a common target for our dataset of compounds, only the best 50 targets identified for each molecule are included here and only the best 10 results for each molecule were further analysed (see results in Table S18, Supporting Information). Interestingly, various targets were highlighted as common targets for different molecules. In particular, cytoplasmic aspartate aminotransferase, protein MG296 homologue, sensor protein fixL, ATP-dependent protease hslV, dehydrogenase and superoxide dismutase, demonstrated common targets for 4–6 molecules between the 10 best fitting targets of the pharmacophore-based iVS (Table S18, Supporting Information). The cytoplasmic aspartate aminotransferase (AST) resulted a shared target by 6 different compounds (i.e. 35% of the molecular dataset). This protein is a liver pyridoxal phosphate dependent enzyme involved in gluconeogenesis and amino acid metabolism. Increased levels of activity of AST has been observed in a number of conditions, such as liver metabolic syndrome, atherosclerosis and diabetes (type I and II), and the use of small molecule that can inhibit the protein activity is under evaluation for the treatment of diabetes^37^. Furthermore, AST has been also proposed as a promising biological target for the development of anti-neoplastic agents^38^^,^^39^.

**Figure 1.**
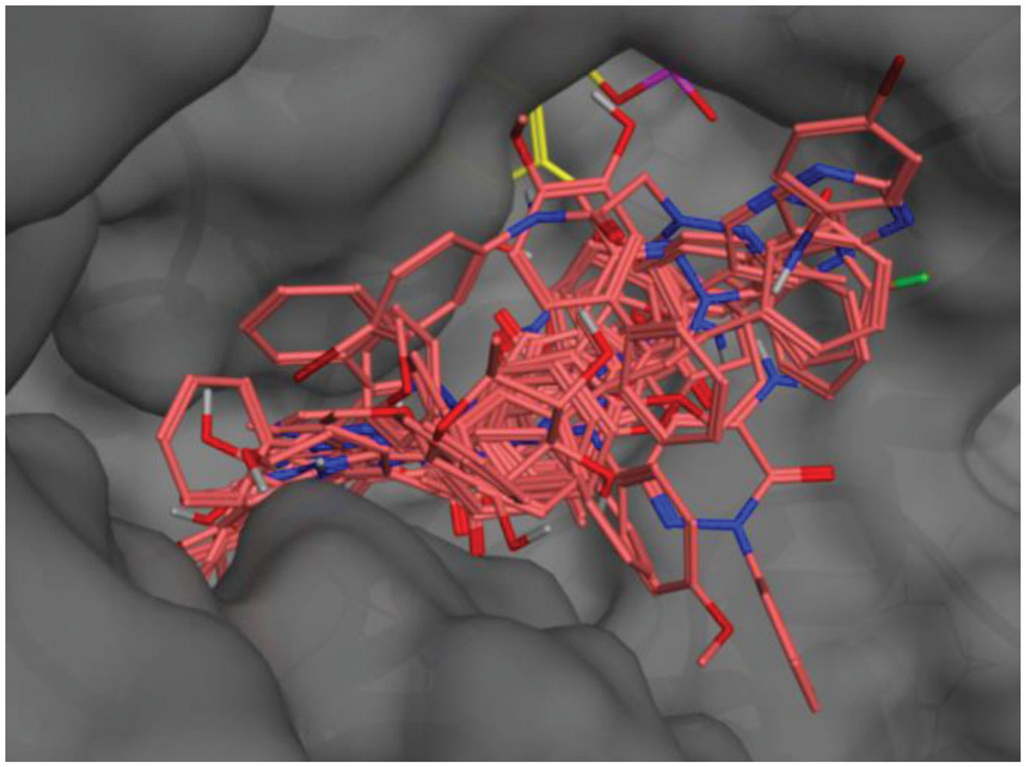
Binding poses of the full molecular series (dark pink) in the active site of 7AAT. The cofactor pyridoxal phosphate is reported in yellow.

We have used docking calculations to further characterise the activity of our set of molecules on AST. To this end, calculations were performed with Autodock Vina, a validated software for iVS structure-based applications^40^^,^^41^. Docking analysis of crystallised ligands, with an established binding mode, were carried out in order to obtain a minimum energy level which has been used as the cut-off for the assessment of binding energies of the new ligands. For the docking calculations, we adopted a previously reported methodology based on the crystal structures of AST (PDB ID: 7AAT) as the enzyme input files^37^. To validate our docking procedure, we have firstly docked two know inhibitors in the active site and, subsequently, we have compared the results with former data from the literature. Hesperetin and hesperidin (*K*_i_ of 51 and 682 µM, respectively)^37^ were docked in the AST active site (see Table 4 for calculated energies and binding efficiency; see Figure 1 for the best poses). Among the two calculated results – i.e. ligand efficiency (LE) and binding energy (BE) – the first value demonstrates linearity with the experimentally obtained data for both the hesperetin and hesperidin. Interestingly, all the 17 molecules analysed for repurposing have a predicted BE lower than that of hesperidin and analogue **3a** has predicted LE higher than hesperetin.

**Table 4.** Docking results for 7AAT.

Lig.	LE	BE[kcal/mol]
**1a**	0.2162	6.271
**2a**	0.2274	6.368
**3a**	0.3363	6.390
**5a**	0.2813	6.752
**7**	0.2791	6.699
**8**	0.2203	7.931
**9**	0.2156	7.760
**10b**	0.2583	7.233
**10g**	0.2792	6.421
**11**	0.2887	6.930
**13a**	0.2501	6.503
**15a**	0.2536	6.339
**15f**	0.2280	6.840
**17a**	0.2613	6.795
**20**	0.2311	7.164
**21a**	0.2718	7.068
**21d**	0.2539	6.602
hesperetin	0.3054	6.718
hesperidin	0.1831	7.875

The active site of AST is formed by Ser-107, Gly-108, Thr-109, Trp-140, His-143, His-189, Asp-194, Asp-222, Arg-224, Tyr-225, Ser-255, Phe-256, Ser-257, Lys-258, Phe-360, and Arg-386. The coenzyme PLP is covalently bonded to Lys-258 and interacts by hydrogen bonds with different residues (Trp-140, His-143, Asp-222, Tyr-225). Previous experimental and modelling studies indicate that the guanidinium groups of Arg-386 interact through hydrogen bonds and ionic interactions with the carboxylate groups of the substrate^42^. The best poses of each analysed molecule are reported in Figure S1-S17 (Supporting Information), indicating that most of the terms interact with the above-mentioned amino acids in the AST active site.

**Figure 2.**
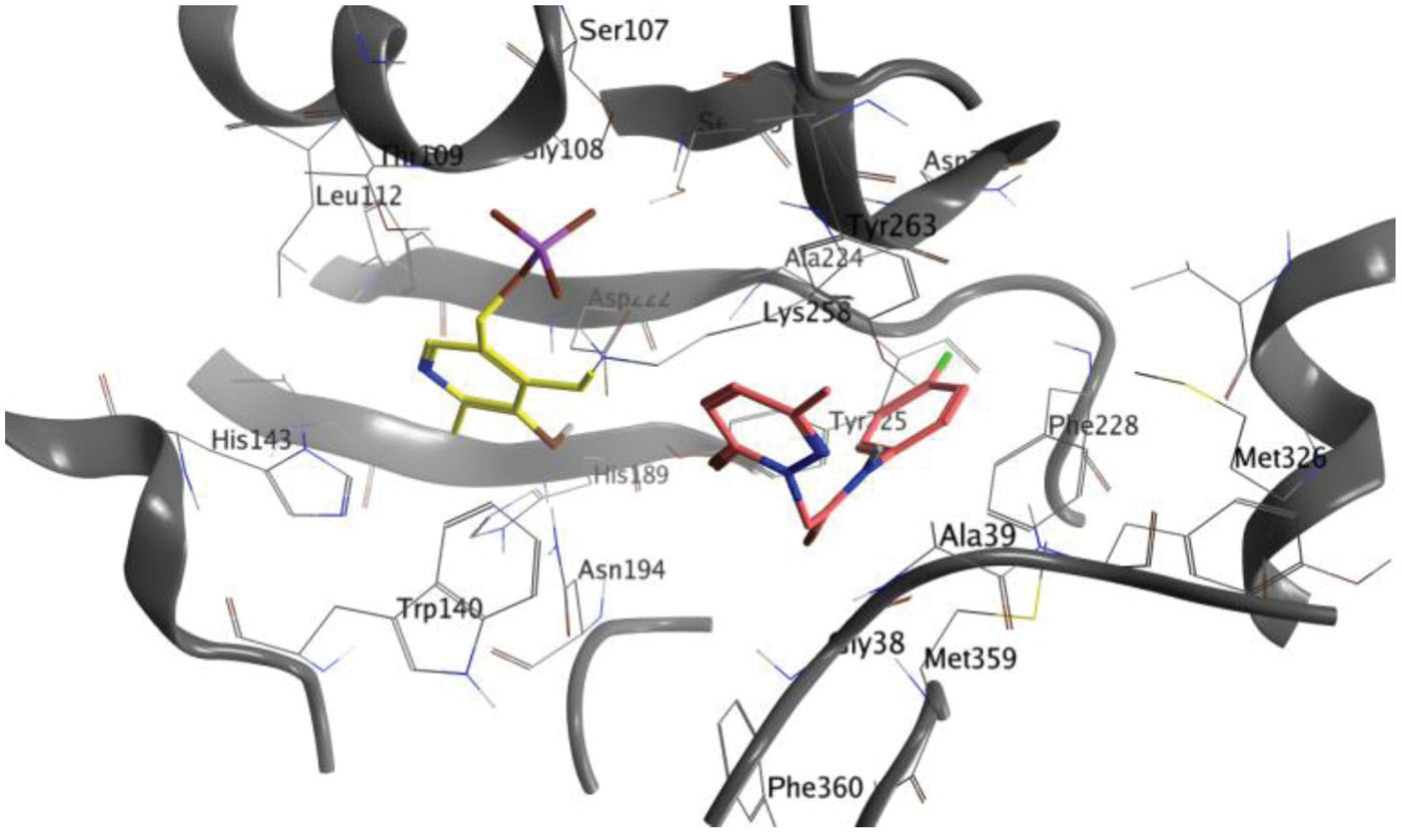
Binding of analogue 3a after 50 ns of MD.

**Figure 3.**
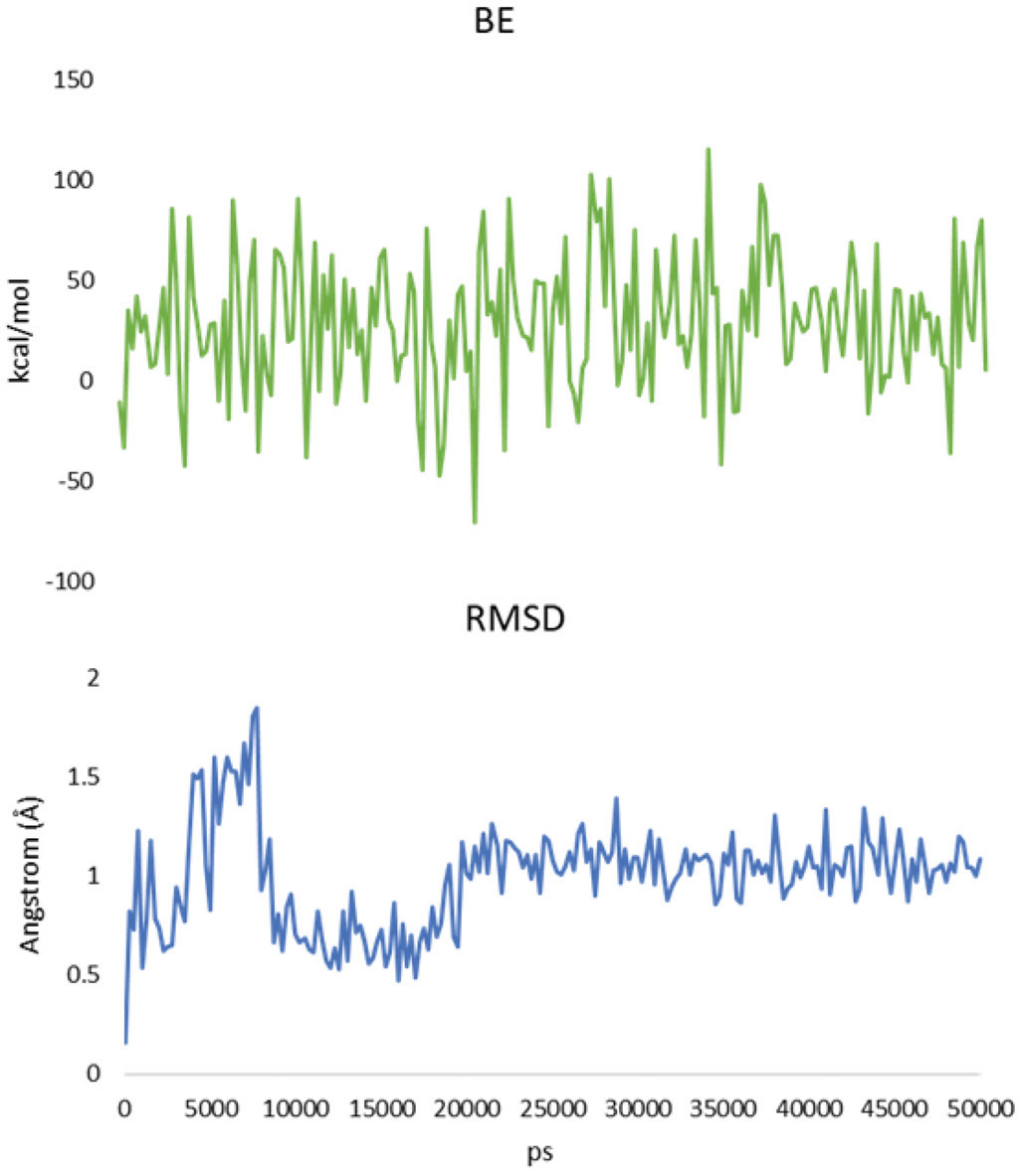
Up, binding energy (BE) of analogue **3a** during the MD simulation. Down, root-mean-square deviation of atomic positions (RMSD) of **3a** during the MD simulation. Time is expressed in ps.

An additional molecular dynamic (MD) simulation study (Figures S18–S26) was then conducted to verify the effectiveness and stability of the poses of the most promising compound (i.e. **3a**, with high LE). Specifically, we performed 50 ns MD simulations starting with the result of the docking calculations (see results in Figures 2 and 3). BE of the molecule result stable over 50 ns and the root-mean-square deviation of atomic positions (RMSD) is less than 2.0 Å. This suggests that after the first 20 ns **3a** presence is stabilised inside the binding pocket and confirms the finding of the docking calculations^43^^,^^44^. Moreover, the total potential energy of the system and the structure of the protein results stable over the time of simulation (Figure S18–S26, Supporting Information).

### 2.3. Admet assessment

**Figure 4.**
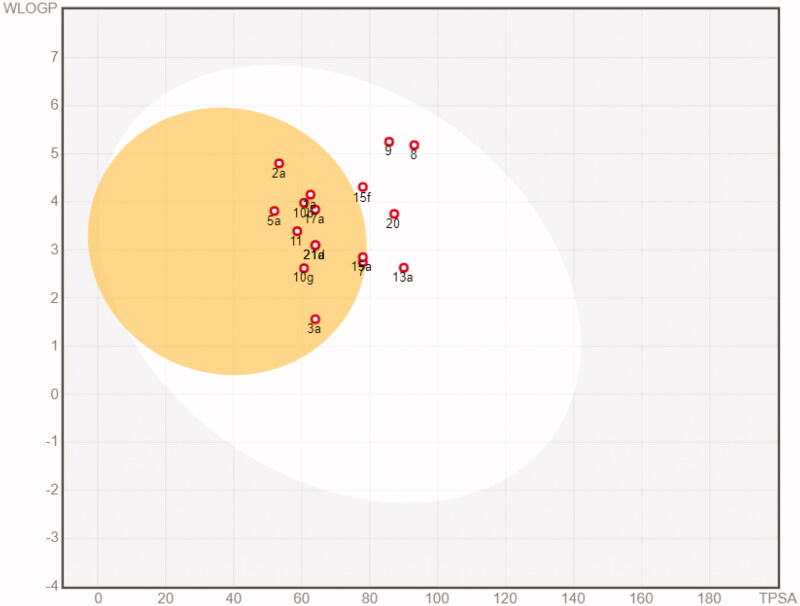
BOILED-Egg plot. Points located in the BOILED-Egg’s yellow represent the analogues predicted to passively permeate the BBB. Points in the egg white are relative to the analogues predicted to face passive absorption by the gastrointestinal tract. Red dots indicate that the molecules are predicted not to be affected by P-glycoprotein mediated extrusion from the CNS.

The *in silico* assessment has been expanded through the evaluation of pharmacokinetic profiles and possible adverse side effects for the representative 17 molecules. The ability to reach targets in bioactive form was assessed using the SwissADME (http://swissadme.ch)^45^ and pkCSM (http://biosig.unimelb.edu.au/pkcsm/)^46^ web platforms. SwissADME results are reported in Figures S27-S43 and pkCSM results are reported in Tables S19–S23. Most of the compounds resulted orally available and from moderately to soluble in water (with the exception of 8 and **9** that resulted instead poorly soluble). None of the compounds resulted a substrate of glycoprotein G (Pgp). Only compounds **8** and **9** violate the Lipinski rule of 5. Additional four drug-likeness rules, namely Ghose, Egan, Veber, and Muegee, were contextually satisfied by the majority of the analogues (with the exception of **8** and **9**, that do not satisfy the Ghose and Muegee rules). The result of the pan assay interference structures (designed to exclude molecules that are most likely to show false positives in biological assays) did not point out relevant issues. In parallel, the calculated absorption and distribution have been graphically represented by the Edan–Egg model reported in Figure 4 (Brain or IntestinaL EstimateD, BOILED-Egg). The visual analysis the Edan–Egg model highlights that most of the molecules are predicted to passively permeate the BBB, whereas compounds **8**, **9**, **13a**, **15f** and **20** can only be passively absorbed by the gastrointestinal tract.

With regard to the absorption parameters, the analogues present a promising oral availability due to the optimal Caco-2 cell permeability and intestinal absorption (>0.89 and >87%, respectively) (Table S19, Supporting Information). The calculated values of steady state volume of distribution are relatively low for **3a**, **7a**, **8**, **13a**, **15a**, **15f**, **17a**, **20a**, **21a** and **21d**. The whole data set shows a significant unbound fraction in the plasma, thus proposing availability to interact with the pharmacological target (Table S20). The calculated values of the total clearance (Table S22, Supporting Information) indicate the majority of the compounds have a good renal elimination and are not substrates of the renal organic cation transporter 2, with the exception of compounds **5a** and **10g**. Lastly, compounds **10g**, **15f**, **17a**, **20a**, **21a** and **21d** did not pass the AMES toxicity test, whereas all others did not present any relevant toxicity problem that could limit the use as drugs (Table S23, Supporting Information).

## 3. Materials and methods

### 3.1. Structure preparation and minimisation

The molecular structures of this study were built using Marvin Sketch (18.24, ChemAxon Ltd., Budapest, Hungary). A first molecular mechanics energy minimisation was used for 3 D structures created from the SMLES, and the Merck molecular force field (MMFF94) present in Marvin Sketch was used. The protonation states were calculated assuming a neutral pH. The PM3 Hamiltonian, as implemented in the MOPAC package (MOPAC2016, Stewart Computational Chemistry) was then used to further optimise the 3 D structures.

### 3.2. Molecular docking

Flexible ligand docking experiments were performed by employing AutoDock Vina software implemented in YASARA (v. 14.7.17, YASARA Biosciences, licenced to King’s College London), using the three-dimensional crystal structure of the Aspartate Aminotransferase in complex with the co-factor pyridoxal-5′-phosphate (PDB ID: 7AAT) obtained from the Protein Data Bank (PDB, http://www.rcsb.org/pdb). All the parameters were used at their default settings. The sequence in the structure, differently to the PDB ID: 5AX8, has the cofactor co-crystalized. The two structures have an overall RMSD: 0.817 Å, and an RMSD of 0.334 Å considering only the active site.

### 3.3. Molecular dynamics simulations

The molecular dynamics simulations of the aspartate aminotransferase/**3a** complex was performed with the YASARA Structure package using the three-dimensional crystal structure of the aspartate aminotransferase in complex with the co-factor pyridoxal-5′-phosphate (PDB ID: 7AAT). A periodic simulation cell with boundaries extending 5 Å from the surface of the complex was employed. The box was filled with water, with a maximum sum of all water bumps of 1.0 Å, and a density of 0.997 g/mL with explicit solvent. YASARA’s pKa utility was used to assign pKa values at pH 7.4, and the cell was neutralised with NaCl (0.9%) in these conditions. Water molecules were deleted to readjust the solvent density to 0.997 g/mL. The final system dimensions were approximately 71 × 79 × 69 Å. The ligand force field parameters were generated with the AutoSMILES utility, which employs semiempirical AM1 geometry optimisation and assignment of charges, followed by the assignment of the AM1BCC atom and bond types with refinement using the RESP charges and, lastly, the assignments of general AMBER force field atom types. Optimisation of the hydrogen bond network of the various enzyme–ligand complexes was obtained using the method established by Hooft *et al.* (see YASARA manual), to address ambiguities arising from multiple side-chain conformations and protonation states that are not well resolved in the electron density. A short MD was run on the solvent only. The entire system was then energy minimised using first a steepest descent minimisation to remove conformational stress, followed by a simulated annealing minimisation until convergence (<0.01 kcal/mol Å). The MD simulation was then initiated, using the NVT ensemble at 298 K and integration time steps for intramolecular and intermolecular forces every 1.25 fs and 2.5 fs, respectively. Finally, 50 ns MD simulations without any restrictions were conducted and the conformations of each system were recorded every 200 ps. The binding energy (BE; Figure 3) is calculated from the energy at an infinite distance by subtracting the energy of the system. More positive is the binding energy, more favourable is the interaction. The obtained binding energy values include solvation events according to the Poisson-Boltzmann approach (PBS). PBS uses a customised version of the APBS programme (see YASARA manual) to solve the Poisson-Boltzmann equation, yielding the electrostatic potential with implicit solvent and counter ions. Accordingly, the PBS binding energy is calculated through the following equation:

$$\text{BE}=E_{\text{potRecept}}+{\;E}_{\text{solvRecept}}+{\;E}_{\text{potLigand}}+{\;E}_{\text{solvLigand}}–{\;E}_{\text{potComplex}}–{\;E}_{\text{solvComplex}}$$

## 4. Conclusion

We have described here computational repurposing approaches for a newly synthesised series of pyridazinone-based small-molecules. The *in silico* screening has been performed by pharmacophore mapping and structure-based models. Our pharmacophore iVS adopted 16159 receptor-based pharmacophore models and the results highlighted aspartate aminotransferase as a valid target for the compound dataset. Furthermore, docking calculations and molecular dynamic simulations were also used to fully establish the suitability of aspartate aminotransferase as target for this molecular series. AST catalyses the reversible endogenous interchange of aspartate and α-ketoglutaric acid to glutamic acid and oxaloacetic acid. The inhibition of this enzyme has been also investigated for selective interference for the survival of breast cancer and other malignant cells over normal mammalian cells^39^^,^^47^. Clearly, novel inhibitors for this enzyme can be useful in anti- cancer studies and therapies. Biological experiments are ongoing to experimentally confirm the findings reported here and propose a number of these pyridazine-based analogues as new hit compounds for AST inhibition and linked pharmacological opportunities.

## Supplementary Material

Supplemental Material

## References

[1] Franzen RG. Recent advances in the preparation of heterocycles on solid support: a review of the literature. J Comb Chem 2000;2:195–214. 10827923 10.1021/cc000002f

[2] Dolle RE, Le Bourdonnec B, Morales GA, et al. Comprehensive survey of combinatorial library synthesis: 2005. J Comb Chem 2006;8:597–635. 16961395 10.1021/cc060095m

[3] Kaushik NK, Kaushik N, Attri P, et al. Biomedical importance of indoles. Molecules 2013;18:6620–62. 23743888 10.3390/molecules18066620 PMC6270133

[4] Macarron R, Banks MN, Bojanic D, et al. Impact of high-throughput screening in biomedical research. Nat Rev Drug Discov 2011;10:188–95. 21358738 10.1038/nrd3368

[5] Scannell JW, Blanckley A, Boldon H, Warrington B. Diagnosing the decline in pharmaceutical R&D efficiency. Nat Rev Drug Discov 2012;11:191–200. 22378269 10.1038/nrd3681

[6] Akhondzadeh S. The importance of clinical trials in drug development. Avicenna J Med Biotechnol 2016;8:151. 27920881 PMC5124250

[7] Paul SM, Mytelka DS, Dunwiddie CT, et al. How to improve R&D productivity: the pharmaceutical industry’s grand challenge. Nat Rev Drug Discov 2010;9:203–14. 20168317 10.1038/nrd3078

[8] Carta F, Angeli A, Nielsen C-T, et al. New biological targets for the treatment of leishmaniasis. In: Jayaprakash V, Castagnolo D, Özkay Y, ed. Medicinal chemistry of neglected and tropical diseases: advances in the design and synthesis of antimicrobial agents. Florida, US: Taylor & Francis Group, CRC Press; 2018.

[9] Shahreza ML, Ghadiri N, Mousavi SR, et al. A review of network-based approaches to drug repositioning. Brief Bioinform 2018;19:878–92. 28334136 10.1093/bib/bbx017

[10] Graul AI, Pina P, Cruces E, Stringer M. The year’s new drugs and biologics 2018: Part I. Drugs Today 2019;55:35–87. 10.1358/dot.2019.55.1.2959663 30740611

[11] Graul AI, Dulsat C, Pina P, et al. The year’s new drugs and biologics 2018: part II-news that shaped the industry in 2018. Drugs Today 2019;55:131–60. 10.1358/dot.2019.55.2.2974962 30816887

[12] Huang GH, Li JC, Wang P, Li WB. A review of computational drug repositioning approaches. Comb Chem High T Scr 2018;20:831–8. 10.2174/1386207321666171221112835 29268682

[13] Xue HQ, Li J, Xie HZ, Wang YD. Review of drug repositioning approaches and resources. Int J Biol Sci 2018;14:1232–44. 30123072 10.7150/ijbs.24612 PMC6097480

[14] Cilibrizzi A, Floresta G, Abbate V, Giovannoni MP. iVS analysis to evaluate the impact of scaffold diversity in the binding to cellular targets relevant in cancer. J Enz Inhib Med Chem 2019;34:44–50. 10.1080/14756366.2018.1518960 PMC6211261 30362379

[15] Austin ND, Sahinidis NV, Trahan DW. Computer-aided molecular design: an introduction and review of tools, applications, and solution techniques. Chem Eng ResDes 2016;116:2–26.

[16] Floresta G, Pittalà V, Sorrenti V, et al. Development of new HO-1 inhibitors by a thorough scaffold-hopping analysis. Bioorg Chem 2018;81:334–9. 30189413 10.1016/j.bioorg.2018.08.023

[17] Floresta G, Cilibrizzi A, Abbate V, et al. 3D-QSAR assisted identification of FABP4 inhibitors: an effective scaffold hopping analysis/QSAR evaluation. Bioorg Chem 2019;84:276–84.. 30529845 10.1016/j.bioorg.2018.11.045

[18] Giovannoni MP, Vergelli C, Cilibrizzi A, et al. Pyrazolo[1’,5’:1,6]pyrimido[4,5-d]pyridazin-4(3H)-ones as selective human A1 adenosine receptor ligands. Bioorg Med Chem 2010;18:7890–9. 20937560 10.1016/j.bmc.2010.09.043

[19] Biagini P, Biancalani C, Graziano A, et al. Functionalized pyrazoles and pyrazolo[3,4-d]pyridazinones: synthesis and evaluation of their phosphodiesterase 4 inhibitory activity. Bioorg Med Chem 2010;18:3506–17. 20413313 10.1016/j.bmc.2010.03.066

[20] Cilibrizzi A. Correspondence: compound 17b and formyl peptide receptor biased agonism in relation to cardioprotective effects in ischaemia-reperfusion injury. Nat Commun 2018;9:531. 29416027 10.1038/s41467-017-02654-2 PMC5803208

[21] Vergelli C, Schepetkin IA, Ciciani G, et al. 2-Arylacetamido-4-phenylamino-5-substituted pyridazinones as formyl peptide receptors agonists. Bioorg Med Chem 2016;24:2530–43. 10.1016/j.bmc.2016.04.019 PMC5055850 27134116

[22] Crocetti L, Vergelli C, Cilibrizzi A, et al. Synthesis and pharmacological evaluation of new pyridazin-based thioderivatives as formyl peptide receptor (FPR) agonists. Drug Dev Res 2013;74:259–71.

[23] Marchueta Hereu I, Serra Masia X. New process for preparing 3-methyl-4-phenylisoxazolo[3,4-d]pyridazin-7(6h)-one. WO2008107064A1, 2008. Available from: https://patents.google.com/patent/WO2008107064A1

[24] Dal Piaz V, Castellana MC, Vergelli C, et al. Synthesis and evaluation of some pyrazolo[3,4-d]pyridazinones and analogues as PDE 5 inhibitors potentially useful as peripheral vasodilator agents. J Enz Inhib Med Chem 2002;17:227–33. 10.1080/1475636021000008494 12530475

[25] Sprio V, Ajello E, Mazza A. Nitrogen heterocycles. II. Hydrogenation of isoxazolo[3,4-d]pyridazin-7-ones, isoxazolo[3,4-d]pyridazin-4-ones, and isoxazolo[3,4-d]pyridazine-4,7-diones. Ann Chim 1967;57:836–45.

[26] Giovannoni MP, Schepetkin IA, Cilibrizzi A, et al. Further studies on 2-arylacetamide pyridazin-3(2H)-ones: design, synthesis and evaluation of 4,6-disubstituted analogs as formyl peptide receptors (FPRs) agonists. Eur J Med Chem 2013;64:512–28. 23685570 10.1016/j.ejmech.2013.03.066 PMC3711119

[27] Dal Piaz V, Ciciani G, Giovannoni MP. Reductive cleavage of isoxazolo[3,4-d]pyridazinones: a synthetic approach to various 4,5-functionalized 3(2H)-pyridazinones. Heterocycles 1991;32:1173–9.

[28] Dal Piaz V, Ciciani G, Giovannoni MP. Synthesis and evaluation as platelet aggregation inhibitors of 6-phenyl-2, 4-substituted-3(2H)-pyridazinones and their rigid analogs benzo[h]cinnolin-3,5-diones. Drug Des Discov 2010;14:53–75. 8854045

[29] Giovannoni MP, Cesari N, Vergelli C, et al. Dal Piaz V. 4-Amino-5-substituted-3(2H)-pyridazinones as orally active antinociceptive agents: synthesis and studies on the mechanism of action. J Med Chem 2007;50:3945–53. 17629262 10.1021/jm070161e

[30] Giovannoni MP, Ciciani G, Cilibrizzi A, et al. Further studies on pyrazolo[1’,5’:1,6]pyrimido[4,5-d]pyridazin-4(3H)-ones as potent and selective human A1 adenosine receptor antagonists. Eur J Med Chem 2015;89:32–41. 25462223 10.1016/j.ejmech.2014.10.020

[31] Ciciani G, Dal Piaz V, Giovannoni MP. Synthesis and evaluation of in vitro antitumor activity of some substituted 5-pyridazinyl-styrylketones. Farmaco 2010;23:873–885. 1793472

[32] Dal Piaz V, Giovannoni MP, Vergelli C, Aguilar IN. Preparation of pyridazin-3(2H)-ones as phosphodiesterase 4 (PDE4) inhibitors. PCT Int. Appl. WO 2003097613 A1 20031127, 2003. Available from: https://patents.google.com/patent/WO2003097613A1/en

[33] Banoglu E, Akoğlu Ç, Ünlü S, et al. Synthesis of 2- [5,6-diphenyl-3(2H)-pyridazinone-2-yl]acetamide and 3- [5,6-diphenyl-3- (2H)-pyridazinone-2-yl]propanamide derivatives as analgesic and anti-inflammatory agents. Turk J Pharm Sci 2004;337: 7–70. 10.1002/ardp.200200738 14760622

[34] Dogruer DS, Sahin MF, Kuepeli E, Yesilada E. Synthesis and analgesic and anti-inflammatory activity of new pyridazinones. Turk J Chem 2003;27:727–38.

[35] Wang X, Shen Y, Wang S, et al. PharmMapper 2017 update: a web server for potential drug target identification with a comprehensive target pharmacophore database. Nucleic Acids Res 2017;45:W356–W360. 28472422 10.1093/nar/gkx374 PMC5793840

[36] Liu X, Ouyang S, Yu B, et al. PharmMapper server: a web server for potential drug target identification using pharmacophore mapping approach. Nucleic Acids Res 2010;38:W609–614. 20430828 10.1093/nar/gkq300 PMC2896160

[37] Zareei S, Boojar MMA, Amanlou M. Inhibition of liver alanine aminotransferase and aspartate aminotransferase by hesperidin and its aglycone hesperetin: an in vitro and in silico study. Life Sci 2017;178:49–55. 28385612 10.1016/j.lfs.2017.04.001

[38] Thornburg JM, Nelson KK, Clem BF, et al. Targeting aspartate aminotransferase in breast cancer. Breast Cancer Res 2008;10(5):R84. 18922152 10.1186/bcr2154 PMC2614520

[39] Anglin J, Zavareh RB, Sander PN, et al. Discovery and optimization of aspartate aminotransferase 1 inhibitors to target redox balance in pancreatic ductal adenocarcinoma. Bioorg Med Chem Lett 2018;28:2675–8. 29731362 10.1016/j.bmcl.2018.04.061 PMC6119644

[40] Lauro G, Masullo M, Piacente S, et al. Inverse virtual screening allows the discovery of the biological activity of natural compounds. Bioorg Med Chem 2012;20:3596–602. 22537682 10.1016/j.bmc.2012.03.072

[41] Lauro G, Romano A, Riccio R, Bifulco G. Inverse virtual screening of antitumor targets: pilot study on a small database of natural bioactive compounds. J Nat Prod 2011;74:1401–7. 21542600 10.1021/np100935s

[42] Nero TL, Wong MG, Oliver SW, et al. Aspartate aminotransferase: investigation of the active sites. J Mol Graph 1990;8:111–5. 2282353 10.1016/0263-7855(90)80091-s

[43] Liu K, Kokubo H. Exploring the stability of ligand binding modes to proteins by molecular dynamics simulations: a cross-docking study. J Chem Inf Model 2017;57:2514–22. 28902511 10.1021/acs.jcim.7b00412

[44] Liu K, Watanabe E, Kokubo H. Exploring the stability of ligand binding modes to proteins by molecular dynamics simulations. J Comput Aided Mol Des 2017;31:201–11. 28074360 10.1007/s10822-016-0005-2

[45] Daina A, Michielin O, Zoete V. SwissADME: a free web tool to evaluate pharmacokinetics, drug-likeness and medicinal chemistry friendliness of small molecules. Sci Rep 2017;7:42717. 28256516 10.1038/srep42717 PMC5335600

[46] Pires DE, Blundell TL, Ascher DB. pkCSM: Predicting small-molecule pharmacokinetic and toxicity properties using graph-based signatures. J Med Chem 2015;58:4066–72. 25860834 10.1021/acs.jmedchem.5b00104 PMC4434528

[47] Antti H, Sellstedt M. Metabolic effects of an aspartate aminotransferase-inhibitor on two T-cell lines. PLoS One 2018;13:e0208025. 30532126 10.1371/journal.pone.0208025 PMC6285999

