# Supplementary material for: Repurposing strategies on pyridazinone-based series by pharmacophore- and structure-driven screening
Source: J Enzyme Inhib Med Chem. 2020 May 5;35(1):1137–44. doi: 10.1080/14756366.2020.1760261 (PMC7241479; doi:10.1080/14756366.2020.1760261)
Supplement: Supplemental Material [file IENZ_A_1760261_SM3337.pdf]

Supporting Information

for

Repurposing strategies on pyridazinone-based series by pharmacophore- and structure-driven screening

G. Floresta,[a] L. Crocetti,[b] M. P. Giovannoni,[b] P. Biagini,[b] and A. Cilibrizzi[a,*]

[a]*Institute of Pharmaceutical Science, King's College London, Stamford Street, London SE1 9NH, UK*

[b]*NEUROFARBA, Pharmaceutical and Nutraceutical Section, University of Florence, Via Ugo Schiff, 6, 50019 Sesto Fiorentino (Fi), Italy*

Content:

1. Chemistry (**Scheme S1-S12**) 2

2. Experimental Section 11

3. Experimental Details - Molecular modelling 29

4. References 81

1. Chemistry

Analogues **1a,b** and **2a,b** were prepared from the 4,5-dichloro-3(2*H*)-pyridazinone **22**, commercially available (**Scheme S1**). *N*-2-benzyl derivatives **23a,b** were synthesised by treatment with 3- or 4-methoxybenzyl bromide in the presence of potassium carbonate and tetrabutylammonium bromide. The compounds **1a,b** were then obtained in two steps. The first reaction was a nucleophilic substitution leading to the selective displacement of the chlorine at C-5 of the pyridazinone ring of **23a,b**, using sodium methoxide in anhydrous methanol. The second step was the coupling of **24a,b** with 4-butoxyphenylboronic acid using tetrakis(triphenylphosphine)-palladium(0) catalyst under standard Suzuki conditions, to give the final 4-arylated-5-methoxy-pyridazinones **1a,b** in good yields. To synthesise analogues **2a,b**, it was not possible to obtain selective monoarylation through classical Suzuki reaction. Differently, a selective coupling of 4-butoxyphenylboronic acid on C-5 of the pyridazinone ring was achieved using *trans*-dichlorobis-(triethylphosphine)palladium (II) as catalyst and in the presence of 2M Na_2CO_3.

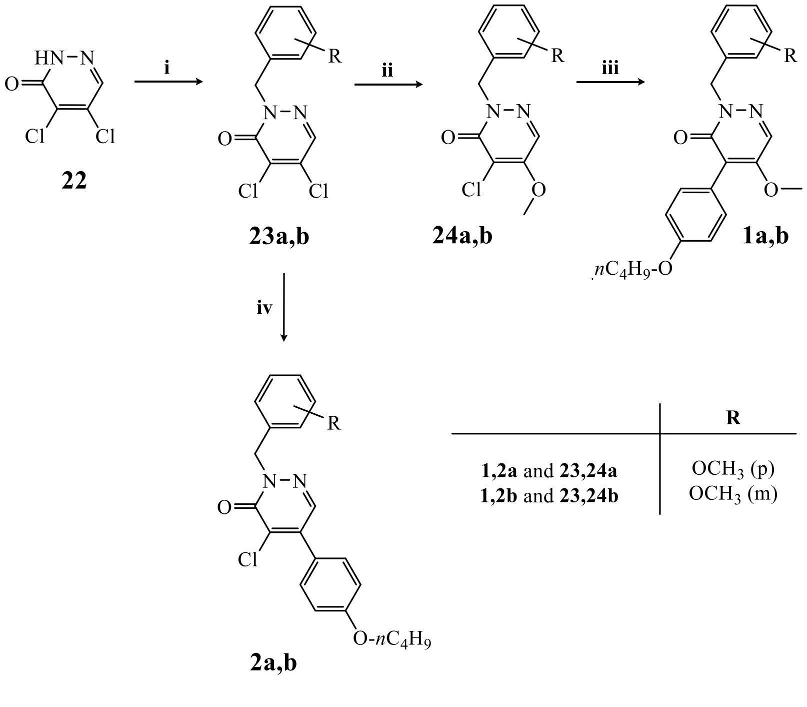

	R
**1,2a** and **23,24a**	OCH_3 (p)
**1,2b** and **23,24b**	OCH_3 (m)

Scheme S1. Reagents and conditions: i) 3 or 4-methoxybenzyl chloride (1.5 equiv), K_2CO_3 (2 equiv), Bu_4NBr (0.1 equiv), anhydrous CH_3CN, 5 h, reflux; **ii)** Na^0 (2 equiv), anhydrous CH_3OH, 1 h, rt; **iii)** 4-butoxyphenylboronic acid (3 equiv), $Pd(PPh_3)_4$ (0.03 equiv), 2M Na_2CO_3 in H_2O (1 equiv), toluene, 8 h, reflux; **iv)** 4-butoxyphenylboronic acid (0.5 equiv), $PdCl_2[(C_2H_5)_3P]_2$ (0.1 equiv), 2M Na_2CO_3 in H_2O (1 equiv), DMF, 6-12 h, rt.

In **Scheme S2** is depicted the synthesis of compounds **3a,b**. The pyridazinone scaffold **25** was converted into **26** through an alkylation with ethyl bromoacetate in standard condition. Intermediate

26 was subjected to alkaline hydrolysis with NaOH (**27**). The following amide bond formation on **27** using ethyl chloroformate and 4-bromoaniline through the mixed-anhydride method afforded compounds **3a,b**.

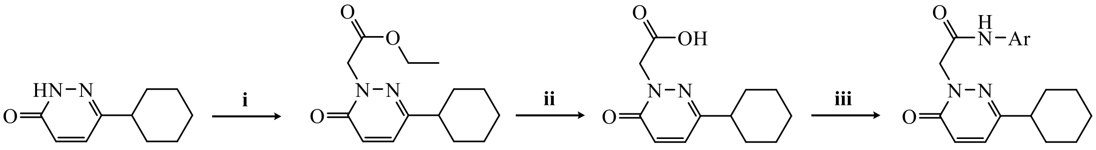

25 **26** **27** **3a,b**

	**Ar**
**3a**	C_6H_4-F (p)
**3b**	C_6H_4-3,4-methylenedioxy

Scheme S2. Reagents and conditions: i) ethyl bromoacetate (1.5 equiv), K_2CO_3 (2 equiv), anhydrous CH_3CN, 3 h, reflux; **ii)** NaOH 6N, 2 h, 80 °C; **iii)** ethyl chloroformate (1.1 equiv), Et_3N (3.5 equiv), suitable aniline (2 equiv), anhydrous THF, 12 h, -5 °C → rt.

The pyridazinone analogues **4a,b**, **5a,b** and **6a,b** (**Scheme S3**) were synthesised starting from the isoxazolo[3,4-d]pyridazinone **28**[23], which was firstly alkylated with the appropriate benzyl halide to give intermediates **29a,b**[18,24]. Oxidative ring cleavage of the intermediates **29a,b** with CAN in a mixture of nitric and acetic acids afforded 5-acetyl-4-nitropyridazinones **4a,b**[24] in moderate yields, through the selective opening of the five-member ring. Treatment of **4a,b** with HCl or HBr in acetone afforded the new 4-chloro (**5a,b**) and 4-bromo (**6a,b**) analogues in good yields, as the 4-nitro substituent behaves as an efficient leaving group, being easily replaced in mild conditions by halogen ions.

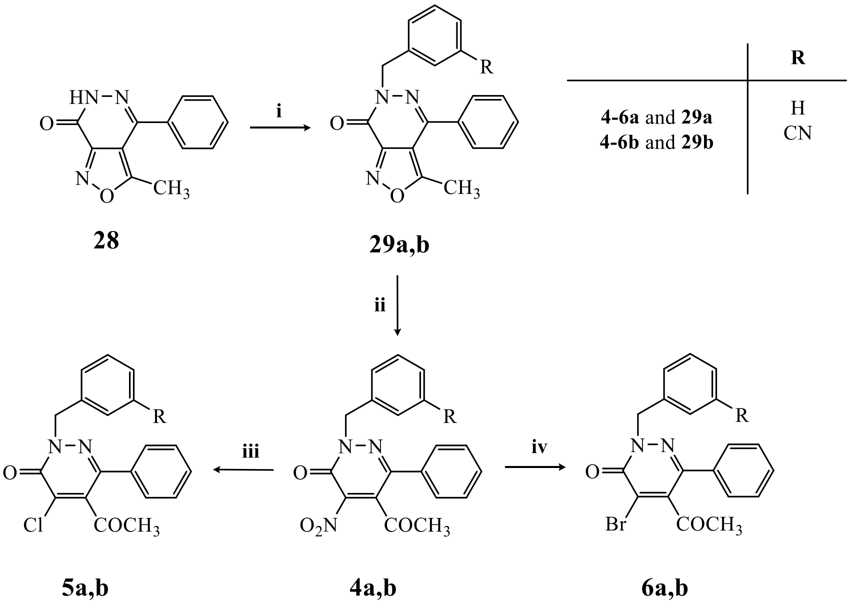

Scheme S3. Reagents and conditions: i) R-benzyl bromide (1.5 equiv), K_2CO_3 (2.0 equiv), anhydrous DMF, 1 h, 90 °C; **ii)** CAN (8.8 equiv), 50% AcOH, 65% HNO_3, 1 h, 55 °C; **iii)** 6M HCl, acetone, 5 h, 100 °C; **iv)** 47% HBr, acetone 2-3 h, 90 °C.

For the synthesis of compound **7** (**Scheme S4**) the starting material was the isoxazolo[4,3-*d*]pyridazinone **30**[25] (a structural isomer of **28**). This was alkylated to give intermediate **31** which, in turn, was converted into the final 4-acetyl-5-amino derivative **7** by reductive cleavage using ammonium formate as hydrogen source.

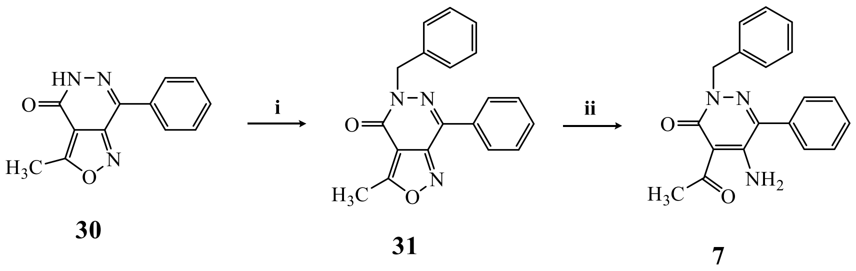

Scheme S4. Reagents and conditions: i) benzyl bromide (1.5 equiv), K_2CO_3 (2 equiv), anhydrous DMF, 40 min, 90 °C; **ii)** ammonium formate (4.3 equiv), 10% Pd/C (catalytic), ethanol, 30 min, reflux.

To synthesise the analogue **8** (**Scheme S5**), pyridazinone **32** was reacted with the commercially available 3-cyanobenzaldehyde through Knovenagel condensation. In this step, the basic reaction conditions determined the simultaneous hydration of the nitrile group to carboxylic acid (**33**). The intermediate **33** was alkylated with ethyl bromoacetate to give **34**, which afforded the bi-carboxylic

acid **35** after treatment with 6N NaOH at 60 °C. Compound **8** was obtained through the mixed-anhydride method using ethyl chloroformate and 4-bromoaniline.

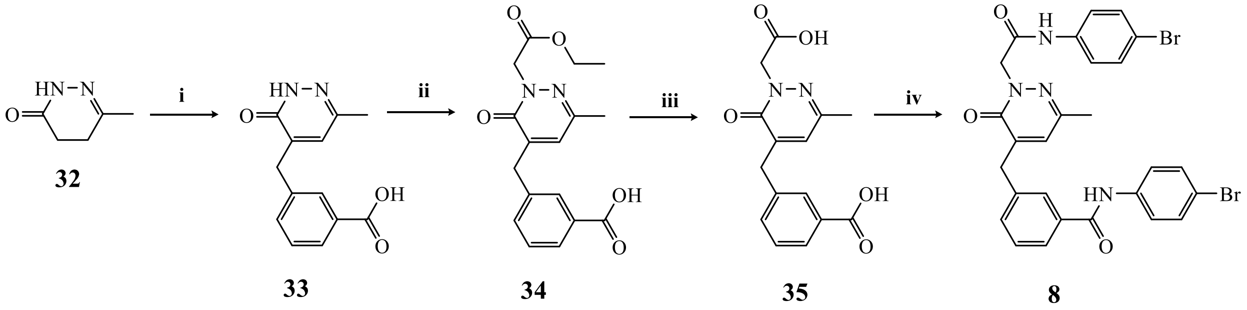

Scheme S5. Reagents and conditions: i) 3-cyanobenzaldehyde (2 equiv), KOH 5% (w/v) in anhydrous EtOH, 4 h, reflux; **ii)** ethyl bromoacetate (1.5 equiv), K_2CO_3 (2 equiv), anhydrous CH_3CN, 2 h, reflux; **iii)** 6N NaOH, 2 h, 60 °C; **iv)** ethyl chloroformate (1.1 equiv), Et_3N (3.5 equiv), 4-bromoaniline (2 equiv), anhydrous THF, 12 h, -5 °C → rt.

The synthetic pathway affording the analogue **9** is depicted in **Scheme S6**. To synthesise the biarylamine derivative **37**, compound **36**[26] was coupled with a two-fold excess of 4-methoxyphenylboronic acid in the presence of $Cu(OAc)_2$ and Et_3N. After standard hydrolysis of the ester group, **38** was processed to usual amidation reaction to afford the final compound **9**.

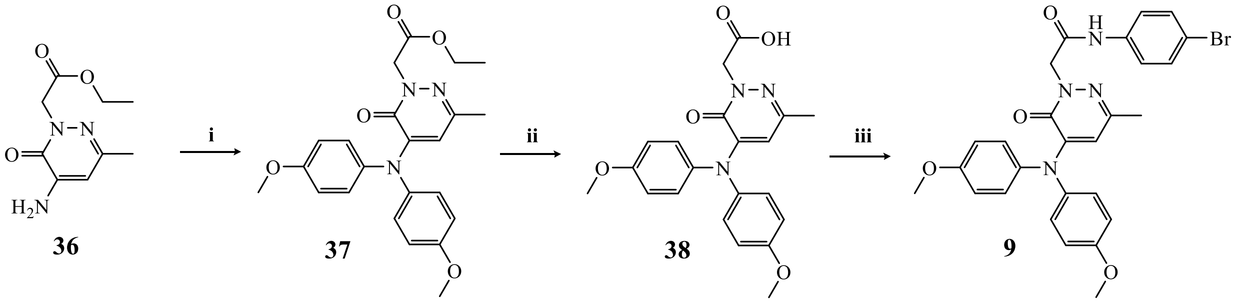

Scheme S6. Reagents and conditions: i) 4-methoxyphenylboronic acid (2 equiv), $Cu(OAc)_2$ (1.5 equiv), Et_3N (2 equiv), CH_2Cl_2, 12 h, rt; **ii)** 6N NaOH, 1.5 h, 80 °C; **iii)** ethyl chloroformate (1.1 equiv), Et_3N (3.5 equiv), 4-bromoaniline (2 equiv), anhydrous THF, 12 h, -5 °C → rt.

To obtain the final compounds **10a-g**, **11** and **12** (see **Scheme S7**), the pyridazinones **32**,**39**[26] were reacted with the appropriate (hetero)arylaldehyde through Knovenagel condensation (intermediates **33** and **40a-g**). The subsequent alkylation with bromoethane and potassium carbonate in anhydrous acetonitrile led to the final compounds **10a-g**. The dehydration with $POCl_3$ at reflux of products **10a** and **10c** afford the final compounds **11** and **12**.

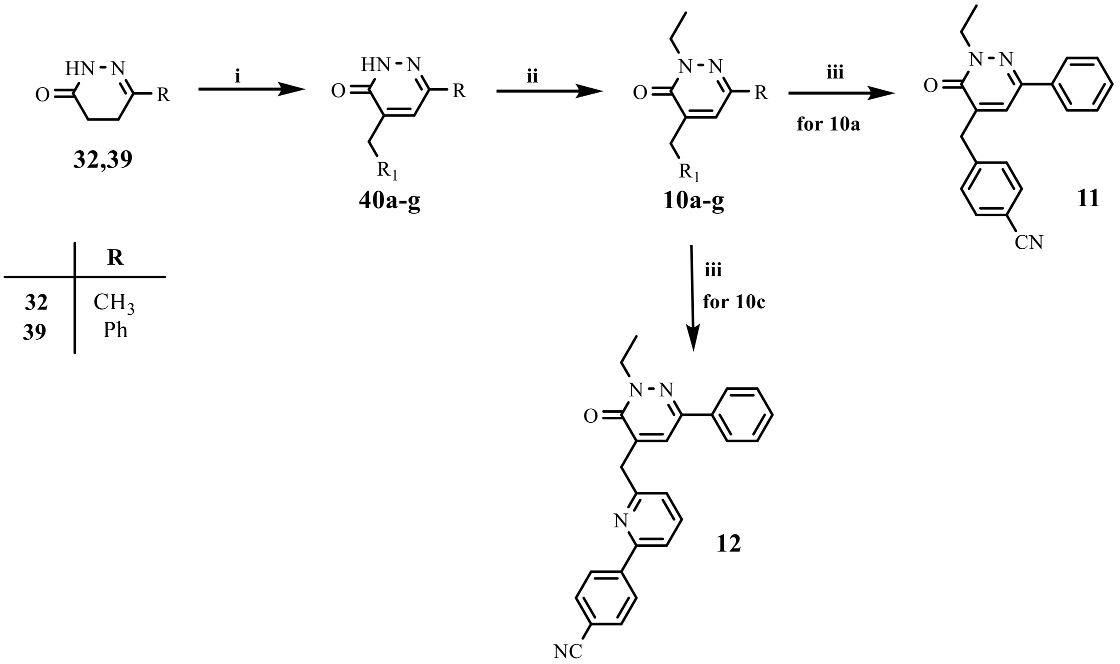

Comp.	R	R1	Comp.	R	R1
**40a/10a**	Ph	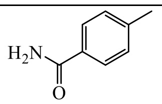	**40e/10e**	Ph	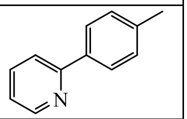
**40b/10b**	Ph	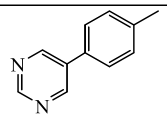	**40f/10f**	CH_3	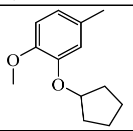
**40c/10c**	Ph	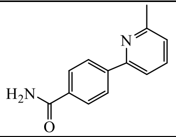	**40g/10g**	CH_3	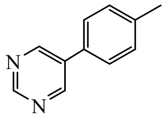
**40d/10d**	Ph	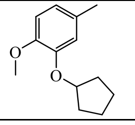			

Scheme S7. Reagents and conditions: i) appropriate (hetero)arylaldehyde (1 equiv), KOH 5% (w/v) in anhydrous EtOH, 4 h, reflux; **ii)** bromoethane (2 equiv), K_2CO_3 (1.5 equiv), anhydrous CH_3CN, 4-6 h, reflux; **iii)** $POCl_3$, 60°C, 2 h.

In the **Scheme S8** is reported the synthetic pathway to obtain the compounds **13a-c**. Starting from 4,6-diphenyl-isoxazol[3,4-d]pyridazin-7(6H)-one (**41**[27]), the reaction with the appropriate amine (for products **13a,b**) or cyclohexanol (for **13c**) in 1,4-dioxane, carried out in closed tube at 90°C, induced an opening of the isoxazole nucleus followed by the formation of the amide, or ester, at position 5 of the pyridazinone scaffold (**13a-c**).

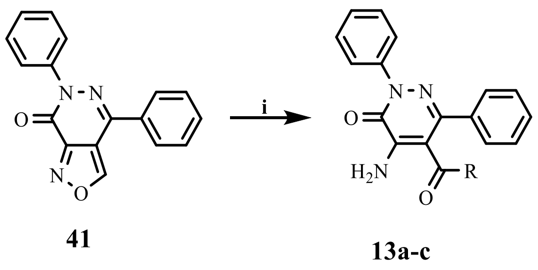

13	R
**a**	$NHnC_3H_7$
**b**	piperidine
**c**	OcC_6H_{11}

Scheme S8. Reagents and conditions: i) Suitable amine or alchol (3.5 equiv), 1,4-dioxane, 90 °C, 2-3 h.

For the synthesis of compound **14a-c** and **15a-l** (**Scheme S9**), the starting materials were the appropriate isoxazolo[4,3-*d*]pyridazinones **43a-g**[27-29]. The isoxazolopyridazinone **43e** is obtained for alkylation reaction of **42**[30] with bromopropane and K_2CO_3 in acetone at reflux. The formation of the styryl derivatives **44a-m** (**44a**[31]) was performed by using the appropriate (hetero)arylaldehyde and MeONa in methanol. The opening of the isoxazol ring (for compounds **44a**, **44b** and **44m**) with molybdenumhexacarbonyl in CH_3CN at reflux gave the acryloyl derivatives **14a-c** and afterwards the products **14a,b** were reduced with ammonium formate and Pd/C in ethanol to obtain the final compounds **15a,b**, respectively. Indeed, the same reduction (ammonium formate and Pd/C) starting from the other styryl derivatives **44** furnished directly the final compounds **15c-l** through a reduced opening.

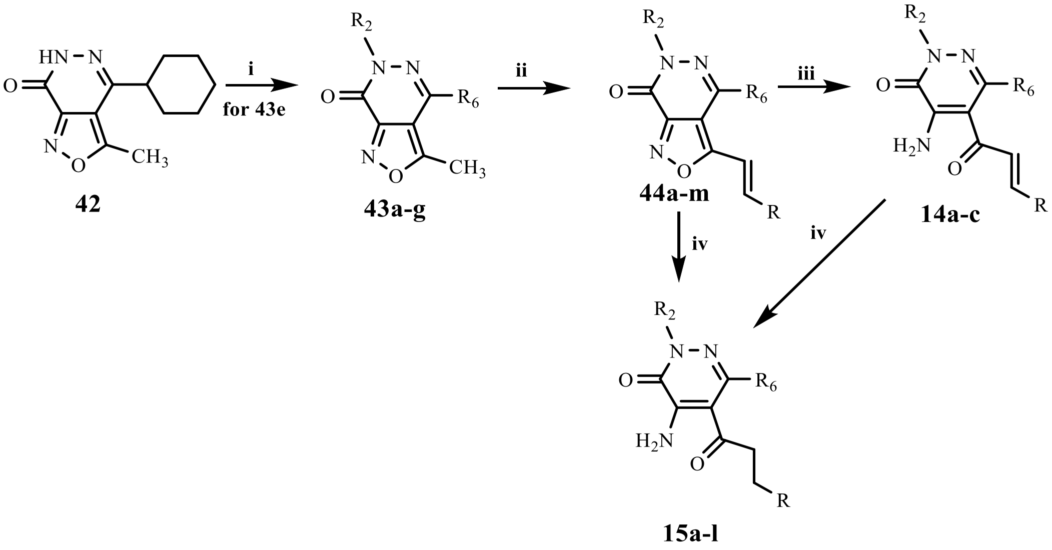

43	R2	R6
**a**	CH_3	Ph
**b**	$CH_2CH_2CH_3$	Ph
**c**	$CH_2CH_2CH_2CH_3$	Ph
**d**	$CH(CH_3)_2$	Ph
**e**	$CH_2CH_2CH_3$	cC_6H_{11}
**f**	Ph	Ph
**g**	CH_3	4-F-Ph

Comp.	R2	R6	R
**44a/14a/15a**	CH_3	Ph	Ph
**44b/14b/15b**	$CH_2CH_2CH_3$	Ph	Ph
**44c/15c**	$CH_2CH_2CH_2CH_3$	Ph	Ph
**44d/15d**	$CH(CH_3)_2$	Ph	Ph
**44e/15e**	$CH_2CH_2CH_3$	cC_6H_{11}	Ph
**44f/15f**	Ph	Ph	Ph
**44g/15g**	CH_2CH_3	Ph	Naphtalene
**44h/15h**	$CH_2CH_2CH_3$	Ph	Thiophene
**44i/15i**	$CH_2CH_2CH_3$	Ph	Naphtalene
**44l/15l**	$CH_2CH_2CH_3$	Ph	Pyridine
**44m/14c**	CH_3	4-F-Ph	Ph

Scheme S9. Reagents and Conditions: i) bromopropane (5 equiv), K_2CO_3 (2 equiv), acetone, reflux, 5-6 h; **ii)** suitable (hetero)arylaldehyde (2.5 equiv), MeONa (1.2 equiv), anhydrous MeOH, reflux, 2-20 min**; iii)** CH_3CN, H_2O (gt), $Mo(CO)_6$ (1.3 equiv), reflux, 3 h**; iv)** $HCOONH_4$ (2.5-3 equiv), Pd/C (cat.), EtOH abs., 80 °C, 2 h.

The **Scheme S10** shows the synthetic procedure for pyridazinone derivatives **16a,b** and **17a-c**, starting from the precursors **43f**[27] and **46b**, the latter obtained by a cyclization reaction of compound **45**[32] with polyphosphoric acid and ethanol under heat. The reduction and opening of isoxazolo[3,4-d]pyridazinone nucleus with ammonium formate and Pd/C afforded the compounds **47a,b** (**47a**[27]). Afterward, the deacetylation of **47a,b** with HBr at heat gave the final compounds **16a,b** which were subsequently treated with the opportune anhydride in pyridine to obtain the compounds **17a-c**.

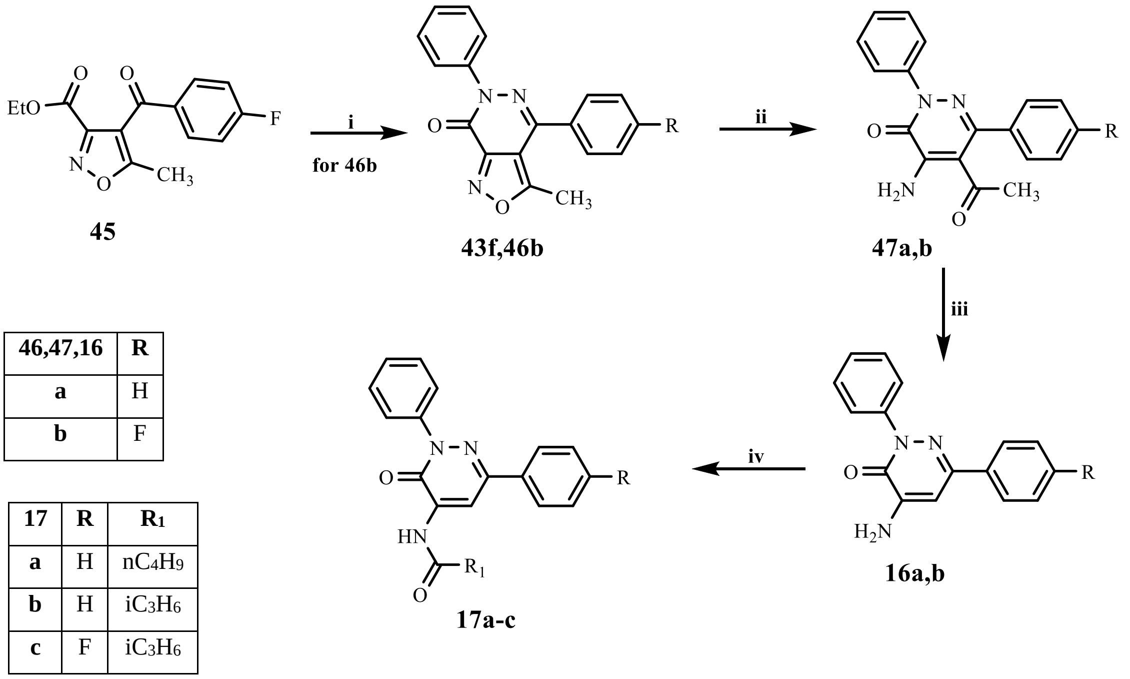

46,47,16	R
**a**	H
**b**	F

17	R	R1
**a**	H	nC_4H_9
**b**	H	iC_3H_6
**c**	F	iC_3H_6

Scheme S10. Reagents and Conditions: i) Phenylhydrazine (2 equiv), PPA (excess), EtOH abs., 80-90 °C, 1 h and 30 min; **ii)** $HCOONH_4$ (2.5 equiv), Pd/C (cat.), EtOH abs., 80 °C, 2 h; **iii)** HBr 48% (28.5 equiv), 130 °C, 2 h; **iv)** suitable anhydride (33.5 equiv), pyridine, sealed tube, 140 °C, 3-5 h.

The derivatives **18**, **19** and **20** were prepared from the 4-phenylisoxazolo[3,4-d]pyridazin-7(6H)-one **48**[27] (**Scheme S11**). The treatment of **48** with tert-butylamine in anhydrous 1,4-dioxane in a sealed tube gave the product **49** and the subsequent alkylation with benzyl chloride in the presence of potassium carbonate in acetone yielded the desired compound **18**. Indeed, the reaction of **48** with benzyl alcohol in presence of triethylamine gave the product **19** which was alkylated in the same conditions reported above to obtain the *N*-2-benzyl derivative **20**.

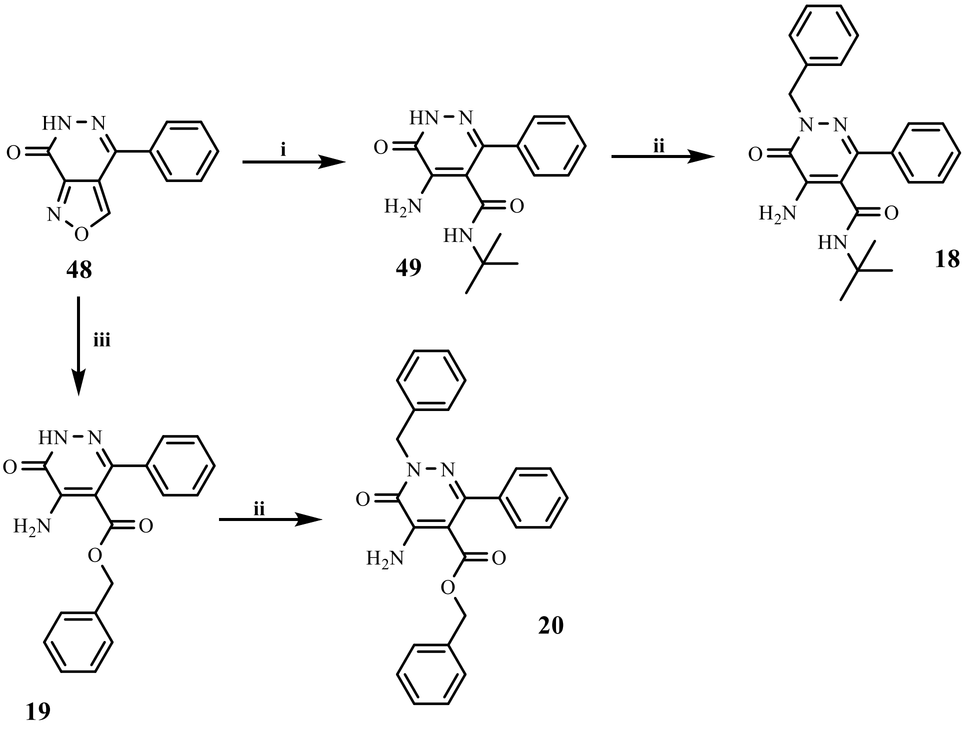

Scheme S11. Reagents and Conditions: i) *tert*-Butylamine (3 equiv), anhydrous 1,4-dioxane, 80-90 °C, 2 h; **ii)** Benzyl chloride (1.2 equiv), K_2CO_3 (2 equiv), anhydrous acetone, reflux, 2 h; **iii)** Benzyl alcohol (10 equiv), Et_3N (2.5 equiv), sealed tube, 80 °C, 3 h.

In **Scheme S12** is depicted the synthesis of compounds **21a-e**. Starting from the diphenylpyridazin-1(6*H*)-acetic acid **50a,b** (**50a**[33] and **50b**[34]), derivatives **21a-e** are obtained through the mixed-anhydride method using ethyl chloroformate and the appropriate amine (propylamine, isopropylamine or cyclopentylamine).

50	R_1	R_2
**a**	H	Ph
**b**	Ph	H

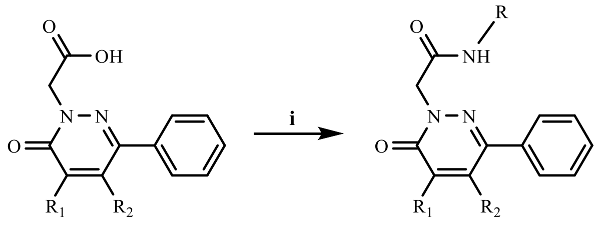

50a,b

21a-e

21	R_1	R_2	R
**a**	H	Ph	nC_3H_7
**b**	H	Ph	iC_3H_7
**c**	H	Ph	cC_5H_9
**d**	Ph	H	nC_3H_7
**e**	Ph	H	iC_3H_7

Scheme S12. Reagents and Conditions: i) ethyl chloroformate (1.1 equiv), Et_3N (3.5 equiv), suitable amine (2 equiv), anhydrous THF, 12 h, -5 °C → r.t.

2. Experimental Section

2.1. General remarks

Reagents and starting materials were obtained from commercial sources. Extracts were dried over Na_2SO_4, and the solvents were removed under reduced pressure. All reactions were monitored by thin layer chromatography (TLC) using commercial plates pre-coated with Merck silica gel 60 F-254. Visualisation was performed by UV fluorescence (λ_{max} = 254 nm) or by staining with iodine or potassium permanganate. Chromatographic separations were performed on a silica gel column by gravity (Kieselgel 40, 0.063-0.200 mm; Merck) or flash chromatography (Kieselgel 40, 0.040-0.063 mm; Merck). Yields refer to chromatographically and spectroscopically pure compounds, unless otherwise stated. When reactions were performed in anhydrous conditions, the mixtures were maintained under nitrogen atmosphere. Compounds were named following IUPAC rules as applied by Beilstein-Institut AutoNom 2000 (4.01.305) or CA Index Name. The identity and purity of intermediates and final compounds was ascertained through TLC chromatography, NMR and mass spectrometry. ^{1}H NMR spectra were recorded with Avance 400 instruments (Bruker Biospin Version 002 with SGU). Chemical shifts (δ) are reported in ppm to the nearest 0.01 ppm, using the solvent as internal standard. Coupling constants (*J* values) are given in Hz and were calculated using 'TopSpin 1.3' software rounded to the nearest 0.1 Hz. Data are reported as follows: chemical shift, multiplicity [exch, exchange; br, broad; s, singlet; d, doublet; t, triplet; q, quartet; quin, quintet; sext, sextet; sept, septet; m, multiplet; or as a combination of these (e.g. dd, dt *etc.*)], integration, assignment and coupling constant(s). Mass spectra (m/z) were recorded on ESI-TOF mass spectrometer (Bruker

Micro TOF) and reported mass values are within the error limits of ± 5 ppm mass units. All melting points were determined on a microscope hot stage Büchi apparatus and are uncorrected.

2.2. Chemistry

General Procedure for 23a,b. K_2CO_3 (6.06 mmol) and tetrabutylammonium bromide (0.30 mmol) were added to a stirred solution of 4,5-dichloro-3(2*H*)-pyridazinone **22** (3.03 mmol) in anhydrous acetonitrile (3 mL). 3- or 4-methoxybenzyl chloride (4.54 mmol) was added to the mixture and the reaction was carried out at reflux for 5 h. The mixture was then allowed to cool down and the solvent was evaporated *in vacuo*. Ice-cold water was added to the residue. After 1 h stirring in ice-bath, compounds **11a,b** were filtered off and recrystallised from ethanol.

4,5-Dichloro-2-(4-methoxybenzyl)pyridazin-3(2H)-one (23a). Yield = 81 %; mp = 116-117 °C (EtOH). ^{1}H NMR ($CDCl_3$) δ 3.81 (s, 3H, OCH_3), 5.28 (s, 2H, NCH_2), 6.88 (d, 2H, Ar, J = 8.6 Hz), 7.42 (d, 2H, Ar, J = 8.6 Hz), 7.79 (s, 1H, pyridaz).

4,5-Dichloro-2-(3-methoxybenzyl)pyridazin-3(2H)-one (23b). Yield = 60 %; mp = 80-82 °C (EtOH). ^{1}H NMR ($CDCl_3$) δ 3.82 (s, 3H, OCH_3), 5.31 (s, 2H, NCH_2), 6.87 (dd, 1H, Ar, J = 5.6 Hz, J = 2.6 Hz), 6.99-7.05 (m, 2H, Ar), 7.27 (t, 1H, Ar, J = 3.6 Hz), 7.80 (s, 1H, pyridaz).

General Procedure for 24a,b. Compounds **23a** or **23b** (0.88 mmol) was added to a stirred solution of Na^0 (1.76 mmol) in 3 mL of anhydrous methanol. The reaction mixture was stirred for 1 h at room temperature. After removal of the solvent *in vacuo*, ice-cold water was added to the residue and the precipitate was filtered off by suction and purified by crystallisation from ethanol.

4-Chloro-5-methoxy-2-(4-methoxybenzyl)pyridazin-3(2H)-one (24a). Yield = 53 %; mp = 135-137 °C (EtOH). ^{1}H NMR ($CDCl_3$) δ 3.80 (s, 3H, C_6H_4-OCH_3), 4.06 (s, 3H, OCH_3 pyridaz.), 5.31 (s, 2H, NCH_2), 6.87 (d, 2H, Ar, J = 8.6 Hz), 7.42 (d, 2H, Ar, J = 8.6 Hz), 7.80 (s, 1H, pyridaz).

4-Chloro-5-methoxy-2-(3-methoxybenzyl)pyridazin-3(2H)-one (24b). Yield = 60 %; mp = 80-82 °C (EtOH). ^{1}H NMR ($CDCl_3$) δ 3.81 (s, 3H, C_6H_4-OCH_3), 4.07 (s, 3H, OCH_3 pyridaz.), 5.34 (s, 2H, NCH_2), 6.85 (dd, 1H, Ar, J = 6.2 Hz, J = 1.89 Hz), 6.99 (s, 1H, Ar), 7.03 (d, 1H, Ar, J = 7.3 Hz), 7.26 (t, 1H, Ar, J = 7.9 Hz), 7.83 (s, 1H, pyridaz).

General Procedure for 1a,b. Na_2CO_3 (1.42 mmol, 2 M in H_2O) was added to the suspension of **24a** or **24b** (0.71 mmol), $Pd(PPh_3)_4$ [tetrakis (triphenylphosphine)palladium(0)] (0.02 mmol) and 4-butoxyphenylboronic acid (1.07 mmol) in toluene (2 mL). The mixture was stirred at reflux for 2 h. Extra 4-butoxyphenylboronic acid (1.07 mmol) was added and the reaction was refluxed for

additional 6 h. The solvent was evaporated under *vacuum* and the suspension was diluted with ice-cold water. After extraction with CH_2Cl_2, the organic layer was dried over Na_2SO_4 and the residue was purified by flash column chromatography using cyclohexane/ethyl acetate 3:1 as eluent.

4-(4-Butoxyphenyl)-5-methoxy-2-(4-methoxybenzyl)pyridazin-3(2H)-one (1a). Yield = 82 %; colorless oil. ^{1}H NMR ($CDCl_3$) δ 0.99 (t, 3H, CH_2*CH_3*, J = 7.4 Hz), 1.51 (sext, 2H, *CH_2*CH_3, J = 7.6 Hz), 1.79 (quin, 2H, CH_2*CH_2*CH_2, J = 6.9 Hz), 3.80 (s, 3H, C_6H_4-O*CH_3*), 3.89 (s, 3H, OCH_3 pyridaz.), 4.01 (t, 2H, OCH_2, J = 6.5 Hz), 5.31 (s, 2H, NCH_2), 6.87 (d, 2H, Ar, J = 8.6 Hz), 6.94 (d, 2H, Ar, J = 8.8 Hz), 7.48 (q, 4H, Ar, J = 8.0 Hz), 7.89 (s, 1H, pyridaz.).

4-(4-Butoxyphenyl)-5-methoxy-2-(3-methoxybenzyl)pyridazin-3(2H)-one (1b). Yield = 28 %; colorless oil. ^{1}H NMR ($CDCl_3$) δ 0.99 (t, 3H, CH_2*CH_3*, J = 7.4 Hz), 1.51 (sext, 2H, *CH_2*CH_3, J = 7.4 Hz), 1.79 (quin, 2H, CH_2*CH_2*CH_2, J = 6.9 Hz), 3.81 (s, 3H, C_6H_4-O*CH_3*), 3.90 (s, 3H, OCH_3 pyridaz.), 4.00 (t, 2H, OCH_2, J = 6.5 Hz), 5.35 (s, 2H, NCH_2), 6.84 (dd, 1H, Ar, J = 6.0 Hz, J = 2.3 Hz), 6.94 (d, 2H, Ar, J = 8.7 Hz), 7.02 (s, 1H, Ar), 7.06 (d, 1H, Ar, J = 7.6 Hz), 7.26 (t, 1H, Ar, J = 8.0 Hz), 7.51 (d, 2H, Ar, J = 8.7 Hz), 7.91 (s, 1H, pyridaz).

General Procedure for 2a,b. Na_2CO_3 (0.53 mmol, 2 M in H_2O) was added to a suspension of **23a** or **23b** (0.53 mmol), $PdCl_2[(C_2H_5)_3P]_2$ [trans-dichlorobis(triethylphosphine)palladium(II)] (0.05 mmol) and 4-butoxyphenylboronic acid (0.26 mmol) in DMF (2 mL). The mixture was stirred at room temperature for 12 h, diluted with ice-cold water and extracted with CH_2Cl_2. The organic layer was dried over Na_2SO_4 and the residue was purified by flash column chromatography using CH_2Cl_2 (for **2a**) and CH_2Cl_2/MeOH/NH_4OH 99:1:0.1 (for **2b**) as eluents.

5-(4-Butoxyphenyl)-4-chloro-2-(4-methoxybenzyl)pyridazin-3(2H)-one (2a). Yield = 22 %; mp = 84-85 °C (EtOH). ^{1}H NMR ($CDCl_3$) δ 1.01 (t, 3H, CH_2*CH_3*, J = 7.4 Hz), 1.53 (sext, 2H, *CH_2*CH_3, J = 7.6 Hz), 1.82 (quin, 2H, CH_2*CH_2*CH_2, J = 6.8 Hz), 3.81 (s, 3H, OCH_3), 4.03 (t, 2H, OCH_2, J = 6.5 Hz), 5.34 (s, 2H, NCH_2), 6.90 (d, 2H, Ar, J = 8.4 Hz), 7.01 (d, 2H, Ar, J = 8.6 Hz), 7.48 (dd, 4H, Ar, J = 5.9 Hz, J = 8.6 Hz), 7.78 (s, 1H, pyridaz).

5-(4-Butoxyphenyl)-4-chloro-2-(3-methoxybenzyl)pyridazin-3(2H)-one (2b). Yield = 14 %; mp = 73-75 °C (EtOH). ^{1}H NMR ($CDCl_3$) δ 1.01 (t, 3H, CH_2*CH_3*, J = 7.4 Hz), 1.53 (sext, 2H, *CH_2*CH_3, J = 7.5 Hz), 1.82 (quin, 2H, CH_2*CH_2*CH_2, J = 7.0 Hz), 3.83 (s, 3H, OCH_3), 4.04 (t, 2H, OCH_2, J = 6.5 Hz), 5.38 (s, 2H, NCH_2), 6.88 (dd, 1H, Ar, J = 5.7 Hz, J = 2.6 Hz), 7.02 (d, 2H, Ar, J = 8.8 Hz), 7.07 (s, 1H,Ar), 7.10 (d, 1H, Ar, J = 7.6 Hz), 7.29 (t, 1H, Ar, J = 8.0 Hz), 7.47 (d, 2H, Ar, J = 8.7 Hz), 7.79 (s, 1H, pyridaz).

Ethyl-2-[3-cyclohexyl-6-oxopyridazin-1(6H)-yl]acetate (26). A mixture of **25** (2.27 mmol), K_2CO_3 (4.54 mmol) and ethyl bromoacetate (3.41 mmol) in CH_3CN (3 mL) was refluxed under stirring for 3 h. The mixture was then concentrated *in vacuo*, diluted with cold water and extracted with CH_2Cl_2 (3 x 15 mL). The organic layer was evaporated and intermediate **26** was used in the following reaction without further purification. Yield ~ 100 %; oil. ^{1}H NMR ($CDCl_3$) δ 1.28-1.46 (m, 8H, (2 x CH_2 + CH-*H*) cyclohexyl + CH_2*CH_3*), 1.71-1.86 (m, 5H, 2 x CH_2 + CH-*H* cyclohexyl), 2.51-2.60 (m, 1H, CH, cyclohexyl), 4.23 (dt, 2H, *CH_2*CH_3, J = 4.3 Hz, J = 1.4 Hz), 4.83 (s, 2H, NCH_2CO), 6.90 (d, 1H, Ar, J = 9.0 Hz), 7.17 (d, 1H, Ar, J = 9.6 Hz).

2-[3-Cyclohexyl-6-oxopyridazin-1(6H)-yl]acetic acid (27). A suspension of derivative **26** (0.91 mmol) in 6 N NaOH (4 mL) was stirred at 80 °C for 2 h. The mixture was then diluted with cold water and acidified with 6 N HCl. Product **27** was collected by filtration and recrystallised from ethanol. Yield = 82 %; mp = 195-197 °C (EtOH). ^{1}H NMR ($CDCl_3$) δ 1.25-1.34 (m, 1H, CH-*H* cyclohexyl), 1.37-1.46 (m, 4H, 2 x CH_2 cyclohexyl), 1.76 (m, 1H, CH-*H* cyclohexyl), 1.79-1.93 (m, 4H, 2 x CH_2 cyclohexyl), 2.50-2.60 (m, 1H, CH cyclohexyl), 4.94 (s, 2H, NCH_2CO), 6.99 (d, 1H, Ar, J = 9.5 Hz), 7.24 (d, 1H, Ar, J = 9.6 Hz).

General procedure for 3a,b. Et_3N (2.06 mmol) was added to a cooled (-5 °C) and stirred solution of intermediate **27** (0.59 mmol) in anhydrous tetrahydrofuran (3 mL). After 30 min, the mixture was allowed to warm up to 0 °C and ethyl chloroformate (0.65 mmol) was added. After 1 h, the appropriately substituted arylamine (1.18 mmol) was added. The reaction was carried out at room temperature for 12 h. The mixture was then concentrated *in vacuo*, diluted with cold water (10-15 mL) and extracted with CH_2Cl_2 (3 x 15 mL). The solvent was evaporated to afford final compounds **3a,b**, which were purified by column chromatography using cyclohexane/ethyl acetate 1:1 (for compound **3a**) and cyclohexane/ethyl acetate 1:2 (for compound **3b**) as eluents.

N-(4-Fluorophenyl)-2-[3-cyclohexyl-6-oxopyridazin-1(6H)-yl]acetamide (3a). Yield = 98 %; mp = 149-151 °C (EtOH). ^{1}H NMR ($CDCl_3$) δ 1.21-1.30 (m, 1H, CH-*H* cyclohexyl), 1.39-1.48 (m, 4H, 2 x CH_2 cyclohexyl), 1.77 (d, 1H, CH-*H* cyclohexyl, J = 12.6 Hz), 1.85-1.93 (m, 4H, 2 x CH_2 cyclohexyl), 2.56-2.63 (m, 1H, CH cyclohexyl), 4.96 (s, 2H, NCH_2CO), 6.94-7.02 (m, 3H, Ar), 7.27 (d, 1H, Ar, J = 9.7 Hz), 7.46-7.51 (m, 2H, Ar), 9.10 (exch br s, 1H, NH).

N-(1,3-Benzodioxol-5-yl)-2-[3-cyclohexyl-6-oxopyridazin-1(6H)-yl]acetamide (3b). Yield = 99%; mp = 185-187 °C (EtOH). ^{1}H NMR ($CDCl_3$) δ 1.20-1.28 (m, 1H, CH-*H* cyclohexyl), 1.30-1.48 (m, 4H, 2 x CH_2 cyclohexyl), 1.76 (d, 1H, CH-*H* cyclohexyl, J = 12.8 Hz), 1.85-1.93 (m, 4H, 2 x CH_2 cyclohexyl), 2.59-2.61 (m, 1H, CH cyclohexyl), 4.93 (s, 2H, NCH_2CO), 5.94 (s, 2H, OCH_2O), 6.72

(d, 1H, Ar, *J* = 8.4 Hz), 6.81 (dd, 1H, Ar, *J* = 6.3 Hz, *J* = 2.1 Hz), 7.00 (d, 1H, Ar, *J* = 9.5 Hz), 7.25-7.28 (m, 2H, Ar), 8.86 (exch br s, 1H, NH).

General procedure for 5a,b. A mixture of **4a**[24] or **4b**[24] (0.20 mmol), acetone (2 mL) and 6 M HCl (4 mL) was warmed in a sealed tube at 100°C for 5 h. The solvent was removed *in vacuo* and the residue was treated with cold water. The precipitate was purified by recrystallisation from ethanol to give pure **5a** and **5b** as colourless crystals or yellowish crystals, respectively.

5-Acetyl-2-benzyl-4-chloro-6-phenylpyridazin-3(2H)-one (5a). Yield = 45 %; mp = 143-146 °C (EtOH). ^{1}H NMR ($CDCl_3$) δ 2.20 (s, 3H, CH_3), 5.45 (s, 2H, CH_2), 7.30-7.60 (m, 10H, 2 x C_6H_5).

5-Acetyl-4-chloro-2-(3-cyanobenzyl) -6-phenylpyridazin-3(2H)-one (5b). Yield = 45%; mp = 141-143 °C (EtOH). ^{1}H NMR ($CDCl_3$) δ 2.20(s, 3H, CH_3), 5.45 (s, 2H, CH_2), 7.30-7.60 (m, 10H, 2 x C_6H_5).

General procedure for 6a,b. A mixture of **4a**[24] or **4b**[24] (0.09 mmol) acetone (1 mL) and 47% HBr (1 mL) was warmed in a sealed tube at 90°C for 2-3 h. After concentration *in vacuo*, ice-cold water was added and the was collected by suction. Recrystallisation from ethanol gave **6a** as colourless solid. Purification through column chromatography (eluent: toluene/ethyl acetate 8:2) afforded pure **6b** as colourless crystals.

5-Acetyl-2-benzyl-4-bromo-6-phenylpyridazin-3(2H)-one (6a). Yield = 61%; mp = 140-143 °C (EtOH). ^{1}H NMR ($CDCl_3$) δ 2.15 (s, 3H, CH_3), 5.45 (s, 2H, CH_2), 7.30-7.60 (m, 10H, 2 x C_6H_5).

5-Acetyl-4-bromo-2-(3-cyanobenzyl) -6-phenylpyridazin-3(2H)-one (6b). Yield = 85%; mp = 150-153 °C (EtOH). ^{1}H NMR ($CDCl_3$) δ 2.20 (s, 3H, CH_3), 5.45 (s, 2H, CH_2), 7.40-7.85 (m, 10H, 2 x C_6H_5).

5-Benzyl- 3-methyl-7-phenylisoxazolo[4,3-d]pyridazin-4(5H)-one (31). A mixture of compound **30**[24] (0.45 mmol), K_2CO_3 (0.90 mmol), benzyl bromide (0.70 mmol) and anhydrous DMF (1.2 mL) was warmed at 90°C for 40 min. After cooling and treatment with ice cold water, the precipitate was collected by suction. Recrystallisation from ethanol gave pure compound **31** as colourless crystals. Yield = 73%; mp = 128-130 °C (EtOH). ^{1}H NMR ($CDCl_3$) δ 2.95 (s, 3H, CH_3), 5.35 (s, 2H, CH_2), 7.30-7.60 (m, 10H, 2 x C_6H_5).

4-Acetyl-5-amino-2-benzyl-6-phenylisoxazolo-3(2H)-one (7). A suspension of intermediate **31** (0.22 mmol), 10% Pd/C (0.05 mmol) and ammonium formate (0.95 mmol) in ethanol (1 mL) was refluxed for 30 min. After cooling, methylene chloride (15 mL) was added and the precipitate was filtered off.

Evaporation *in vacuo* afforded the crude product which was recrystallised from ethanol to give pure **7** as colourless crystals. Yield = 59 %; mp = 153-155 °C (EtOH). ^{1}H NMR ($CDCl_3$) δ 2.75 (s, 3H, CH_3), 5.30 (s, 2H, CH_2), 7.25-7.60 (m, 10H, 2 x C_6H_5).

3-[(6-Methyl-3-oxo-2,3-dihydropyridazin-4-yl)methyl[benzoic acid (33). Compound **32** (1.78 mmol) and 3-cyanobenzaldehyde (3.56 mmol) were added to 6 mL of KOH in absolute EtOH (5%, w/v). The mixture was refluxed under stirring for 4 h. After cooling, the suspension was concentrated *in vacuo,* diluted with ice-cold water (10 mL) and acidified with 2 N HCl. After 1 h stirring in ice-bath, the precipitate was filtered off and purified by crystallisation from ethanol. Yield = 70 %; mp = 164-166 °C (EtOH). ^{1}H NMR ($CDCl_3$) δ 2.18 (s, 3H, 6-CH_3), 3.80 (s, 2H, CHCCH_2), 7.06 (s, 1H, pyridaz), 7.33 (d, 2H, Ar, J = 8.2 Hz), 7.80 (d, 2H, Ar, J = 8.1 Hz), 12.72 (exch br s, 1H, OH). IR (cm^{-1}): 3300 (NH), 3200 (OH), 1649 (CO), 1608 (CO).

3-{[2-(2-Ethoxy-2-oxoethyl)-6-methyl-3-oxo-2,3-dihydropyridazin-4-yl]methyl}benzoic acid (34). A mixture of compound **33** (1.56 mmol), K_2CO_3 (3.12 mmol) and ethyl bromoacetate (2.34 mmol) in anhydrous CH_3CN (6 mL) was refluxed under stirring for 2 h. The mixture was then concentrated *in vacuo* and diluted with cold water. After 1 h stirring in ice-bath, the yellow precipitate was filtered off by suction and purified by recrystallisation from ethanol. Yield = 78 %; mp = 174-176 °C (EtOH). ^{1}H NMR ($CDCl_3$) δ 1.31 (t, 3H, CH_2CH_3, J = 7.2 Hz), 2.25 (s, 3H, 3-CH_3), 3.96 (s, 2H, CHCCH_2), 4.26 (q, 2H, CH_2CH_3, J = 7.2 Hz), 4.86 (s, 2H, NCH_2CO), 6.70 (s, 1H, pyridaz), 7.34 (d, 2H, Ar, J = 7.7 Hz), 7.80 (d, 2H, Ar, J = 7.7 Hz).

3-{[2-(Carboxymethyl)-6-methyl-3-oxo-2,3-dihydropyridazin-4-yl]methyl} benzoic acid (35). A suspension of the intermediate **34** (1.22 mmol) in 6 N NaOH (5 mL) was stirred at 60 °C for 2 h. The mixture was diluted with ice-cold water (3 mL), acidified with 6 N HCl and the final product **35** was then filtered off by suction and recrystallised from ethanol. Yield = 76 %; mp = 225-227 °C (EtOH). ^{1}H NMR ($CDCl_3$) δ 2.22 (s, 3H, 6-CH_3), 3.87 (s, 2H, CHCCH_2), 4.69 (s, 2H, NCH_2CO), 7.15 (s, 1H, pyridaz), 7.39 (d, 2H, Ar, J = 8.0 Hz), 7.88 (d, 2H, Ar, J = 8.0 Hz), 13.01 (exch br s, 2H, 2 x OH).

N-(4-Bromophenyl)-3-{2-[(4-bromophenylcarbamoyl)methyl]-6-methyl-3-oxo-2,3-dihydro-pyridazin-4-ylmethyl}benzamide (8). Et_3N (3.26 mmol) was added to a cooled (-5 °C) and stirred solution of compound **35** (0.93 mmol) in anhydrous tetrahydrofuran (7 mL). After 30 min, the mixture was allowed to warm up to 0 °C and ethyl chloroformate (1.02 mmol) was added. After 1 h, 4-bromo aniline (1.86 mmol) was added. The reaction was carried out at room temperature for 12 h. The mixture was then concentrated *in vacuo*, diluted with cold water (15 mL) and extracted with CH_2Cl_2 (3 x 15 mL). After removal of the solvent, the residue was purified by column chromatography using

$CH_2Cl_2/CH_3OH/NH_4OH$ 9.5:0.5:0.05 as eluent. The pure sample of **8** was obtained from a further purification through a silica gel preparative TLC (eluent: $CH_2Cl_2/CH_3OH/$ NH_4OH 9.5:0.5:0.05). Yield = 10 %; mp = 226-228 °C (EtOH). ^1H NMR ($CDCl_3$) δ 2.31 (s, 3H, 6-CH_3), 4.00 (s, 2H, CHCCH_2), 4.93 (s, 2H, NCH_2CO), 6.83 (s, 1H, pyridaz), 7.40 (t, 6H, Ar, J = 8.4 Hz), 7.51 (d, 2H, Ar, J = 8.7 Hz), 7.57 (d, 2H, Ar, J = 8.9 Hz), 7.84 (d, 2H, Ar, J = 7.7 Hz), 8.67 (exch br s, 1H, NH). MS (ESI) calcd. For $C_{27}H_{22}Br_2N_4O_3$, 610.30. Found: *m/z* 609 $[M - H]^-$, 611.2 $[M + H]^+$.

Ethyl-2-{5-[bis(4-methoxyphenyl)amino]-3-methyl-6-oxopyridazin-1(6H)-yl}acetate (37). Et_3N (0.64 mmol) was added to a suspension of compound **36**[25] (0.57 mmol), copper acetate (0.85 mmol) and 4-methoxyphenylboronic acid (1.14 mmol) in CH_2Cl_2 (3 mL). The mixture was stirred at room temperature for 14 h and extracted with 15% aqueous ammonia (3 x 10 mL). The organic layer was washed with 10 mL of water and dried over Na_2SO_4. After removal of the solvent *in vacuo*, the residue was purified by flash column chromatography using cyclohexane/ethyl acetate 1:3 as eluent. Yield = 21 %; colourless oil. ^1H NMR ($CDCl_3$) δ 1.28 (t, 3H, CH_2CH_3, J = 7.2 Hz), 2.18 (s, 3H, 3-CH_3), 3.81 (s, 6H, 2 x OCH_3), 4.21 (q, 2H, CH_2CH_3, J = 7.2 Hz), 4.83 (s, 2H, NCH_2CO), 6.32 (s, 1H, pyridaz), 6.86 (dd, 4H, Ar, J = 3.4 Hz, J = 2.3 Hz), 6.99 (dd, 4H, Ar, J = 4.5 Hz, J = 2.3 Hz).

2-{5-[Bis-(4-methoxyphenyl)amino]-3-methyl-6-oxopyridazin-1(6H)-yl}acetic acid (38). A suspension of the intermediate **37** (0.12 mmol), 6 NaOH (10 mL) and EtOH (3 mL) was stirred at rt 12 h. After removal of the solvent under vacuum, the mixture was diluted with ice-cold water and acidified with 6 N HCl. After 1 h stirring in ice-bath, the product **38** was collected by filtration and recrystallised from ethanol. Yield = 84 %; mp = 192-193 °C (EtOH). ^1H NMR ($CDCl_3$) δ 2.20 (s, 3H, 3-CH_3), 3.82 (s, 6H, 2 x OCH_3), 4.88 (s, 2H, NCH_2CO), 6.34 (s, 1H, pyridaz), 6.86 (d, 4H, Ar, J = 8.8 Hz), 6.99 (d, 4H, Ar, J = 8.8 Hz).

N-(4-Bromophenyl)-2-{5-[bis(4-methoxyphenyl)amino]-3-methyl-6-oxopyridazin-1(6H)-yl}acetamide (9). Et_3N (0.35 mmol) was added to a cooled (-5 °C) and stirred solution of compound **38** (0.10 mmol) in anhydrous tetrahydrofuran (4 mL). After 30 min, the mixture was allowed to warm up to 0 °C and ethyl chloroformate (0.11 mmol) was added. After 1 h 4-bromo aniline (0.20 mmol) was added and the reaction was carried out at room temperature for 12 h. The mixture was then concentrated *in vacuo,* diluted with cold water (10 mL) and extracted with CH_2Cl_2 (3 x 15 mL). The solvent was evaporated to afford final compound **9**, which was purified by flash column chromatography using cyclohexane/ethyl acetate 1:1 as eluent. Yield = 55 %; mp = 244-245 °C (EtOH). ^1H NMR ($CDCl_3$) δ 2.23 (s, 3H, 3-CH_3), 3.79 (s, 6H, 2 x OCH_3), 4.81 (s, 2H, NCH_2CO), 6.39 (s, 1H,pyridaz), 6.84 (dd, 4H, Ar, J = 4.6 Hz, J = 2.2 Hz), 6.99 (dd, 4H, Ar, J = 2.2 Hz, J = 3.4 Hz), 7.25-7.38 (m, 4H, Ar), 9.00 (exch br s, 1H, NH).

General procedure for compounds 40a-g. Compounds **40a-g** were obtained using the same procedure (Knovenagel condensation) and treatment followed for the synthesis of intermediate **33**, using the appropriate (hetero)arylaldehyde and starting from compound **39**. The products were recovered by *vacuum* filtration and purified by crystallization from diethyl ether (for **40a**) or ethanol (for **40b-g**).

4-((3-Oxo-6-phenyl-2,3-dihydropyridazin-4-yl)methyl)benzamide (40a). Yield = 57 %; mp = 263-266 °C (EtOH). ^{1}H NMR (DMSO-d_6) δ 3.92 (s, 2H, CH_2-Ph), 7.30 (exch br s, 1H, CONH-*H*), 7.40-7.55 (m, 5H, Ar), 7.80-7.85 (m, 4H, Ar), 7.91 (exch br s, 1H, CONH-*H*), 7.93 (s, 1H, pyridaz.), 13.17 (exch br s, 1H, NH).

6-Phenyl-4-(4-(pyrimidin-5-yl)benzyl)pyridazin-3(2H)-one (40b). Yield = 82 %; mp = 242-244 °C (EtOH). ^{1}H NMR (DMSO-d_6) δ 3.95 (s, 2H, CH_2-Ph), 7.42-7.55 (m, 5H, Ar), 7.76 (d, 2H, Ar, *J* = 8.0 Hz), 7.84 (d, 2H, Ar, *J* = 7.2 Hz), 8.00 (s, 1H, pyridaz.), 9.13 (s, 2H, pyrimidine), 9.17 (s, 1H, pyrimidine), 13.18 (exch br s, 1H, NH).

4-(6-((3-Oxo-6-phenyl-2,3-dihydropyridazin-4-yl)methyl)pyridin-2-yl)benzamide (40c). Yield = 65 %; mp = 243-247 °C (EtOH). ^{1}H NMR (DMSO-d_6) δ 4.15 (s, 2H, -CH_2-), 7.36 (d, 1H, pyridine, J = 7.2 Hz), 7.42 (exch br s, 1H, CONH-*H*), 7.43-7.53 (m, 3H, Ar), 7.84-7.92 (m, 4H, 2H Ar + 2H pyridine), 7.95 (d, 2H, Ar, *J* = 8.4 Hz), 8.04 (exch br s, 1H, CONH-*H*), 8.05 (s, 1H, pyridaz.), 8.13 (d, 2H, Ar, *J* = 8.4 Hz), 13.21 (exch br s, 1H, NH).

4-(3-(Cyclopentyloxy)-4-methoxybenzyl)-6-phenylpyridazin-3(2H)-one (40d). Yield = 65 %; mp = 243-247 °C (EtOH). ^{1}H NMR ($CDCl_3$) δ 1.55-1.68 (m, 2H, cyclopent.), 1.80-2.00 (m, 6H, cyclopent.), 3.88 (s, 3H, OCH_3), 3.94 (s, 2H, -CH_2-Ph), 4.77-4.82 (m, 1H, cyclopent.), 6.80-6.90 (m, 3H, Ar), 7.33 (s, 1H, pyridaz.), 7.43-7.48 (m, 3H, Ar), 7.65-7.70 (m, 2H, Ar), 11.02 (exch br s, 1H, NH).

6-Phenyl-4-(3-(pyridin-2-yl)benzyl)pyridazin-3(2H)-one (40e). Yield = 51 %; mp = 199-200 °C (EtOH). ^{1}H NMR (DMSO-d_6) δ 3.94 (s, 2H, CH_2-Ph), 7.33 (dd, 1H, pyridine, *J* = 4.8 Hz and *J* = 7.2 Hz), 7.41-7.51 (m, 5H, Ar), 7.83 (d, 2H, Ar, *J* = 8.0 Hz), 7.87 (d, 1H, pyridine, *J* = 7.6 Hz), 7.93 (d, 1H, pyridine, *J* = 8.0 Hz), 7.96 (s, 1H, pyridaz.), 8.03 (d, 2H, Ar, *J* = 8.0 Hz), 8.65 (m, 1H, pyridine), 13.18 (exch br s, 1H, NH).

4-(3-(Cyclopentyloxy)-4-methoxybenzyl)-6-methylpyridazin-3(2H)-one (40f). Yield = 17 %; mp = 155-157 °C (EtOH). ^{1}H NMR ($CDCl_3$) δ 1.85-2.00 (m, 8H, cyclopent.), 2.24 (s, 3H, CH_3), 3.84 (s,

2H, -CH_2-Ph), 3.87 (s, 3H, OCH_3), 4.75-4.80 (m, 1H, cyclopent.), 6.69 (s, 1H, pyridaz.), 6.75-6.80 (m, 2H, Ar), 6.85-6.90 (m, 1H, Ar), 11.20 (exch br s, 1H, NH).

6-Methyl-4-(3-(pyrimidin-5-yl)benzyl)pyridazin-3(2H)-one (40g). Yield = 54 %; mp = 244-246 °C (EtOH). ^{1}H NMR (DMSO-d_6) δ 2.19 (s, 3H, CH_3), 3.83 (s, 2H, CH_2-Ph), 7.13 (s, 1H, pyridaz.), 7.45 (d, 2H, Ar, *J* = 8.4 Hz), 7.76 (d, 2H, Ar, *J* = 8.4 Hz), 9.14 (s, 2H, pyrimidine), 9.18 (s, 1H, pyrimidine), 12.73 (exch br s, 1H, NH).

General procedure for compounds 10a-g. A mixture of intermediates **40a-g** (0.58 mmol), K_2CO_3 (1.17 mmol), ethyl bromide (0.88 mmol) and anhydrous CH_3CN (8 mL) was stirred at reflux for 4-6 hours about. After cooling, the solvent was evaporated and ice-cold water was added. The formed precipitate was recovered by *vacuum* filtration and the final compounds **10a-g** were purified by crystallization from ethanol.

4-((2-Ethyl-3-oxo-6-phenyl-2,3-dihydropyridazin-4-yl)methyl)benzamide (10a). Yield = 33 %; mp = 215-218°C (EtOH). ^{1}H NMR (DMSO-d_6) δ 1.32 (t, 3H, $CH_2\mathit{CH_3}$, *J* = 7.2 Hz), 3.95 (s, 2H, CH_2-Ph), 4.18 (q, 2H, $\mathit{CH_2}CH_3$, *J* = 7.2 Hz), 7.30 (exch br s, 1H, CONH-*H*), 7.43 (d, 2H, Ar, *J* = 7.6 Hz), 7.45-7.50 (m, 3H, Ar), 7.81 (d, 2H, Ar, *J* = 7.2 Hz), 7.84 (d, 2H, Ar, *J* = 7.6 Hz), 7.92 (s, 2H, 1H CONH-*H* + 1H pyridaz.).

2-Ethyl-6-phenyl-4-(4-(pyrimidin-5-yl)benzyl)pyridazin-3(2H)-one (10b). Yield = 38 %; mp = 150-151 °C (EtOH). ^{1}H NMR (DMSO-d_6) δ 1.33 (t, 3H, $CH_2\mathit{CH_3}$, *J* = 7.2 Hz), 3.98 (s, 2H, CH_2-Ph), 4.18 (q, 2H, $\mathit{CH_2}CH_3$, *J* = 7.2 Hz), 7.45-7.55 (m, 5H, Ar), 7.76 (d, 2H, Ar, *J* = 8.0 Hz), 7.86 (d, 2H, Ar, *J* = 7.2 Hz), 8.00 (s, 1H, pyridaz.), 9.13 (s, 2H, pyrimidine), 9.17 (s, 1H, pyrimidine).

4-(6-((2-Ethyl-3-oxo-6-phenyl-2,3-dihydropyridazin-4-yl)methyl)pyridin-2-yl)benzamide (10c). Yield = 49 %; mp = 207-208 °C (EtOH). ^{1}H NMR ($CDCl_3$) δ 1.47 (t, 3H, $CH_2\mathit{CH_3}$, *J* = 7.2 Hz), 4.28-4.38 (m, 4H, 2H -CH_2-pyridine + 2H N-$\mathit{CH_2}CH_3$), 5.78 (exch br s, 1H, CONH-*H*), 6.25 (exch br s, 1H, CONH-*H*), 7.39-7.51 (m, 4H, 3H Ar + 1H pyridaz.), 7.69 (d, 1H, pyridine, *J* = 7.6 Hz), 7.75-7.85 (m, 4H, 2H Ar + 2H pyridine), 7.92 (d, 2H, Ar, *J* = 8.4 Hz), 8.10 (d, 2H, Ar, *J* = 8.4 Hz).

4-(3-(Cyclopentyloxy)-4-methoxybenzyl)-2-ethyl-6-phenylpyridazin-3(2H)-one (10d). Yield = 20 %; oil. ^{1}H NMR ($CDCl_3$) δ 1.48 (t, 3H, $CH_2\mathit{CH_3}$, *J* = 7.2 Hz), 1.53-1.68 (m, 2H, cyclopent.), 1.78-2.02 (m, 6H, cyclopent.), 3.88 (s, 3H, OCH_3), 3.92 (s, 2H, -CH_2-Ph), 4.36 (q, 2H, $\mathit{CH_2}CH_3$, *J* = 7.2 Hz), 4.75-4.80 (m, 1H, cyclopent.), 6.78-6.90 (m, 3H, Ar), 7.22 (s, 1H, pyridaz.), 7.40-7.45 (m, 3H, Ar), 7.69 (d, 2H, Ar, *J* = 8.0 Hz).

2-Ethyl-6-phenyl-4-(3-(pyridin-2-yl)benzyl)pyridazin-3(2H)-one (10e). Yield = 68 %; mp = 134-136 °C (EtOH). ^{1}H NMR (DMSO-d$_6$) δ 1.33 (t, 3H, CH$_2$*CH$_3$*, *J* = 6.8 Hz), 3.97 (s, 2H, CH$_2$-Ph), 4.19 (q, 2H, *CH$_2$*CH$_3$, *J* = 6.8 Hz), 7.34 (m, 1H, pyridine), 7.41-7.57 (m, 5H, Ar), 7.86 (d, 3H, Ar, *J* = 7.2 Hz), 7.92 (s, 1H, pyridine), 7.95 (s, 1H, pyridaz.), 8.03 (d, 2H, Ar, *J* = 7.6 Hz), 8.65 (m, 1H, pyridine).

4-(3-(Cyclopentyloxy)-4-methoxybenzyl)-2-ethyl-6-methylpyridazin-3(2H)-one (10f). Yield = 66 %; oil. ^{1}H NMR (CDCl$_3$) δ 1.38 (t, 3H, CH$_2$*CH$_3$*, *J* = 7.2 Hz), 1.55-1.65 (m, 2H, cyclopent.), 1.80-1.98 (m, 6H, cyclopent.), 2.23 (s, 3H, CH$_3$), 3.82 (s, 2H, -CH$_2$-Ph), 3.86 (s, 3H, OCH$_3$), 4.21 (q, 2H, *CH$_2$*CH$_3$, *J* = 7.2 Hz), 4.75-4.80 (m, 1H, cyclopent.), 6.61 (s, 1H, pyridaz.), 6.75-6.80 (m, 2H, Ar), 6.85 (d, 1H, Ar, *J* = 8.0 Hz).

2-Ethyl-6-methyl-4-(3-(pyrimidin-5-yl)benzyl)pyridazin-3(2H)-one (10g). Yield = 44 %; mp = 148-150 °C (EtOH). ^{1}H NMR (CDCl$_3$) δ 1.38 (t, 3H, CH$_2$*CH$_3$*, *J* = 7.2 Hz), 2.27 (s, 3H, CH$_3$), 3.96 (s, 2H, CH$_2$-Ph), 4.20 (q, 2H, *CH$_2$*CH$_3$, *J* = 7.2 Hz), 6.73 (s, 1H, pyridaz.), 7.43 (d, 2H, Ar, *J* = 8.4 Hz), 7.57 (d, 2H, Ar, *J* = 8.4 Hz), 8.96 (s, 2H, pyrimidine), 9.21 (s, 1H, pyrimidine).

General procedure for compounds 11 and 12. A suspension of intermediate **10a** or **10c** (0.54 mmol) in 5 mL of POCl$_3$ was stirred at 60-70 °C for 2h about. After cooling, ice-cold water (20 mL) was slowly added, and the precipitate was filtered under *vacuum* and washed with abundant cold-water to obtain the desired compounds **11** and **12**, which were recrystallized from ethanol.

4-((2-Ethyl-3-oxo-6-phenyl-2,3-dihydropyridazin-4-yl)methyl)benzonitrile (11). Yield = 59 %; mp = 129-130 °C (EtOH). ^{1}H NMR (CDCl$_3$) δ 1.47 (t, 3H, CH$_2$*CH$_3$*, *J* = 7.2 Hz), 4.05 (s, 2H, CH$_2$-Ph), 4.33 (q, 2H, *CH$_2$*CH$_3$, *J* = 7.2 Hz), 7.34 (s, 1H, pyridaz.), 7.40-7.50 (m, 5H, Ar), 7.66 (d, 2H, Ar, *J* = 8.4 Hz), 7.72 (d, 2H, Ar, *J* = 6.8 Hz). IR ν (cm^{-1}): 2221 (CN), 1655 (CO).

4-(6-((2-Ethyl-3-oxo-6-phenyl-2,3-dihydropyridazin-4-yl)methyl)pyridin-2-yl)benzonitrile (12). Yield = 52 %; mp = 146-148 °C (EtOH). ^{1}H NMR (CDCl$_3$) δ 1.47 (t, 3H, CH$_2$*CH$_3$*, *J* = 7.2 Hz), 4.26 (s, 2H, -CH$_2$-pyridine), 4.34 (q, 2H, *CH$_2$*CH$_3$, *J* = 7.2 Hz), 7.40-7.50 (m, 4H, 3H Ar + 1H pyridaz.), 7.65-7.70 (m, 2H, pyridine), 7.76-7.82 (m, 5H, 4H Ar + 1H pyridine), 8.13 (d, 2H, Ar, *J* = 8.4 Hz). IR ν (cm^{-1}): 2223 (CN), 1650 (CO).

General procedure for compounds 13a-c. 0.35 mmol of 4,6-diphenyl-isoxazol[3,4-d]pyridazin-7(6H)-one **41**[27] was dissolved in 0.5 mL of 1,4-dioxane in a sealed tube. 1.22 mmol of suitable amine or alcohol was added and the reaction was stirred at 90 °C for 2-3 h (for compound **13c** some drops of Et$_3$N were added). After cooling, the solvent was evaporated and ice-cold water was added. The

formed precipitate was filtered off to obtaine the desired compounds **13a-c** which were recrystallized from ethanol.

5-Amino-6-oxo-1,3-diphenyl-N-propyl-1,6-dihydropyridazine-4-carboxamide (13a). Yield = 58 %; mp = 162-164 °C (EtOH). ^{1}H NMR (DMSO-d$_6$) δ 0.62 (t, 3H, $CH_2CH_2\mathit{CH_3}$, J = 7.4 Hz), 1.21 (sest, 2H, $CH_2\mathit{CH_2}CH_3$, J = 7.2 Hz), 3.00 (q, 2H, $\mathit{CH_2}CH_2CH_3$, J = 6.8 Hz), 6.62 (exch br s, 2H, NH_2), 7.38-7.44 (m, 4H, Ar), 7.50-7.55 (m, 4H, Ar), 7.62 (d, 2H, Ar, J = 7.6 Hz), 8.12 (t, 1H, NH, J = 5.6 Hz).

4-Amino-2,6-diphenyl-5-(piperidine-1-carbonyl)pyridazin-3(2H)-one (13b). Yield = 31 %; mp = 199-201 °C (EtOH). ^{1}H NMR ($CDCl_3$) δ 1.15-1.20 (m, 1H, piperidine), 1.25-1.30 (m, 1H, piperidine), 1.30-1.40 (m, 2H, piperidine), 1.40-1.55 (m, 2H, piperidine), 2.75-2.85 (m, 1H, piperidine), 3.05-32.15 (m, 1H, piperidine), 3.50-3.55 (m, 2H, piperidine), 5.69 (exch br s, 2H, NH_2), 7.38-7.45 (m, 4H, Ar), 7.50 (t, 2H, Ar, J = 7.8 Hz), 7.60-7.70 (m, 2H, Ar), 7.74 (d, 2H, Ar, J = 8.0 Hz).

Cyclohexyl 5-amino-6-oxo-1,3-diphenyl-1,6-dihydropyridazine-4-carboxylate (13c). Yield = 30 %; mp = 129-131 °C (EtOH). ^{1}H NMR ($CDCl_3$) δ 0.90-1.15 (m, 3H, cyclohexane), 1.15-1.30 (m, 3H,cyclohexane), 1.40-1.50 (m, 3H, cyclohexane), 1.55-1.60 (m, 2H, cyclohexane), 4.71-4.76 (m, 1H, cyclohexane), 7.36-7.42 (m, 6H, Ar), 7.43-7.50 (m, 2H, Ar), 7.69-7.74 (m, 2H, Ar).

4-Cyclohexyl-3-methyl-6-propylisoxazolo[3,4-d]pyridazin-7(6H)-one (43e). A mixture of compound **42**[31] (0.45 mmol), K_2CO_3 (0.90 mmol), bromopropane (2.25 mmol, added twice) and anhydrous acetone (2 mL) was warmed at 90 °C for 5 h. After cooling, the solvent was evaporated and ice cold-water was added. The suspension was extracted with ethyl acetate (3 x 10 mL), dried on sodium sulfate and evaporated to obtain the desired compound which was purified by column chromatography to remove the excess of bromopropane using cyclohexane/ethyl acetate 3:1 as eluent. Yield = 70%; mp = 160-162 °C (EtOH). ^{1}H NMR ($CDCl_3$) δ 0.95 (t, 3H, $CH_2CH_2\mathit{CH_3}$, J = 7.2 Hz), 1.35-1.45 (m, 6H, cC_6H_{11}), 1.80 (sest, 2H, $CH_2\mathit{CH_2}CH_3$, J = 7.2 Hz), 1.88-1.99 (m, 4H, cC_6H_{11}), 2.83 (s, 3H, 3-CH_3), 4.06 (t, 2H, $CH_2CH_2CH_3$, J = 7.2 Hz).

General procedure for compounds 44b-m. To a solution of intermediates **43b-g**[27-29] (0.60 mmol) in 1.25 mL of anhydrous MeOH, 1.5 mmol of appropriate (hetero)arylaldehyde and a solution of MeONa (0.65 mmol of Na^0 in 1 mL of MeOH) were added. The mixture was stirred at reflux for 2-20 min about. After cooling, the precipitate was filtered off to obtain the styril derivatives **44b-m**.

(E)-4-Phenyl-6-propyl-3-styrylisoxazolo[3,4-d]pyridazin-7(6H)-one (44b). Yield = 57 %; mp = 154-156 °C (EtOH). ^{1}H NMR ($CDCl_3$) δ 1.02 (t, 3H, $CH_2CH_2\mathit{CH_3}$, J = 7.2 Hz), 1.91 (sest, 2H,

CH_2*CH_2*CH_3, *J* = 7.2 Hz), 4.22 (t, 2H, *CH_2*CH_2CH_3, *J* = 7.2 Hz), 6.80 (d, 1H, CH=*CH*, *J* = 16.4 Hz), 7.32-7.39 (m, 5H, Ar), 7.59-7.65 (m, 5H, Ar), 8.30 (d, 1H, *CH*=CH, *J* = 16.4 Hz).

(E)-6-Butyl-4-phenyl-3-styrylisoxazolo[3,4-d]pyridazin-7(6H)-one (44c). Yield = 55%; mp = 90-92 °C (EtOH). ^1H NMR ($CDCl_3$) δ 0.99 (t, 3H, $CH_2CH_2CH_2$*CH_3*, *J* = 7.2 Hz), 1.45 (sest, 2H, CH_2CH_2*CH_2*CH_3, *J* = 7.2 Hz), 1.86 (quin, 2H, CH_2*CH_2*CH_2CH_3, *J* = 7.2 Hz), 4.24 (t, 2H, *CH_2*CH_2 CH_2CH_3, *J* = 7.2 Hz), 6.80 (d, 1H, CH=*CH*, *J* = 16.4 Hz), 7.30-7.40 (m, 5H, Ar), 7.55-7.70 (m, 5H, Ar), 7.92 (d, 1H, *CH*=CH, *J* = 16.4 Hz).

(E)-6-Isopropyl-4-phenyl-3-styrylisoxazolo[3,4-d]pyridazin-7(6H)-one (44d). Yield = 51 %; mp = 187-189 °C (EtOH). ^1H NMR ($CDCl_3$) δ 1.38 (d, 6H, CH(*CH_3)$_2$*, *J* = 6.4 Hz), 5.40 (m, 1H, *CH*$(CH_3)_2$), 6.85 (d, 1H, CH=*CH*, *J* = 16.4 Hz), 7.30-7.45 (m, 5H, Ar), 7.50-7.65 (m, 7H, 5H Ar + 1H -*CH*=CH).

(E)-4-Cyclohexyl-6-propyl-3-styrylisoxazolo[3,4-d]pyridazin-7(6H)-one (44e). Yield = 60%; mp = 156-158 °C (EtOH). ^1H NMR ($CDCl_3$) δ 0.98 (t, 3H, CH_2CH_2*CH_3*, *J* = 7.2 Hz), 1.35-1.55 (m, 6H, cC_6H_{11}), 1.80 (sest, 2H, CH_2*CH_2*CH_3, *J* = 7.2 Hz), 1.95-2.05 (m, 4H, cC_6H_{11}), 4.12 (t, 2H, $CH_2CH_2CH_3$, *J* = 7.2 Hz), 7.18 (d, 1H, CH=*CH*, *J* = 16.4 Hz), 7.45-7.50 (m, 3H, Ar), 7.60-7.65 (m, 2H, Ar), 7.80 (d, 1H, CH=*CH*, *J* = 16 Hz).

(E)-4,6-Diphenyl-3-styrylisoxazolo[3,4-d]pyridazin-7(6H)-one (44f). Yield = 58 %; mp = 224-225 °C (EtOH). ^1H NMR ($CDCl_3$) δ 6.95 (d, 1H, CH=*CH*, *J* = 16.4 Hz), 7.35-7.70 (m, 13H, Ar), 7.81-7.86 (m, 2H, Ar), 8.40 (d, 1H, *CH*=CH, *J* = 16.4 Hz).

(E)-6-Ethyl-3-(2-(naphthalen-2-yl)vinyl)-4-phenylisoxazolo[3,4-d]pyridazin-7(6H)-one (44g). Yield = 58 %; mp = 198-200 °C (EtOH). ^1H NMR ($CDCl_3$) δ 1.48 (t, 3H, CH_2*CH_3*, *J* = 7.2 Hz), 4.34 (q, 2H, *CH_2*CH_3, *J* = 7.2 Hz), 6.92 (d, 1H, CH=*CH*, *J* = 16.4 Hz), 7.45-7.70 (m, 9H, Ar), 7.91 (t, 2H, Ar, *J* = 6.8 Hz), 8.20 (d, 1H, Ar, *J* = 8.8 Hz), 8.51 (d, 1H, *CH*=CH, *J* = 16.4 Hz).

(E)-4-Phenyl-6-propyl-3-(2-(thiophen-2-yl)vinyl)isoxazolo[3,4-d]pyridazin-7(6H)-one (44h). Yield = 74 %; mp = 145-147 °C (EtOH). ^1H NMR ($CDCl_3$) δ 1.03 (t, 3H, CH_2CH_2*CH_3*, *J* = 7.2 Hz), 1.94 (sest, 2H, CH_2*CH_2*CH_3, *J* = 7.4 Hz), 4.25 (t, 2H, *CH_2*CH_2CH_3, *J* = 7.2 Hz), 6.05 (d, 1H, thiophene, *J* = 16 Hz), 6.97 (d, 1H, CH=*CH*, *J* = 16.4 Hz), 7.20 (d, 1H, thiophene, *J* = 16 Hz), 7.45 (m, 1H, thiophene), 7.50-7.65 (m, 6H, 5H Ar + 1H -*CH*=CH-).

(E)-3-(2-(Naphthalen-2-yl)vinyl)-4-phenyl-6-propylisoxazolo[3,4-d]pyridazin-7(6H)-one (44i). Yield = 42 %; mp = 182-185 °C (EtOH). ^1H NMR ($CDCl_3$) δ 1.05 (t, 3H, CH_2CH_2*CH_3*, *J* = 7.4 Hz), 1.92 (sest, 2H, CH_2*CH_2*CH_3, *J* = 7.4 Hz), 4.25 (t, 2H, *CH_2*CH_2CH_3, *J* = 7.4 Hz), 6.93 (d, 1H, CH=*CH*,

J = 16 Hz), 7.45-7.70 (m, 9H, Ar), 7.88-7.93 (m, 2H, Ar), 8.20 (d, 1H, Ar, *J* = 8.4 Hz), 8.50 (d, 1H, *CH*=CH, *J* = 16 Hz).

(E)-4-Phenyl-6-propyl-3-(2-(pyridin-4-yl)vinyl)isoxazolo[3,4-d]pyridazin-7(6H)-one (44l). Yield = 45 %; mp = 87-89 °C (EtOH). ^{1}H NMR (CDCl$_3$) δ 1.00 (t, 3H, CH$_2$CH$_2$*CH$_3$*, *J* = 7.2 Hz), 1.89 (sest, 2H, CH$_2$*CH$_2$*CH$_3$, *J* = 7.4 Hz), 4.16 (t, 2H, *CH$_2$*CH$_2$CH$_3$, *J* = 7.2 Hz), 7.19 (d, 1H, CH=*CH*, *J* = 16.4 Hz), 7.30-7.70 (m, 9H, 8H Ar + 1H -*CH*=CH-), 8.73 (d, 1H, Ar, *J* = 7.2 Hz).

(E)-4-(4-Fluorophenyl)-6-methyl-3-styrylisoxazolo[3,4-d]pyridazin-7(6H)-one (44m). Yield = 52 %; mp = 216-218 °C (EtOH). ^{1}H NMR (CDCl$_3$) δ 3.86 (s, 3H, N-*CH$_3$*), 6.78 (d, 1H, CH=*CH*, *J* = 16.4 Hz), 7.31 (d, 2H, Ar, *J* = 8.4 Hz), 7.37-7.42 (m, 5H, Ar), 7.62-7.69 (m, 3H, 2H Ar + 1H -*CH*=CH-).

General procedure for compounds 14a-c. To a solution of styryl derivatives **44a**[31],**44b** or **44m** (0.34 mmol) in CH$_3$CN, 0.45 mmol of molybdenumhexacarbonyl and 3-4 drops of H$_2$O were added. The mixture reaction was warmed at 80 °C for 3 h. After cooling, the solvent was removed *in vacuo*, the residue was recovered with ethyl acetate and the organic phase was washed with a mixture of H$_2$O/NH$_4$OH 1:1 (3 x 10 mL). After evaporation of the solvent, the final compounds **14a-c** were purified by column chromatography using cyclohexane/ethyl acetate 3:1 as eluent.

4-Amino-5-cinnamoyl-2-methyl-6-phenylpyridazin-3(2H)-one (14a). Yield = 65 %; mp = 135-137 °C (EtOH). ^{1}H NMR (CDCl$_3$) δ 3.90 (s, 3H, N-*CH$_3$*), 6.27 (d, 1H, CH=*CH*, *J* = 15.6 Hz), 7.03 (d, 2H, Ar, *J* = 7.6 Hz), 7.23-7.32 (m, 3H, Ar), 7.40-7.50 (m, 4H, 3H Ar + 1H -*CH*=CH-), 7.54 (d, 2H, Ar, *J* = 7.6 Hz).

4-Amino-5-cinnamoyl-6-phenyl-2-propylpyridazin-3(2H)-one (14b). Yield = 66 %; mp = 122-124 °C (EtOH). ^{1}H NMR (CDCl$_3$) δ 1.03 (t, 3H, CH$_2$CH$_2$*CH$_3$*, *J* = 7.2 Hz), 1.93 (sest, 2H, CH$_2$*CH$_2$*CH$_3$, *J* = 7.2 Hz), 4.21 (t, 2H, *CH$_2$*CH$_2$CH$_3$, *J* = 7.2 Hz), 6.26 (d, 1H, CH=*CH*, *J* = 15.6 Hz), 7.02 (d, 2H, Ar, *J* = 7.2 Hz), 7.20-7.30 (m, 3H, Ar), 7.38-7.47 (m, 4H, 3H Ar +1H -*CH*=CH-), 7.53 (d, 2H, Ar, *J* = 7.6 Hz).

4-Amino-5-cinnamoyl-6-(4-fluorophenyl)-2-methylpyridazin-3(2H)-one (14c). Yield = 58 %; mp = 152-154 °C (EtOH). ^{1}H NMR (CDCl$_3$) δ 3.87 (s, 3H, N-*CH$_3$*), 6.26 (d, 1H, CH=*CH*, *J* = 15.6 Hz), 6.86 (d, 2H, Ar, *J* = 6.8 Hz), 7.07-7.20 (m, 3H, Ar), 7.25-7.35 (m, 4H, Ar), 7.49 (d, 1H, *CH*=CH, *J* = 15.6 Hz).

General procedure for compounds 15a-l. A suspension of intermediates **14a,b** or **44c-l** (0.15 mmol), 10% Pd/C (0.05 mmol) and ammonium formate (0.4 mmol) in absolute ethanol (1.5 mL) was

refluxed for 2 h. After cooling, the ethanol was evaporated and methylene chloride (15 mL) was added. The precipitate was removed by filtration and the solvent was recovered and evaporated to afford the desired compounds which were purified by crystallization from ethanol.

4-Amino-2-methyl-6-phenyl-5-(3-phenylpropanoyl)pyridazin-3(2H)-one (15a). Yield = 60 %; mp = 121-123 °C (EtOH). ^{1}H NMR ($CDCl_3$) δ 2.32 (t, 2H, CO-CH_2*CH_2*-Ph, *J* = 7.6 Hz), 2.70 (t, 2H, CO-*CH_2*CH_2-Ph, *J* = 7.6 Hz), 3.82 (s, 3H, N-*CH_3*), 6.82 (d, 2H, Ar, *J* = 8.0 Hz), 7.09-7.18 (m, 3H, Ar), 7.43-7.48 (m, 5H, Ar).

4-Amino-6-phenyl-5-(3-phenylpropanoyl)-2-propylpyridazin-3(2H)-one (15b). Yield = 75 %; mp = 103-105 °C (EtOH). ^{1}H NMR ($CDCl_3$) δ 1.00 (t, 3H, CH_2CH_2*CH_3*, *J* = 7.0 Hz), 1.88 (sest, 2H, CH_2*CH_2*CH_3, *J* = 7.0 Hz), 2.31 (t, 2H, CO-CH_2*CH_2*-Ph, *J* = 7.2 Hz), 2.70 (t, 2H, CO-*CH_2*CH_2-Ph, *J* = 7.4 Hz), 4.15 (t, 2H, *CH_2*CH_2CH_3, *J* = 7.0 Hz), 6.82 (d, 2H, Ar, *J* = 7.2 Hz), 7.09-7.17 (m, 3H, Ar), 7.42-7.50 (m, 5H, Ar).

4-Amino-2-butyl-6-phenyl-5-(3-phenylpropanoyl)pyridazin-3(2H)-one (15c). Yield = 58 %; mp = 80-82 °C (EtOH). ^{1}H NMR ($CDCl_3$) δ 0.99 (t, 3H, $CH_2CH_2CH_2$*CH_3*, *J* = 7.2 Hz), 1.43 (sest, 2H, CH_2CH_2*CH_2*CH_3, *J* = 7.6 Hz), 1.85 (quin, 2H, CH_2*CH_2*CH_2CH_3, *J* = 7.6 Hz), 2.33 (t, 2H, CO-CH_2*CH_2*-Ph, *J* = 7.2 Hz), 2.71 (t, 2H, CO-*CH_2*CH_2-Ph, *J* = 7.6 Hz), 4.20 (t, 2H, *CH_2*$CH_2CH_2CH_3$, *J* = 7.4 Hz), 6.83 (d, 2H, Ar, *J* = 6.8 Hz), 7.10-7.20 (m, 3H, Ar), 7.44-7.50 (m, 5H, Ar).

4-Amino-2-isopropyl-6-phenyl-5-(3-phenylpropanoyl)pyridazin-3(2H)-one (15d). Yield = 55 %; mp = 90-93 °C (EtOH). ^{1}H NMR ($CDCl_3$) δ 1.38 (d, 6H, CH(*CH_3)$_2$*, *J* = 6.4 Hz), 2.33 (t, 2H, CO-CH_2*CH_2*-Ph, *J* = 7.2 Hz), 2.69 (t, 2H, CO-*CH_2*CH_2-Ph, *J* = 7.2 Hz), 5.20-5.30 (m, 1H, *CH*(CH_3)$_2$), 6.80 (d, 2H, Ar, J = 6.8 Hz), 7.10-7.15 (m, 2H, Ar), 7.20-7.25 (m, 1H, Ar), 7.40-7.50 (m, 5H, Ar).

4-Amino-6-cyclohexyl-5-(3-phenylpropanoyl)-2-propylpyridazin-3(2H)-one (15e). Yield = 60%; mp = 114-116 °C (EtOH). ^{1}H NMR ($CDCl_3$) δ 0.96 (t, 3H, CH_2CH_2*CH_3*, *J* = 7.6 Hz), 1.25-1.35 (m, 2H, cC_6H_{11}), 1.55-1.60 (m, 2H, cC_6H_{11}), 1.76-1.90 (m, 8H, 2H CH_2*CH_2*CH_3 + 6H cC_6H_{11}), 3.05-3.10 (m, 4H, CO-*CH_2CH_2*-Ph), 4.07 (t, 2H, *CH_2*CH_2CH_3, *J* = 7.2 Hz), 7.20-7.25 (m, 3H, Ar), 7.30-7.35 (m, 2H, Ar).

4-Amino-2,6-diphenyl-5-(3-phenylpropanoyl)pyridazin-3(2H)-one (15f). Yield = 52 %; oil. ^{1}H NMR ($CDCl_3$) δ 2.41 (t, 2H, CO-CH_2*CH_2*-Ph, *J* = 8.0 Hz), 2.76 (t, 2H, CO-*CH_2*CH_2-Ph, *J* = 8.0 Hz), 6.88 (d, 2H, Ar, *J* = 7.2 Hz), 7.15-7.20 (m, 1H, Ar), 7.40-7.60 (m, 10H, Ar), 7.71 (d, 2H, Ar, *J* = 7.2 Hz).

4-Amino-2-ethyl-5-(3-(naphthalen-1-yl)propanoyl)-6-phenylpyridazin-3(2H)-one (15g). Yield = 55 %; oil. ^{1}H NMR ($CDCl_3$) δ 1.40 (t, 3H, CH_2*CH_3*, *J* = 7.2 Hz), 2.50 (t, 2H, CO-CH_2*CH_2*-Ph, *J* = 8.0 Hz), 3.19 (t, 2H, CO-*CH_2*CH_2-Ph, *J* = 8.0 Hz), 4.22 (q, 2H, *CH_2*CH_3, *J* = 7.2 Hz), 6.97 (d, 1H, Ar, *J* = 6.8 Hz), 7.26 (d, 1H, Ar, *J* = 8.0 Hz), 7.35-7.50 (m, 7H, Ar), 7.55-7.65 (m, 2H, Ar), 7.80 (d, 1H, Ar, *J* = 8.0 Hz).

4-Amino-6-phenyl-2-propyl-5-(3-(thiophen-2-yl)propanoyl)pyridazin-3(2H)-one (15h). Yield = 50 %; mp = 103-105 °C (EtOH). ^{1}H NMR ($CDCl_3$) δ 1.01 (t, 3H, CH_2CH_2*CH_3,* *J* = 7.2 Hz), 1.88-1.93 (m, 2H, CH_2*CH_2*CH_3), 2.36 (t, 2H, CO-CH_2*CH_2*-Ph, *J* = 7.2 Hz), 2.95 (t, 2H, CO-*CH_2*CH_2-Ph, *J* = 7.2 Hz), 4.18 (m, 2H, *CH_2*CH_2CH_3), 6.49 (ds, 1H, thiophene, *J* = 3.2 Hz), 6.80 (dd, 1H, thiophene, *J* = 5.2 Hz and *J* = 3.2 Hz), 7.03 (d, 1H, thiophene, *J* = 4.8 Hz), 7.40-7.50 (m, 5H, Ar).

4-Amino-5-(3-(naphthalen-1-yl)propanoyl)-6-phenyl-2-propylpyridazin-3(2H)-one (15i). Yield = 80 %; oil. ^{1}H NMR ($CDCl_3$) δ 0.99 (t, 3H, CH_2CH_2*CH_3*, *J* = 7.4 Hz), 1.85 (sest, 2H, CH_2*CH_2*CH_3, *J* = 7.4 Hz), 2.50 (t, 2H, CO-CH_2*CH_2*-Ph, *J* = 7.8 Hz), 3.19 (t, 2H, CO-*CH_2*CH_2-Ph, *J* = 7.8 Hz), 4.14 (q, 2H, *CH_2*CH_2CH_3, *J* = 7.2 Hz), 6.97 (d, 1H, Ar, *J* = 7.2 Hz), 7.27 (t, 1H, Ar, *J* = 7.4 Hz), 7.40-7.50 (m, 7H, Ar), 7.59 (d, 1H, Ar, *J* = 8.0 Hz), 7.65 (d, 1H, Ar, *J* = 8.4 Hz), 7.81 (d, 1H, Ar, *J* = 8.0 Hz).

4-Amino-6-phenyl-2-propyl-5-(3-(pyridin-4-yl)propanoyl)pyridazin-3(2H)-one (15l). Yield = 50 %; oil. ^{1}H NMR ($CDCl_3$) δ 1.00 (t, 3H, CH_2CH_2*CH_3,* *J* = 7.4 Hz), 1.89 (sest, 2H, CH_2*CH_2*CH_3, *J* = 7.2 Hz), 2.39 (t, 2H, CO-CH_2*CH_2*-Ph, *J* = 7.2 Hz), 2.89 (t, 2H, CO-*CH_2*CH_2-Ph, *J* = 7.2 Hz), 4.16 (t, 2H, *CH_2*CH_2CH_3, *J* = 7.6 Hz), 7.21 (d, 2H, pyridine, *J* = 5.6 Hz), 7.41-7.51 (m, 5H, Ar), 8.53 (d, 2H, pyridine, *J* = 5.2 Hz).

4-(4-Fluorophenyl)-3-methyl-6-phenylisoxazolo[3,4-d]pyridazin-7(6H)-one (46b). A suspension of intermediate **45**[32] (2.89 mmol), phenyl hydrazine (5.8 mmol) and an excess of PPA (7 g) in 2.5 mL of absolute ethanol was heated at 80-90 °C for 1 h and 30 min. After cooling, the ethanol was evaporated and ice-cold water (15 mL) was added. The precipitate was recovered by filtration to obtain a yellow solid which resulted to be pure at the TLC. Yield = 60 %; mp = 175-178 °C (EtOH). ^{1}H NMR ($CDCl_3$) δ 2.62 (s, 3H, CH_3), 7.22-7.28 (m, 3H, Ar), 7.39-7.45 (m, 2H Ar), 7.49-7.60 (m, 4H, Ar).

5-Acetyl-4-amino-6-(4-fluorophenyl)-2-phenylpyridazin-3(2H)-one (47b). Compound **47b** was obtained following the same procedure used for the synthesis of compounds **15a-l**. After cooling, the precipitate was removed by gravity filtration and the organic phase was recovered and evaporated to obtain a white-yellow solid which resulted tob e pure at the TLC. Yield = 76 %; mp = 160-163 °C

(EtOH). ^{1}H NMR ($CDCl_3$) δ 1.88 (s, 3H, CH_3), 7.17 (t, 2H, Ar, *J* = 7.6 Hz), 7.41 (t, 1H, Ar, *J* = 8.0 Hz), 7.48-7.53 (m, 4H, Ar), 7.72 (d, 2H, Ar, *J* = 7.6 Hz). IR ν (cm^{-1}): 3398-3283 (NH_2), 1646 (CO), 1595 (CO).

General procedure for compounds 16a,b. In a sealed tube, 0.62 mmol of intermediates **47a,b** (**47a**[27]) and 17.68 mmol of HBr 48% were heated at 130 °C for 2 h. After cooling, the mixture was transferred to a balloon, diluted with ice-cold water (15 mL) and basified with NaOH 6N. The suspension was extracted with ethyl acetate (3 x 15 mL), dried on sodium sulfate and evaporated to obtain the desired compounds which were purified by crystallization from ethanol.

4-Amino-2,6-diphenylpyridazin-3(2H)-one (16a). Yield = 69 %; mp = 216-218 °C (EtOH). ^{1}H NMR ($CDCl_3$) δ 6.75 (s, 1H, pyridazinone), 7.45-7.55 (m, 5H, Ar), 7.72-7.80 (m, 5H, Ar). IR ν (cm^{-1}): 3429-3315 (NH_2), 1615 (CO).

4-Amino-6-(4-fluorophenyl)-2-phenylpyridazin-3(2H)-one (16b). Yield = 71 %; mp = 167-169 °C (EtOH). ^{1}H NMR ($CDCl_3$) δ 6.76 (s, 1H, pyridazinone), 7.13 (t, 2H, Ar, *J* = 8.6 Hz), 7.41 (t, 1H, Ar, *J* = 8.0 Hz), 7.51 (t, 2H, Ar, *J* = 7.6 Hz), 7.72 (d, 2H, Ar, *J* = 7.6 Hz), 7.77-7.81 (m, 2H, Ar). IR ν (cm^{-1}): 3458-3317 (NH_2), 1612 (CO).

General procedure for compounds 17a-c. A mixture of **16a,b** (0.18 mmol), suitable anhydride (6.03 mmol) and 1.5 mL of pyridine was warmed in a sealed tube at 140 °C for 3-5 h. After cooling, the mixture was transferred to a balloon and ice-cold water (15 mL) was added. The mixture was stirred for 1 h about in order to hydrolyze the excess of anhydride. The precipitate formed was recovered by *vacuum* filtration to obtain the desired compounds **17a-c** which were purified by crystallization from ethanol.

N-(3-Oxo-2,6-diphenyl-2,3-dihydropyridazin-4-yl)pentanamide (17a). Yield = 50 %; mp = 120-123 °C (EtOH). ^{1}H NMR ($CDCl_3$) δ 1.00 (t, 3H, $CH_2CH_2CH_2\mathit{CH_3}$, *J* = 7.6 Hz), 1.46 (sest, 2H, $CH_2CH_2\mathit{CH_2}CH_3$, *J* = 7.4 Hz), 1.77 (quin, 2H, $CH_2\mathit{CH_2}CH_2CH_3$, *J* = 7.6 Hz), 2.53 (t, 2H, $\mathit{CH_2}CH_2CH_2CH_3$, *J* = 7.4 Hz), 7.43-7.60 (m, 6H, Ar), 7.73 (d, 2H, Ar, *J* = 7.6 Hz), 7.90 (dd, 2H, Ar, *J* = 1.8 Hz and *J* = 7.4 Hz), 8.74 (exch br s, 1H, NH), 8.77 (s, 1H, pyridazinone).

N-(3-Oxo-2,6-diphenyl-2,3-dihydropyridazin-4-yl)isobutyramide (17b). Yield = 55 %; mp = 167-168 °C (EtOH). ^{1}H NMR ($CDCl_3$) δ 1.32 (d, 6H, $CH\mathit{(CH_3)_2}$, *J* = 6.8 Hz), 2.71 (quin, 1H, $\mathit{CH}(CH_3)_2$, *J* = 6.8 Hz), 7.43-7.48 (m, 4H, Ar), 7.54 (t, 2H, Ar, *J* = 7.2 Hz), 7.73 (d, 2H, Ar, *J* = 8.0 Hz), 7.91 (d, 2H, Ar, *J* = 6.8 Hz), 8.78 (s, 1H, pyridazinone), 8.82 (exch br s, 1H, NH).

N-(6-(4-Fluorophenyl)-3-oxo-2-phenyl-2,3-dihydropyridazin-4-yl)isobutyramide (17c). Yield = 48 %; mp = 169-170 °C (EtOH). ^{1}H NMR ($CDCl_3$) δ 1.32 (d, 6H, CH*(CH_3)₂*, *J* = 6.8 Hz), 2.70 (quin, 1H, *CH*(CH_3)$_2$, *J* = 6.8 Hz), 7.15 (t, 2H, Ar, *J* = 8.4 Hz), 7.46 (t, 1H, Ar, *J* = 7.4 Hz), 7.54 (t, 2H, Ar, *J* = 7.8 Hz), 7.71 (d, 2H, Ar, *J* = 8.0 Hz), 7.86-7.91 (m, 2H, Ar), 8.74 (s, 1H, pyridazinone), 8.82 (exch br s, 1H, NH). LC-MS: 352.18 $[M+H]^+$.

5-Amino-N-(tert-butyl)-6-oxo-3-phenyl-1,6-dihydropyridazine-4-carboxamide (49). A mixture of compound **48**[27] (0.94 mmol) and *tert*-butylamine (2.85 mmol) in 2 mL of anhydrous 1,4-dioxane was warmed in a sealed tube at 80-90 °C for 2 h. After cooling, the mixture was transferred to a balloon and the solvent was evaporated. Ice-cold water (15 mL) was added and the light brown precipitate was filtered off by suction to obtain the desired compound **49**. Yield = 45 %; mp = 229-233 °C (EtOH). ^{1}H NMR (DMSO-d_6) δ 1.08 (s, 9H, C*(CH_3)$_3$*), 6.22 (exch br s, 2H, NH_2), 7.37-7.45 (m, 5H, Ar), 12.79 (exch br s, 1H, NH pyrid.).

General procedure for compounds 18 and 20. A mixture of compounds **49** (for **18**) or **19** (for **20**) (0.24 mmol), K_2CO_3 (0.50 mmol), benzyl chloride (0.30 mmol), and anhydrous acetone (1.5 mL) was stirred at reflux for 2 h. After cooling, the solvent was evaporated and ice-cold water (10 mL) was added. The suspension was extracted with ethyl acetate (3 x 15 mL), dried on sodium sulfate and evaporated to obtain the desired compounds which were purified by crystallization from ethanol for **18**) or methanol (for **20**).

5-Amino-1-benzyl-N-(tert-butyl)-6-oxo-3-phenyl-1,6-dihydropyridazine-4-carboxamide (18). Yield = 54 %; mp = 168-169 °C (EtOH). ^{1}H NMR ($CDCl_3$) δ 1.06 (s, 9H, C*(CH_3)$_3$*), 4.81 (exch br s, 1H, *NH*CO), 5.37 (s, 2H, *CH_2*-Ph), 7.30-7.38 (m, 3H, Ar), 7.45-7.55 (m, 7H, Ar). LC-MS: 377.27 $[M+H]^+$.

Benzyl 5-amino-1-benzyl-6-oxo-3-phenyl-1,6-dihydropyridazine-4-carboxylate (20). Yield = 47 %; mp = 115-116 °C (MeOH). ^{1}H NMR ($CDCl_3$) δ 4.96 (s, 2H, O-*CH_2*-Ph), 5.34 (s, 2H, N-*CH_2*-Ph), 6.78 (d, 2H, Ar, *J* = 7.6 Hz), 7.17-7.24 (m, 3H, Ar), 7.27-7.35 (m, 8H, Ar), 7.47 (d, 2H, Ar, *J* = 7.6 Hz).

Benzyl 5-amino-6-oxo-3-phenyl-1,6-dihydropyridazine-4-carboxylate (19). To a suspension of intermediate **48**[27] (1.41 mmol) in 1.5 mL of benzyl alcohol (14.5 mmol) in a sealed tube, 3.59 mmol of Et_3N was added. The mixture reaction was warmed at 80 °C for 3 h. After cooling in ice-bath, ethanol was added, a light yellow precipitate was formed and it was filtered off by suction to obtain the desired compound. Yield = 35 %; mp = 218-220 °C (EtOH). ^{1}H NMR (DMSO-d_6) δ 5.00 (s, 2H,

O-*CH*$_2$-Ph), 6.87 (d, 2H, Ar, *J* = 7.6 Hz), 7.20-7.35 (m, 8H, Ar), 7.40 (exch br s, 2H, NH_2), 12.91 (exch br s, 1H, NH). IR ν (cm^{-1}): 3451-3327 (NH_2), 1687 (CO), 1658 (CO).

General procedure for compounds 21a-e. Et_3N (1.14 mmol) was added to a cooled (-5 °C) and stirred solution of intermediate **50a,b**[33,34] (0.33 mmol) in anhydrous tetrahydrofuran (2 mL). After 30 min, the mixture was allowed to warm up to 0 °C and ethyl chloroformate (0.36 mmol) was added. After 1 h, the suitable amine (0.69 mmol) was added. The reaction was carried out at room temperature for 12 h. The mixture was then concentrated *in vacuo*, diluted with cold water (10-15 mL) and extracted with CH_2Cl_2 (3 x 15 mL). The organic phase was dried on sodium sulphate and the solvent was evaporated to afford final compounds **21a-e** which were purified by flash column chromatography using cyclohexane/ethyl acetate 1:1 (for **21a-c**) or 2:1 (for **21d**,**e**) as eluents.

2-(6-Oxo-3,4-diphenylpyridazin-1(6H)-yl)-N-propylacetamide (21a). Yield = 35 %; mp = 136-137 °C (EtOH). ^{1}H NMR ($CDCl_3$) δ 0.94 (t, 3H, CH_2CH_2*CH*$_3$, *J* = 7.4 Hz), 1.57 (sest, 2H, CH_2*CH*$_2$$CH_3$, *J* = 7.2 Hz), 3.28 (q, 2H, *CH*$_2$$CH_2CH_3$, *J* = 7.0 Hz), 4.93 (s, 2H, N*CH*$_2$CO), 6.47 (exch br s, 1H, *NH*CO), 7.00-7.40 (m, 11H, 10H Ar + 1H pyridazinone).

N-Isopropyl-2-(6-oxo-3,4-diphenylpyridazin-1(6H)-yl)acetamide (21b). Yield = 44 %; mp = 138-139 °C (EtOH). ^{1}H NMR ($CDCl_3$) δ 1.20 (d, 6H, CH(*CH*$_3$)$_2$, *J* = 6.8 Hz), 4.12 (sest, 1H, *CH*(CH_3)$_2$, *J* = 6.8 Hz), 4.88 (s, 2H, N*CH*$_2$CO), 6.22 (exch br s, 1H, *NH*CO), 7.00 (s, 1H, pyridazinone), 7.05-7.17 (m, 4H, Ar), 7.30-7.40 (m, 6H, Ar).

N-Cyclopentyl-2-(6-oxo-3,4-diphenylpyridazin-1(6H)-yl)acetamide (21c). Yield = 65%; mp = 148-150 °C (EtOH). ^{1}H NMR ($CDCl_3$) δ 1.40-1.50 (m, 2H, cC_5H_9), 1.53-1.73 (m, 3H, cC_5H_9), 1.95-2.08 (m, 3H, cC_5H_9), 4.20-4.30 (m, 1H, CH cC_5H_9), 4.89 (s, 2H, NCH_2CO), 6.50 (exch br s, 1H, *NH*CO), 7.00 (s, 1H, pyridazinone), 7.05-7.18 (m, 5H, Ar), 7.20-7.40 (m, 5H, Ar).

2-(6-Oxo-3,5-diphenylpyridazin-1(6H)-yl)-N-propylacetamide (21d). Yield = 22 %; mp = 168-169 °C (EtOH). ^{1}H NMR ($CDCl_3$) δ 0.92 (t, 3H, CH_2CH_2*CH*$_3$, *J* = 7.4 Hz), 1.57 (sest, 2H, CH_2*CH*$_2$$CH_3$, *J* = 7.4 Hz), 3.27 (q, 2H, *CH*$_2$$CH_2CH_3$, *J* = 6.8 Hz), 5.00 (s, 2H, N*CH*$_2$CO), 6.48 (exch br s, 1H, *NH*CO), 7.48-7.55 (m, 6H, 5H Ar + 1H pyridazinone), 7.84-7.90 (m, 5H, Ar).

N-Isopropyl-2-(6-oxo-3,5-diphenylpyridazin-1(6H)-yl)acetamide (21e). Yield = 34 %; mp = 198-200 °C (EtOH). ^{1}H NMR ($CDCl_3$) δ 1.18 (d, 6H, CH(*CH*$_3$)$_2$, *J* = 6.8 Hz), 4.12 (sest, 1H, *CH*(CH_3)$_2$, *J* = 6.8 Hz), 4.96 (s, 2H, N*CH*$_2$CO), 6.26 (exch br s, 1H, *NH*CO), 7.46-7.51 (m, 6H, 5H Ar + 1H pyridazinone), 7.84-7.90 (m, 5H, Ar).

3. Experimental Details - Molecular modelling

Table S1. PharmMapper result for molecule **1a**.

	PDB ID	Target Name	Fit Score	Normalized Fit Score
1	1LFG	Lactotransferrin	2.98	0.9935
2	2GWF	Ubiquitin carboxyl-terminal hydrolase 8	2.977	0.9923
3	4LUR	NONE	2.961	0.9871
4	3CR3	PTS-dependent dihydroxyacetone kinase, ADP-binding subunit dhaL	2.929	0.9764
5	1NU9	Prothrombin	3.845	0.769
6	1RF8	Eukaryotic translation initiation factor 4E	2.996	0.7491
7	2CST	Aspartate aminotransferase, cytoplasmic	2.992	0.748
8	1ZR4	Transposon gamma-delta resolvase	2.992	0.7479
9	2AJ7	Flagellar assembly factor fliW	2.991	0.7477
10	1MKM	Transcriptional regulator, IclR family	2.981	0.7452
11	2GFP	Multidrug resistance protein D	2.98	0.7451
12	2V0V	Nuclear receptor subfamily 1 group D member 2	2.977	0.7443
13	1IS2	Acyl-coenzyme A oxidase 1, peroxisomal	2.976	0.7439
14	2C9O	RuvB-like 1	2.975	0.7437
15	2PJW	Class E vacuolar protein-sorting machinery protein HSE1	2.963	0.7407
16	2E8X	Geranylgeranyl pyrophosphate synthetase	2.951	0.7377
17	2AD9	Polypyrimidine tract-binding protein 1	2.949	0.7372
18	1SSE	AP-1-like transcription factor YAP1	2.945	0.7362
19	1S7Q	H-2 class I histocompatibility antigen, K-B alpha chain	2.945	0.7362
20	1R6R	Genome polyprotein	2.944	0.7359
21	1W36	Exodeoxyribonuclease V gamma chain	2.937	0.7344
22	1UJV	Membrane-associated guanylate kinase, WW and PDZ domain-containing protein 2	2.937	0.7342
23	1W1G	3-phosphoinositide-dependent protein kinase 1	2.937	0.7342
24	2E02	Cysteine proteinase 1, mitochondrial	2.932	0.7329
25	1CLZ	Ig gamma-3 chain C region	2.932	0.7329
26	1QZ2	FK506-binding protein 4	2.925	0.7313
27	1IG8	Hexokinase-2	2.923	0.7308
28	2P0W	Histone acetyltransferase type B catalytic subunit	2.921	0.7302
29	2B6E	Putative esterase HI1161	2.915	0.7289
30	1PC8	Beta-galactoside-specific lectin 4	2.91	0.7274
31	1UKF	Cysteine protease avirulence protein avrPphB	4	0.6667
32	1S40	Cell division control protein 13	3.915	0.6525
33	3FQD	5-3 exoribonuclease 2	3.133	0.6266
34	2ZU6	Eukaryotic initiation factor 4A-I	3.745	0.6241
35	1HM9	Bifunctional protein glmU	3.12	0.624
36	1X65	Cold shock domain-containing protein E1	3.7	0.6167
37	1XJV	Protection of telomeres protein 1	3.067	0.6133
38	2VH3	Ranasmurfin	3	0.6

39	1PHN	C-phycocyanin alpha chain	3	0.6
40	1WIN	Flotillin-2	3	0.6
41	1ZVS	Beta-2-microglobulin	3	0.6
42	2P0R	Calpain-9	2.998	0.5996
43	1DUS	Protein MJ0882	3.597	0.5995
44	2FV4	Kinetochore protein SPC25	2.993	0.5987
45	1BUO	Zinc finger and BTB domain-containing protein 16	2.993	0.5985
46	2I15	Uncharacterized protein MG296 homolog	2.99	0.5981
47	1JN5	NTF2-related export protein 1	2.988	0.5975
48	1FNC	Ferredoxin--NADP reductase, chloroplastic	2.987	0.5974
49	1LSH	Vitellogenin	2.985	0.5971
50	1GHS	Glucan endo-1,3-beta-glucosidase GII	2.982	0.5964

Table S2. PharmMapper result for molecule **2a**.

	PDB ID	Target Name	Fit Score	Normalized Fit Score
1	4NFT	NONE	2.996	0.9986
2	2JQD	Acidic leucine-rich nuclear phosphoprotein 32 family member A	2.994	0.9981
3	2IRP	Methylthioribulose-1-phosphate dehydratase	2.994	0.998
4	1T0T	UPF0447 protein GK3416	2.994	0.9979
5	2NQ2	Probable ABC transporter permease protein HI1471	2.994	0.9979
6	1CQA	Profilin	2.993	0.9975
7	2VRW	Ras-related C3 botulinum toxin substrate 1	2.992	0.9975
8	1IJE	Elongation factor 1-alpha	2.992	0.9975
9	2HDP	E3 ubiquitin-protein ligase Mdm2	2.992	0.9975
10	1GK5	Pro-epidermal growth factor	2.992	0.9974
11	2KDD	Borealin	2.992	0.9973
12	3CE6	Adenosylhomocysteinase	2.991	0.9969
13	2DGR	RNA-binding protein MEX3D	2.99	0.9966
14	1M3S	3-hexulose-6-phosphate isomerase	2.99	0.9965
15	1O96	Electron transfer flavoprotein subunit beta	2.989	0.9965
16	2PJW	Class E vacuolar protein-sorting machinery protein HSE1	2.989	0.9963
17	3EPY	Acyl-CoA-binding domain-containing protein 7	2.989	0.9962
18	1O7D	Lysosomal alpha-mannosidase	2.989	0.9962
19	1S68	RNA ligase 2	2.988	0.996
20	1VLI	Spore coat polysaccharide biosynthesis protein spsE	2.988	0.9959
21	3DZ7	S-adenosylmethionine decarboxylase proenzyme	2.987	0.9956
22	2GWF	Ubiquitin carboxyl-terminal hydrolase 8	2.986	0.9954
23	1V5V	Probable aminomethyltransferase	2.986	0.9953
24	2ASF	Uncharacterized protein Rv2074/MT2134	2.986	0.9952
25	1Z0D	Ras-related protein Rab-5C	2.985	0.9951
26	1O9Y	Type III secretion protein hrcQb	2.985	0.9949
27	2NPU	FKBP12-rapamycin complex-associated protein	2.984	0.9948
28	3DUZ	Major envelope glycoprotein	2.984	0.9947
29	2A7S	Probable propionyl-CoA carboxylase beta chain 5	2.984	0.9945

30	2I15	Uncharacterized protein MG296 homolog	2.983	0.9945
31	1QWG	Phosphosulfolactate synthase	2.983	0.9943
32	1U2W	Cadmium efflux system accessory protein	2.983	0.9943
33	2PIH	Uncharacterized protein ymcA	2.983	0.9942
34	1J1E	Troponin C, slow skeletal and cardiac muscles	2.981	0.9936
35	2D6F	Glutamyl-tRNA(Gln) amidotransferase subunit D	2.981	0.9935
36	1QZT	Phosphate acetyltransferase	2.98	0.9934
37	1F3B	Glutathione S-transferase A1	2.98	0.9932
38	3HZQ	Large-conductance mechanosensitive channel	2.979	0.993
39	2ZIW	Crossover junction endonuclease MUS81	2.979	0.993
40	1UKK	Osmotically inducible protein C	2.975	0.9917
41	1GGZ	Calmodulin-like protein 3	2.973	0.9912
42	1D4M	Genome polyprotein	2.973	0.9908
43	2ZSM	Glutamate-1-semialdehyde 2,1-aminomutase	2.972	0.9908
44	1BDG	Hexokinase	2.971	0.9904
45	2CWX	Ribulose bisphosphate carboxylase	2.965	0.9884
46	2I1Y	Receptor-type tyrosine-protein phosphatase-like N	2.965	0.9884
47	1XPP	DNA-directed RNA polymerase subunit L	2.965	0.9883
48	2P67	LAO/AO transport system kinase	2.964	0.9881
49	2UUU	Alkyldihydroxyacetonephosphate synthase	2.963	0.9876
50	1GT5	Odorant-binding protein	2.962	0.9874

Table S3. PharmMapper result for molecule **3a**.

	PDB ID	Target Name	Fit Score	Normalized Fit Score
1	3MYB	NONE	2.993	0.7482
2	3B3D	Uncharacterized oxidoreductase ytbE	2.985	0.7463
3	2NVB	NADP-dependent alcohol dehydrogenase	2.98	0.745
4	1UWK	Urocanate hydratase	2.966	0.7416
5	1UJV	Membrane-associated guanylate kinase, WW and PDZ domain-containing protein 2	2.963	0.7407
6	1EYB	Homogentisate 1,2-dioxygenase	2.949	0.7371
7	2NZ2	Argininosuccinate synthase	2.948	0.737
8	2EBZ	Regulator of G-protein signaling 12	2.947	0.7369
9	1U2E	2-hydroxy-6-oxononadienedioate/2-hydroxy-6-oxononatrienedioate hydrolase	2.93	0.7325
10	2CO9	Thymocyte selection-associated high mobility group box protein TOX	2.92	0.7301
11	1A0J	Trypsin-3	2.919	0.7298
12	2GNX	UPF0536 protein C12orf66 homolog	2.919	0.7297
13	2DBR	Glyoxylate reductase	2.917	0.7293
14	1GYP	Glyceraldehyde-3-phosphate dehydrogenase, glycosomal	2.91	0.7275
15	2ZPA	Uncharacterized protein ypfI	2.909	0.7272
16	2ZW9	Leucine carboxyl methyltransferase 2	2.907	0.7268
17	2PFC	Uncharacterized protein Rv0098/MT0107	2.906	0.7266
18	3DRA	Protein farnesyltransferase/geranylgeranyltransferase type-1 subunit alpha	2.903	0.7259

19	3DXR	Mitochondrial import inner membrane translocase subunit TIM9	2.896	0.7241
20	1UYR	Acetyl-CoA carboxylase	2.895	0.7238
21	3FFV	Protein syd	2.883	0.7208
22	2YRV	AT-rich interactive domain-containing protein 4A	2.883	0.7208
23	1JL2	Ribonuclease HI	2.882	0.7205
24	2CWX	Ribulose bisphosphate carboxylase	2.876	0.719
25	1TFC	Estrogen-related receptor gamma	2.869	0.7173
26	1W07	Acyl-coenzyme A oxidase 1, peroxisomal	2.868	0.717
27	2AQT	Superoxide dismutase [Cu-Zn]	2.86	0.715
28	2ROD	Induced myeloid leukemia cell differentiation protein Mcl-1 homolog	2.845	0.7112
29	2RGV	Peroxide operon regulator	2.841	0.7102
30	2COP	Acyl-CoA-binding domain-containing protein 6	2.839	0.7096
31	1KA9	Imidazole glycerol phosphate synthase subunit hisF	2.838	0.7096
32	1D2E	Elongation factor Tu, mitochondrial	2.837	0.7092
33	1PN0	Phenol 2-monooxygenase	2.828	0.7071
34	1B93	Methylglyoxal synthase	2.827	0.7067
35	2QG3	UPF0130 protein AF_2059	2.814	0.7035
36	2VRE	Delta(3,5)-Delta(2,4)-dienoyl-CoA isomerase, mitochondrial	2.813	0.7032
37	2I99	Mu-crystallin homolog	2.804	0.7009
38	2P0W	Histone acetyltransferase type B catalytic subunit	2.799	0.6997
39	1I3A	Ribonuclease HII	2.797	0.6992
40	1WYM	Transgelin-2	2.796	0.699
41	2JZ6	50S ribosomal protein L28	2.787	0.6968
42	1KZF	Acyl-homoserine-lactone synthase	2.78	0.6951
43	1JQK	Carbon monoxide dehydrogenase	2.77	0.6925
44	2CST	Aspartate aminotransferase, cytoplasmic	2.769	0.6924
45	1U94	Protein recA	2.758	0.6894
46	1WFH	Zinc finger A20 and AN1 domain-containing stress-associated protein 4	2.75	0.6875
47	3D3L	Arachidonate 12-lipoxygenase, 12S-type	3.183	0.6366
48	2YWW	Aspartate carbamoyltransferase regulatory chain	2.991	0.5983
49	1URJ	Major DNA-binding protein	2.985	0.597
50	1B7A	Phosphatidylethanolamine-binding protein 1	2.984	0.5968

Table S4. PharmMapper result for molecule **5a**.

	PDB ID	Target Name	Fit Score	Normalized Fit Score
1	1VBI	Dehydrogenase	2.961	0.9871
2	3D31	NONE	2.996	0.7489
3	1YAA	Aspartate aminotransferase, cytoplasmic	2.991	0.7478
4	1J3N	Transferase	2.991	0.7477

5	2DQN	Glutamyl-tRNA(Gln) amidotransferase subunit A	2.973	0.7432
6	3BHY	Death-associated protein kinase 3	2.971	0.7427
7	3HKI	Prothrombin	2.933	0.7332
8	1D06	Sensor protein fixL	2.905	0.7262
9	2OWI	Regulator of G-protein signaling 18	2.899	0.7246
10	3CA8	Protein ydcF	2.894	0.7234
11	2VLD	UPF0286 protein PYRAB01260	2.887	0.7218
12	3DRA	Protein farnesyltransferase/geranylgeranyltransferase type-1 subunit alpha	2.863	0.7157
13	3B8C	ATPase 2, plasma membrane-type	2.849	0.7123
14	2NRO	Molybdopterin biosynthesis protein moeA	2.832	0.7079
15	2CR7	Paired amphipathic helix protein Sin3b	2.824	0.7061
16	2VRE	Delta(3,5)-Delta(2,4)-dienoyl-CoA isomerase, mitochondrial	2.818	0.7046
17	3CPR	Dihydrodipicolinate synthase	2.81	0.7026
18	2I15	Uncharacterized protein MG296 homolog	2.795	0.6988
19	1JQK	Carbon monoxide dehydrogenase	2.794	0.6986
20	2PUJ	2-hydroxy-6-oxo-6-phenylhexa-2,4-dienoate hydrolase	2.786	0.6965
21	1PC8	Beta-galactoside-specific lectin 4	2.765	0.6914
22	2GQF	Uncharacterized protein HI0933	2.709	0.6771
23	1NF0	Triosephosphate isomerase	2.704	0.676
24	2GNX	UPF0536 protein C12orf66 homolog	2.695	0.6738
25	1LJ0	Cytochrome b5 type B	2.684	0.6709
26	1K7A	Protein C-ets-1	2.653	0.6632
27	2JWE	Tight junction protein ZO-1	2.609	0.6522
28	2QFD	Probable ATP-dependent RNA helicase DDX58	2.608	0.6519
29	1U94	Protein recA	2.607	0.6518
30	2JZ6	50S ribosomal protein L28	2.556	0.639
31	3FFV	Protein syd	2.529	0.6323
32	1PN0	Phenol 2-monooxygenase	2.437	0.6093
33	2Q2E	Type II DNA topoisomerase VI subunit A	2.431	0.6077
34	3FMO	Nuclear pore complex protein Nup214	2.42	0.6049
35	2AQT	Superoxide dismutase [Cu-Zn]	2.4	0.6
36	1GGZ	Calmodulin-like protein 3	2.998	0.5997
37	2BTY	Acetylglutamate kinase	2.998	0.5996
38	1NA6	Type-2 restriction enzyme EcoRII	2.995	0.5989
39	1FI6	RalBP1-associated Eps domain-containing protein 1	2.995	0.5989
40	3EAP	Rho GTPase-activating protein 11A	2.994	0.5989
41	2AY0	Bifunctional protein putA	2.994	0.5988
42	1SNL	Nucleobindin-1	2.993	0.5987
43	1RKS	Ribokinase	2.989	0.5979
44	1UFI	Major centromere autoantigen B	2.983	0.5965
45	2HJS	USG-1 protein homolog	2.975	0.595
46	1XCB	Redox-sensing transcriptional repressor rex	2.97	0.594
47	1IVX	Phenylethylamine oxidase	2.969	0.5938
48	3G9K	Capsule biosynthesis protein capD	2.965	0.5931

49	2VWT	2-keto-3-deoxy-L-rhamnonate aldolase	2.961	0.5922
50	2HHK	Reaction center protein H chain	2.958	0.5917

Table S5. PharmMapper result for molecule **7**.

	PDB ID	Target Name	Fit Score	Normalized Fit Score
1	4I1P	NONE	3.171	0.7929
2	2CST	Aspartate aminotransferase, cytoplasmic	2.981	0.7454
3	3FMO	Nuclear pore complex protein Nup214	2.903	0.7257
4	3DRA	Protein farnesyltransferase/geranylgeranyltransferase type-1 subunit alpha	2.862	0.7154
5	1JQK	Carbon monoxide dehydrogenase	2.858	0.7145
6	1PP9	Cytochrome b-c1 complex subunit 1, mitochondrial	2.752	0.688
7	2VRE	Delta(3,5)-Delta(2,4)-dienoyl-CoA isomerase, mitochondrial	2.741	0.6852
8	2GQF	Uncharacterized protein HI0933	2.702	0.6754
9	1QGK	Importin subunit beta-1	2.658	0.6645
10	1PN0	Phenol 2-monooxygenase	2.619	0.6548
11	1XQ1	Tropinone reductase homolog At1g07440	2.538	0.6346
12	2C6S	Penton protein	2.425	0.6062
13	2CWX	Ribulose bisphosphate carboxylase	2.367	0.5918
14	1QRJ	Gag-Pro-Pol polyprotein	2.361	0.5901
15	1GPM	GMP synthase [glutamine-hydrolyzing]	2.943	0.5886
16	1UYR	Acetyl-CoA carboxylase	2.301	0.5754
17	2P1Q	SKP1-like protein 1A	2.866	0.5732
18	1YIV	Myelin P2 protein	4.559	0.5699
19	1FRV	Periplasmic [NiFe] hydrogenase small subunit	2.268	0.5671
20	1SGJ	Citrate lyase beta subunit-like protein	2.267	0.5666
21	2PA6	Enolase	2.266	0.5664
22	1YPF	GMP reductase	2.811	0.5621
23	1Y1U	Signal transducer and activator of transcription 5A	2.81	0.5621
24	1R6U	Tryptophanyl-tRNA synthetase, cytoplasmic	2.235	0.5587
25	1I3R	H-2 class II histocompatibility antigen, E-K alpha chain	2.788	0.5576
26	2P0R	Calpain-9	2.744	0.5488
27	2E55	Uracil phosphoribosyltransferase	3.289	0.5482
28	1SPU	Primary amine oxidase	3.247	0.5411
29	2QFD	Probable ATP-dependent RNA helicase DDX58	2.162	0.5405
30	1OXY	Hemocyanin II	3.225	0.5375
31	1HRO	Cytochrome c2	3.211	0.5352
32	1W9C	Exportin-1	2.14	0.5351
33	1B35	Genome polyprotein	2.125	0.5313
34	1MDZ	UDP-4-amino-4-deoxy-L-arabinose--oxoglutarate aminotransferase	2.101	0.5254
35	1RP8	Alpha-amylase type A isozyme	4.19	0.5238
36	1VLU	Gamma-glutamyl phosphate reductase	2.614	0.5229

37	3ESW	Peptide-N(4)-(N-acetyl-beta-glucosaminyl)asparagine amidase	2.608	0.5216
38	1PUJ	Ribosome biogenesis GTPase A	3.112	0.5186
39	3G2F	Bone morphogenetic protein receptor type-2	2.584	0.5169
40	1IS2	Acyl-coenzyme A oxidase 1, peroxisomal	3.098	0.5163
41	1AHS	Core protein VP7	3.097	0.5161
42	1QLM	Methenyltetrahydromethanopterin cyclohydrolase	2.559	0.5118
43	1Y79	Peptidyl-dipeptidase dcp	2.559	0.5117
44	1V5V	Probable aminomethyltransferase	2.033	0.5083
45	1X62	PDZ and LIM domain protein 1	2.535	0.5071
46	1J4A	D-lactate dehydrogenase	2.534	0.5068
47	2UUU	Alkyldihydroxyacetonephosphate synthase	2.022	0.5055
48	1RVV	6,7-dimethyl-8-ribityllumazine synthase	2.523	0.5046
49	1SNL	Nucleobindin-1	2.48	0.496
50	1XDO	Polyphosphate kinase	2.479	0.4958

Table S6. PharmMapper result for molecule **8**.

	PDB ID	Target Name	Fit Score	Normalized Fit Score
1	2C9O	RuvB-like 1	3.118	0.7794
2	1DK8	Axin-1	3.824	0.7647
3	4HNY	NONE	3.796	0.7593
4	3F3K	Uncharacterized protein YKR043C	3.404	0.6808
5	1XES	Dihydropinosylvin synthase	3.393	0.6785
6	1RQG	Methionyl-tRNA synthetase	3.383	0.6766
7	2BTY	Acetylglutamate kinase	3.285	0.657
8	3D3L	Arachidonate 12-lipoxygenase, 12S-type	3.204	0.6408
9	3CLH	3-dehydroquinate synthase	3.128	0.6256
10	2R0N	Glutaryl-CoA dehydrogenase, mitochondrial	3.128	0.6256
11	2HMA	tRNA-specific 2-thiouridylase mnmA	3.727	0.6212
12	1H4R	Merlin	3.712	0.6186
13	1U08	Aminotransferase ybdL	3.072	0.6144
14	1X65	Cold shock domain-containing protein E1	3.681	0.6135
15	2KA5	Putative anti-sigma factor antagonist TM_1081	3.651	0.6084
16	3C7G	Arabinoxylan arabinofuranohydrolase	3.628	0.6047
17	1LRW	Methanol dehydrogenase subunit 1	3.62	0.6033
18	2BTU	Phosphoribosylformylglycinamidine cyclo-ligase	3.618	0.603
19	1TU9	Hypothetical protein	3.611	0.6019
20	1HJ1	Estrogen receptor beta	3.603	0.6005
21	1S40	Cell division control protein 13	3.554	0.5923
22	2IN5	Uncharacterized lipoprotein gfcB	3.546	0.5909
23	1UKF	Cysteine protease avirulence protein avrPphB	3.534	0.589
24	1YY7	Regulator of transcription; stringent starvation protein A	3.51	0.5851
25	1LWD	Isocitrate dehydrogenase [NADP], mitochondrial	3.501	0.5836
26	2A7S	Probable propionyl-CoA carboxylase beta chain 5	3.495	0.5826

27	2P4B	Sigma-E factor regulatory protein rseB	3.476	0.5794
28	3DL2	Ubiquitin-conjugating enzyme E2 variant 3	3.469	0.5781
29	2GK9	Phosphatidylinositol-5-phosphate 4-kinase type-2 gamma	3.468	0.578
30	2GU0	Non-structural protein 2	3.444	0.574
31	1IN7	Holliday junction ATP-dependent DNA helicase ruvB	3.442	0.5737
32	3DH4	Sodium/glucose cotransporter	3.424	0.5707
33	1MJ3	Enoyl-CoA hydratase, mitochondrial	3.407	0.5678
34	2RLI	Protein SCO2 homolog, mitochondrial	3.395	0.5658
35	1K1G	Splicing factor 1	3.392	0.5653
36	1V1F	Calcineurin B-like protein 4	3.368	0.5613
37	1MA1	Superoxide dismutase [Fe]	3.363	0.5605
38	2E74	Cytochrome b6	3.352	0.5586
39	1S0W	Beta-lactamase TEM	3.348	0.558
40	2HCB	Chromosomal replication initiator protein dnaA	3.899	0.557
41	1OVQ	Putative Holliday junction resolvase	3.335	0.5559
42	2CSH	Zinc finger and BTB domain-containing protein 43	3.89	0.5558
43	2IF2	Dephospho-CoA kinase	3.322	0.5537
44	2QTZ	Methionine synthase reductase, mitochondrial	3.308	0.5514
45	1P6P	Fatty acid-binding protein, liver	3.845	0.5493
46	3DP9	5-methylthioadenosine/S-adenosylhomocysteine nucleosidase	3.287	0.5479
47	2C7Y	3-ketoacyl-CoA thiolase 2, peroxisomal	3.824	0.5462
48	2RC7	Glutamate [NMDA] receptor subunit 3A	3.273	0.5455
49	2Z2N	Virginiamycin B lyase	3.815	0.545
50	1CO6	Cytochrome c2	3.269	0.5449

Table S7. PharmMapper result for molecule **9**.

	PDB ID	Target Name	Fit Score	Normalized Fit Score
1	4UAK	NONE	3.57	0.8926
2	1FFT	Ubiquinol oxidase subunit 1	3.444	0.861
3	2NRO	Molybdopterin biosynthesis protein moeA	3.306	0.8265
4	1R1T	Transcriptional repressor smtB	3.266	0.8165
5	2OWI	Regulator of G-protein signaling 18	3.204	0.8011
6	3FQD	5-3 exoribonuclease 2	3.943	0.7885
7	1YED	Ig gamma-1 chain C region secreted form	3.087	0.7718
8	2F8N	Histone H3.2	3.762	0.7525
9	1XES	Dihydropinosylvin synthase	3.751	0.7502
10	1DK8	Axin-1	3.75	0.7501
11	1X65	Cold shock domain-containing protein E1	4.472	0.7453
12	2YWW	Aspartate carbamoyltransferase regulatory chain	3.689	0.7378
13	2ES0	Regulator of G-protein signaling 6	3.665	0.7331
14	3EXA	tRNA Delta(2)-isopentenylpyrophosphate transferase	3.639	0.7278
15	1N6B	Cytochrome P450 2C5	3.62	0.724
16	1XJV	Protection of telomeres protein 1	3.618	0.7236

17	2R0N	Glutaryl-CoA dehydrogenase, mitochondrial	3.593	0.7185
18	2AXX	Cytochrome b5	3.57	0.714
19	1R8U	Cbp/p300-interacting transactivator 2	3.555	0.7109
20	1VF5	Cytochrome b6	3.549	0.7097
21	1M1J	Fibrinogen alpha chain	3.544	0.7088
22	1S99	Putative HMP/thiamine-binding protein ykoF	3.515	0.7031
23	1RQG	Methionyl-tRNA synthetase	3.484	0.6967
24	2P0T	UPF0307 protein PSPTO_4464	3.482	0.6965
25	1GGT	Coagulation factor XIII A chain	3.473	0.6946
26	3D3L	Arachidonate 12-lipoxygenase, 12S-type	3.428	0.6856
27	2H08	Ribose-phosphate pyrophosphokinase 1	3.408	0.6815
28	1SFK	Genome polyprotein	3.364	0.6728
29	1ZTE	Superoxide dismutase [Mn], mitochondrial	3.304	0.6608
30	3F3K	Uncharacterized protein YKR043C	3.284	0.6569
31	2VOJ	Alanine dehydrogenase	3.246	0.6493
32	1WEX	Heterogeneous nuclear ribonucleoprotein L-like	3.24	0.6479
33	2FBW	Succinate dehydrogenase [ubiquinone] flavoprotein subunit, mitochondrial	3.235	0.6469
34	1S40	Cell division control protein 13	3.864	0.6441
35	1L0V	Fumarate reductase flavoprotein subunit	3.221	0.6441
36	1B0A	Bifunctional protein folD	3.212	0.6425
37	2GFP	Multidrug resistance protein D	3.212	0.6425
38	3EPY	Acyl-CoA-binding domain-containing protein 7	3.845	0.6408
39	2JUL	Calsenilin	3.841	0.6401
40	2CTQ	DnaJ homolog subfamily C member 12	4.477	0.6395
41	2QKD	Zinc finger protein ZPR1	3.833	0.6389
42	2QBY	Cell division control protein 6 homolog 1	3.16	0.6321
43	2HYD	Putative multidrug export ATP-binding/permease protein SAV1866	3.773	0.6289
44	3DF0	Calpain-2 catalytic subunit	3.142	0.6283
45	2VIG	Short-chain specific acyl-CoA dehydrogenase, mitochondrial	3.14	0.6279
46	3IBV	Exportin-T	3.131	0.6262
47	1NSH	Protein S100-A11	3.123	0.6247
48	2GK9	Phosphatidylinositol-5-phosphate 4-kinase type-2 gamma	3.745	0.6242
49	1YF6	Reaction center protein H chain	3.121	0.6241
50	2AYN	Ubiquitin carboxyl-terminal hydrolase 14	3.116	0.6232

Table S8. PharmMapper result for molecule **10b**.

	PDB ID	Target Name	Fit Score	Normalized Fit Score
1	1VBI	Dehydrogenase	2.944	0.9812
2	3REO	NONE	2.941	0.9805
3	1UKK	Osmotically inducible protein C	2.926	0.9752
4	2GWF	Ubiquitin carboxyl-terminal hydrolase 8	2.912	0.9707
5	2I15	Uncharacterized protein MG296 homolog	2.899	0.9665

6	2GZA	Type IV secretion system protein virB11	2.881	0.9604
7	2CTK	Vigilin	2.854	0.9513
8	3B8C	ATPase 2, plasma membrane-type	2.998	0.7495
9	2CST	Aspartate aminotransferase, cytoplasmic	2.996	0.7489
10	1D06	Sensor protein fixL	2.99	0.7476
11	2CR7	Paired amphipathic helix protein Sin3b	2.987	0.7469
12	2DQN	Glutamyl-tRNA(Gln) amidotransferase subunit A	2.986	0.7465
13	1LJ0	Cytochrome b5 type B	2.984	0.7461
14	3BHY	Death-associated protein kinase 3	2.976	0.744
15	2VRE	Delta(3,5)-Delta(2,4)-dienoyl-CoA isomerase, mitochondrial	2.974	0.7435
16	2OWI	Regulator of G-protein signaling 18	2.966	0.7414
17	2NRO	Molybdopterin biosynthesis protein moeA	2.957	0.7392
18	3HKI	Prothrombin	2.954	0.7384
19	1P35	Early 35 kDa protein	2.952	0.7381
20	1Y89	DevB protein	2.945	0.7362
21	1J3N	Transferase	2.942	0.7355
22	2HGK	Uncharacterized protein yqcC	2.93	0.7326
23	3EMJ	Inorganic pyrophosphatase	2.929	0.7323
24	2V0V	Nuclear receptor subfamily 1 group D member 2	2.929	0.7322
25	1LSH	Vitellogenin	2.928	0.7319
26	1MKM	Transcriptional regulator, IclR family	2.927	0.7318
27	2VEQ	Centromere DNA-binding protein complex CBF3 subunit B	2.919	0.7298
28	1KU5	Archaeal histone A	2.919	0.7297
29	1A6Z	Hereditary hemochromatosis protein	2.915	0.7288
30	2E02	Cysteine proteinase 1, mitochondrial	2.915	0.7287
31	2GFP	Multidrug resistance protein D	2.913	0.7284
32	1W5A	Cell division protein ftsZ homolog 1	2.909	0.7273
33	1A0J	Trypsin-3	2.908	0.727
34	1YED	Ig gamma-1 chain C region secreted form	2.904	0.726
35	1E00	Odorant-binding protein	2.901	0.7251
36	2I9X	Putative septation protein spoVG	2.898	0.7245
37	2CWX	Ribulose bisphosphate carboxylase	2.897	0.7242
38	2QFD	Probable ATP-dependent RNA helicase DDX58	2.896	0.724
39	2GNX	UPF0536 protein C12orf66 homolog	2.896	0.724
40	2JWE	Tight junction protein ZO-1	2.895	0.7236
41	3DWL	Actin-related protein 3	2.894	0.7234
42	3D64	Adenosylhomocysteinase	2.89	0.7225
43	2OPX	Lactaldehyde dehydrogenase	2.887	0.7218
44	2V3C	Signal recognition particle 19 kDa protein	2.881	0.7203
45	2FM8	Surface presentation of antigens protein spaK	2.881	0.7202
46	1EYB	Homogentisate 1,2-dioxygenase	2.879	0.7199
47	2C9O	RuvB-like 1	2.866	0.7165
48	3DRA	Protein farnesyltransferase/geranylgeranyltransferase type-1 subunit alpha	2.864	0.716
49	1R1T	Transcriptional repressor smtB	2.863	0.7157
50	1RF8	Eukaryotic translation initiation factor 4E	2.858	0.7145

Table S9. PharmMapper result for molecule **10g**.

	PDB ID	Target Name	Fit Score	Normalized Fit Score
1	1VBI	Dehydrogenase	2.989	0.9964
2	2CT7	RING finger protein 31	2.985	0.9948
3	3OK8	NONE	2.976	0.9921
4	2UXW	Very long-chain specific acyl-CoA dehydrogenase, mitochondrial	2.959	0.9864
5	1EVY	Glycerol-3-phosphate dehydrogenase [NAD+], glycosomal	2.957	0.9858
6	1UKK	Osmotically inducible protein C	2.924	0.9747
7	2I15	Uncharacterized protein MG296 homolog	2.903	0.9676
8	2GWF	Ubiquitin carboxyl-terminal hydrolase 8	2.899	0.9662
9	2GZA	Type IV secretion system protein virB11	2.889	0.9629
10	1ROZ	Deoxyhypusine synthase	2.885	0.9616
11	2CTK	Vigilin	2.88	0.9601
12	1YED	Ig gamma-1 chain C region secreted form	2.996	0.749
13	2CST	Aspartate aminotransferase, cytoplasmic	2.994	0.7485
14	2QTZ	Methionine synthase reductase, mitochondrial	2.985	0.7462
15	1SH4	Cytochrome b5	2.983	0.7458
16	1LJ0	Cytochrome b5 type B	2.981	0.7454
17	2OWI	Regulator of G-protein signaling 18	2.972	0.743
18	2NRO	Molybdopterin biosynthesis protein moeA	2.965	0.7413
19	2VRE	Delta(3,5)-Delta(2,4)-dienoyl-CoA isomerase, mitochondrial	2.957	0.7392
20	2JUB	Internal protein I	2.953	0.7382
21	1Q2Y	Uncharacterized N-acetyltransferase yjcF	2.942	0.7354
22	2E02	Cysteine proteinase 1, mitochondrial	2.938	0.7344
23	1Y89	DevB protein	2.935	0.7337
24	1MKM	Transcriptional regulator, IclR family	2.93	0.7326
25	2V0V	Nuclear receptor subfamily 1 group D member 2	2.93	0.7326
26	3EMJ	Inorganic pyrophosphatase	2.93	0.7324
27	1LSH	Vitellogenin	2.929	0.7321
28	3HKI	Prothrombin	2.926	0.7315
29	1J3N	Transferase	2.925	0.7314
30	2CR7	Paired amphipathic helix protein Sin3b	2.916	0.7291
31	1ZRT	Ubiquinol-cytochrome c reductase iron-sulfur subunit	2.916	0.729
32	2C9O	RuvB-like 1	2.915	0.7288
33	2FM8	Surface presentation of antigens protein spaK	2.914	0.7285
34	2OPX	Lactaldehyde dehydrogenase	2.913	0.7282
35	1W5A	Cell division protein ftsZ homolog 1	2.912	0.7281
36	1KU5	Archaeal histone A	2.91	0.7274
37	3DWL	Actin-related protein 3	2.906	0.7265
38	2I9X	Putative septation protein spoVG	2.906	0.7264
39	3D64	Adenosylhomocysteinase	2.904	0.7261

40	1IEB	H-2 class II histocompatibility antigen, E-D alpha chain	2.904	0.726
41	1VB6	Heme-regulated cyclic AMP phosphodiesterase	2.898	0.7245
42	1E00	Odorant-binding protein	2.894	0.7236
43	2GFP	Multidrug resistance protein D	2.892	0.7229
44	2QFD	Probable ATP-dependent RNA helicase DDX58	2.891	0.7228
45	3B8C	ATPase 2, plasma membrane-type	2.89	0.7225
46	1XES	Dihydropinosylvin synthase	3.61	0.722
47	2IX4	3-oxoacyl-[acyl-carrier-protein] synthase, mitochondrial	2.888	0.722
48	1OSA	Calmodulin	2.878	0.7194
49	1WVC	Glucose-1-phosphate cytidylyltransferase	2.875	0.7188
50	2HGK	Uncharacterized protein yqcC	2.873	0.7181

Table S10. PharmMapper result for molecule **11**.

	PDB ID	Target Name	Fit Score	Normalized Fit Score
1	1VBI	Dehydrogenase	2.935	0.9784
2	5E2E	NONE	2.666	0.8886
3	3BHY	Death-associated protein kinase 3	2.999	0.7498
4	1D06	Sensor protein fixL	2.995	0.7488
5	3B8C	ATPase 2, plasma membrane-type	2.995	0.7487
6	2CR7	Paired amphipathic helix protein Sin3b	2.994	0.7484
7	2DQN	Glutamyl-tRNA(Gln) amidotransferase subunit A	2.994	0.7484
8	2CST	Aspartate aminotransferase, cytoplasmic	2.979	0.7448
9	2I15	Uncharacterized protein MG296 homolog	2.979	0.7447
10	1LJ0	Cytochrome b5 type B	2.973	0.7432
11	2OWI	Regulator of G-protein signaling 18	2.966	0.7416
12	2NRO	Molybdopterin biosynthesis protein moeA	2.947	0.7368
13	1J3N	Transferase	2.939	0.7348
14	3HKI	Prothrombin	2.939	0.7347
15	2VRE	Delta(3,5)-Delta(2,4)-dienoyl-CoA isomerase, mitochondrial	2.937	0.7343
16	2JWE	Tight junction protein ZO-1	2.914	0.7284
17	2QFD	Probable ATP-dependent RNA helicase DDX58	2.888	0.722
18	3DRA	Protein farnesyltransferase/geranylgeranyltransferase type-1 subunit alpha	2.883	0.7208
19	2GNX	UPF0536 protein C12orf66 homolog	2.865	0.7162
20	1YED	Ig gamma-1 chain C region secreted form	2.81	0.7026
21	2GQF	Uncharacterized protein HI0933	2.807	0.7018
22	1B0N	HTH-type transcriptional regulator sinR	2.803	0.7007
23	2IBJ	Cytochrome b5	2.795	0.6986
24	1GMG	Regulatory protein rop	2.774	0.6935
25	1UYR	Acetyl-CoA carboxylase	2.76	0.69
26	1TAF	Transcription initiation factor TFIID subunit 9	2.685	0.6713
27	1FFT	Ubiquinol oxidase subunit 1	2.587	0.6466
28	2AQT	Superoxide dismutase [Cu-Zn]	2.527	0.6319

29	2JZ6	50S ribosomal protein L28	2.483	0.6208
30	1GK9	Penicillin G acylase	2.428	0.607
31	1ES8	Type II restriction enzyme BglII	2.407	0.6018
32	1VF5	Cytochrome b6	2.999	0.5999
33	1SNL	Nucleobindin-1	2.999	0.5999
34	1GH7	Cytokine receptor common subunit beta	2.999	0.5998
35	1UFI	Major centromere autoantigen B	2.998	0.5995
36	2F8N	Histone H3.2	2.993	0.5985
37	2BTY	Acetylglutamate kinase	2.992	0.5984
38	1RKS	Ribokinase	2.991	0.5983
39	1IVH	Isovaleryl-CoA dehydrogenase, mitochondrial	2.99	0.598
40	1GGZ	Calmodulin-like protein 3	2.988	0.5977
41	1NA6	Type-2 restriction enzyme EcoRII	2.988	0.5976
42	2AY0	Bifunctional protein putA	2.988	0.5976
43	3G9K	Capsule biosynthesis protein capD	2.988	0.5976
44	1GGT	Coagulation factor XIII A chain	2.985	0.5969
45	1W8I	Uncharacterized protein AF_1683	2.984	0.5968
46	1ZXK	Cadherin-8	2.98	0.5959
47	1VRN	Photosynthetic reaction center cytochrome c subunit	2.979	0.5958
48	1E51	Delta-aminolevulinic acid dehydratase	2.979	0.5957
49	2AYN	Ubiquitin carboxyl-terminal hydrolase 14	2.978	0.5956
50	2VWT	2-keto-3-deoxy-L-rhamnonate aldolase	2.976	0.5953

Table S11. PharmMapper result for molecule **13a**.

	PDB ID	Target Name	Fit Score	Normalized Fit Score
1	2FH0	Uncharacterized protein YMR074C	2.949	0.983
2	4LUR	NONE	2.946	0.9821
3	1M4Y	ATP-dependent protease hslV	3.282	0.8204
4	1MJE	26S proteasome complex subunit DSS1	3.013	0.7531
5	2DU7	O-phosphoseryl-tRNA(Cys) synthetase	2.975	0.7438
6	2CST	Aspartate aminotransferase, cytoplasmic	2.973	0.7431
7	1JVW	Macrophage infectivity potentiator	2.97	0.7425
8	1RF8	Eukaryotic translation initiation factor 4E	2.966	0.7416
9	2QG3	UPF0130 protein AF_2059	2.962	0.7405
10	3EMJ	Inorganic pyrophosphatase	2.956	0.7391
11	2JZ6	50S ribosomal protein L28	2.951	0.7377
12	2VRE	Delta(3,5)-Delta(2,4)-dienoyl-CoA isomerase, mitochondrial	2.946	0.7366
13	2WA0	Melanoma-associated antigen 4	2.935	0.7338
14	1C0L	D-amino-acid oxidase	2.93	0.7325
15	1BUV	Matrix metalloproteinase-14	2.927	0.7319
16	2E02	Cysteine proteinase 1, mitochondrial	2.924	0.731
17	1YED	Ig gamma-1 chain C region secreted form	2.916	0.729

18	2NVB	NADP-dependent alcohol dehydrogenase	2.908	0.727
19	2YXR	Preprotein translocase subunit secY	2.907	0.7267
20	3FFV	Protein syd	2.903	0.7258
21	2EX5	DNA endonuclease I-CeuI	2.901	0.7254
22	2GFP	Multidrug resistance protein D	2.898	0.7245
23	1FRV	Periplasmic [NiFe] hydrogenase small subunit	2.869	0.7173
24	2PO0	Probable exosome complex exonuclease 1	2.866	0.7166
25	2VLD	UPF0286 protein PYRAB01260	2.866	0.7164
26	3DRA	Protein farnesyltransferase/geranylgeranyltransferase type-1 subunit alpha	2.859	0.7147
27	1VBG	Pyruvate, phosphate dikinase 1, chloroplastic	2.852	0.7131
28	3D64	Adenosylhomocysteinase	2.85	0.7126
29	1QRJ	Gag-Pro-Pol polyprotein	2.847	0.7118
30	3FQD	5-3 exoribonuclease 2	3.313	0.6626
31	3D3L	Arachidonate 12-lipoxygenase, 12S-type	3.168	0.6335
32	1X65	Cold shock domain-containing protein E1	3.78	0.6299
33	2WBI	Acyl-CoA dehydrogenase family member 11	3.055	0.6109
34	1IGR	Insulin-like growth factor 1 receptor	3.011	0.6022
35	2GJX	Beta-hexosaminidase subunit alpha	3	0.5999
36	2ES0	Regulator of G-protein signaling 6	2.992	0.5985
37	1XES	Dihydropinosylvin synthase	2.991	0.5982
38	2RM4	Enhancer of mRNA-decapping protein 3	2.99	0.5979
39	2H63	Biliverdin reductase A	2.986	0.5971
40	1L0V	Fumarate reductase flavoprotein subunit	2.983	0.5967
41	2E55	Uracil phosphoribosyltransferase	3.579	0.5965
42	1VF5	Cytochrome b6	2.982	0.5964
43	2R46	Aerobic glycerol-3-phosphate dehydrogenase	2.98	0.596
44	1UW4	Regulator of nonsense transcripts 3B	2.974	0.5947
45	1IVX	Phenylethylamine oxidase	2.96	0.5921
46	1OVT	Ovotransferrin	2.951	0.5902
47	1PUJ	Ribosome biogenesis GTPase A	3.537	0.5895
48	2AY0	Bifunctional protein putA	2.946	0.5893
49	2HJS	USG-1 protein homolog	2.941	0.5883
50	1JEY	ATP-dependent DNA helicase 2 subunit 1	2.938	0.5876

Table S12. PharmMapper result for molecule **15a**.

	PDB ID	Target Name	Fit Score	Normalized Fit Score
1	1M4Y	ATP-dependent protease hslV	3.264	0.8159
2	1RZO	Agglutinin	3.158	0.7896
3	2XBU	NONE	3.084	0.771
4	3FFV	Protein syd	2.989	0.7473

5	2JZ6	50S ribosomal protein L28	2.973	0.7432
6	2AQT	Superoxide dismutase [Cu-Zn]	2.955	0.7386
7	2VRE	Delta(3,5)-Delta(2,4)-dienoyl-CoA isomerase, mitochondrial	2.907	0.7268
8	2CST	Aspartate aminotransferase, cytoplasmic	2.888	0.722
9	2QEQ	Genome polyprotein	2.865	0.7163
10	2B6E	Putative esterase HI1161	2.861	0.7153
11	3DRA	Protein farnesyltransferase/geranylgeranyltransferase type-1 subunit alpha	2.861	0.7152
12	1UYR	Acetyl-CoA carboxylase	2.852	0.713
13	2VLD	UPF0286 protein PYRAB01260	2.837	0.7092
14	1R6U	Tryptophanyl-tRNA synthetase, cytoplasmic	2.827	0.7068
15	1PP9	Cytochrome b-c1 complex subunit 1, mitochondrial	2.821	0.7052
16	2RFA	Transient receptor potential cation channel subfamily V member 6	2.82	0.705
17	2E0G	Chromosomal replication initiator protein dnaA	2.81	0.7024
18	1KCG	NKG2D ligand 3	2.773	0.6933
19	1QZ9	Kynureninase	2.773	0.6931
20	1FRV	Periplasmic [NiFe] hydrogenase small subunit	2.77	0.6924
21	3DXR	Mitochondrial import inner membrane translocase subunit TIM9	2.763	0.6908
22	2DBA	Protein unc-45 homolog A	2.756	0.689
23	2PUJ	2-hydroxy-6-oxo-6-phenylhexa-2,4-dienoate hydrolase	2.753	0.6882
24	2W9S	Dihydrofolate reductase type 1 from Tn4003	2.742	0.6855
25	1LSH	Vitellogenin	2.73	0.6825
26	1QRJ	Gag-Pro-Pol polyprotein	2.722	0.6804
27	1W9C	Exportin-1	2.716	0.679
28	1F3T	Ornithine decarboxylase	2.711	0.6776
29	1SYO	Cation-independent mannose-6-phosphate receptor	2.691	0.6728
30	2D7R	Polypeptide N-acetylgalactosaminyltransferase 10	2.679	0.6697
31	2DQN	Glutamyl-tRNA(Gln) amidotransferase subunit A	3.34	0.668
32	2DYQ	Amyloid beta A4 precursor protein-binding family B member 3	2.67	0.6675
33	1EE8	Formamidopyrimidine-DNA glycosylase	2.648	0.6621
34	1FPP	Protein farnesyltransferase subunit beta	3.298	0.6596
35	1P9B	Adenylosuccinate synthetase	2.636	0.6589
36	1Q3O	SH3 and multiple ankyrin repeat domains protein 1	2.634	0.6586
37	1J4A	D-lactate dehydrogenase	3.224	0.6448
38	3E7W	D-alanine--poly(phosphoribitol) ligase subunit 1	3.798	0.633
39	1IGR	Insulin-like growth factor 1 receptor	3.133	0.6267
40	2E55	Uracil phosphoribosyltransferase	3.565	0.5942
41	2H63	Biliverdin reductase A	2.963	0.5926
42	1YPF	GMP reductase	2.95	0.5901
43	2D3L	Glucan 1,4-alpha-maltohexaosidase	2.941	0.5883
44	1T9K	Methylthioribose-1-phosphate isomerase	2.922	0.5845

45	2HJS	USG-1 protein homolog	2.911	0.5822
46	2G18	Phycocyanobili	2.904	0.5809
47	1Y1U	Signal transducer and activator of transcription 5A	2.895	0.579
48	1TM0	Uncharacterized protein BMEI1586	2.893	0.5786
49	1C8S	Bacteriorhodopsin	2.89	0.578
50	2P1Q	SKP1-like protein 1A	2.874	0.5748

Table S13. PharmMapper result for molecule **15f**.

	PDB ID	Target Name	Fit Score	Normalized Fit Score
1	1LFG	Lactotransferrin	2.97	0.9899
2	2FH0	Uncharacterized protein YMR074C	2.926	0.9754
3	2VVJ	NONE	3.308	0.8269
4	1M4Y	ATP-dependent protease hslV	3.285	0.8213
5	1RZO	Agglutinin	3.207	0.8016
6	1MJE	26S proteasome complex subunit DSS1	3.032	0.7579
7	2VEQ	Centromere DNA-binding protein complex CBF3 subunit B	2.991	0.7478
8	3DRA	Protein farnesyltransferase/geranylgeranyltransferase type-1 subunit alpha	2.975	0.7437
9	3FFV	Protein syd	2.974	0.7434
10	2AQT	Superoxide dismutase [Cu-Zn]	2.954	0.7384
11	2VRE	Delta(3,5)-Delta(2,4)-dienoyl-CoA isomerase, mitochondrial	2.954	0.7384
12	2NVB	NADP-dependent alcohol dehydrogenase	2.952	0.7379
13	2GQF	Uncharacterized protein HI0933	2.943	0.7358
14	3DXR	Mitochondrial import inner membrane translocase subunit TIM9	2.942	0.7356
15	2QEQ	Genome polyprotein	2.939	0.7348
16	2JZ6	50S ribosomal protein L28	2.938	0.7346
17	1LJ0	Cytochrome b5 type B	2.917	0.7293
18	2CST	Aspartate aminotransferase, cytoplasmic	2.912	0.728
19	2PUJ	2-hydroxy-6-oxo-6-phenylhexa-2,4-dienoate hydrolase	2.904	0.726
20	2GNX	UPF0536 protein C12orf66 homolog	2.9	0.7249
21	2I15	Uncharacterized protein MG296 homolog	2.886	0.7214
22	3FQD	5-3 exoribonuclease 2	3.341	0.6682
23	2DQN	Glutamyl-tRNA(Gln) amidotransferase subunit A	3.11	0.622
24	1J4A	D-lactate dehydrogenase	3.109	0.6219
25	1FPP	Protein farnesyltransferase subunit beta	3.103	0.6206
26	1D5M	HLA class II histocompatibility antigen, DRB1-4 beta chain	3.049	0.6099
27	1IGR	Insulin-like growth factor 1 receptor	3.002	0.6003
28	1L0V	Fumarate reductase flavoprotein subunit	2.998	0.5995
29	1OVT	Ovotransferrin	2.997	0.5995

30	1XES	Dihydropinosylvin synthase	2.992	0.5983
31	2E55	Uracil phosphoribosyltransferase	3.589	0.5981
32	1B0A	Bifunctional protein folD	2.986	0.5972
33	3DF0	Calpain-2 catalytic subunit	2.986	0.5971
34	1W8I	Uncharacterized protein AF_1683	2.98	0.596
35	2RNX	Histone acetyltransferase KAT2B	2.974	0.5948
36	3EXA	tRNA Delta(2)-isopentenylpyrophosphate transferase	2.974	0.5948
37	1IVX	Phenylethylamine oxidase	2.968	0.5935
38	1EXV	Glycogen phosphorylase, liver form	2.965	0.593
39	2AY0	Bifunctional protein putA	2.964	0.5928
40	1YVG	Tetanus toxin	2.961	0.5922
41	2H63	Biliverdin reductase A	2.952	0.5904
42	2P1Q	SKP1-like protein 1A	2.951	0.5901
43	2F8N	Histone H3.2	2.948	0.5897
44	1J3I	Bifunctional dihydrofolate reductase-thymidylate synthase	2.948	0.5896
45	3D47	L-rhamnonate dehydratase	3.529	0.5882
46	1C8S	Bacteriorhodopsin	2.927	0.5854
47	2D3L	Glucan 1,4-alpha-maltohexaosidase	2.926	0.5853
48	2H60	Probable global transcription activator SNF2L4	2.919	0.5839
49	1ZXK	Cadherin-8	2.917	0.5833
50	2GP6	3-oxoacyl-[acyl-carrier-protein] synthase 2	2.914	0.5827

Table S14. PharmMapper result for molecule **17a**.

	PDB ID	Target Name	Fit Score	Normalized Fit Score
1	1UJV	Membrane-associated guanylate kinase, WW and PDZ domain-containing protein 2	2.996	0.7489
2	3RFW	NONE	2.987	0.7467
3	1P35	Early 35 kDa protein	2.97	0.7426
4	1U2E	2-hydroxy-6-oxononadienedioate/2-hydroxy-6-oxononatrienedioate hydrolase	2.963	0.7409
5	1RZU	Glycogen synthase 1	2.959	0.7398
6	3B3D	Uncharacterized oxidoreductase ytbE	2.959	0.7397
7	1JL2	Ribonuclease HI	2.956	0.7391
8	1YAV	Uncharacterized protein ykuL	2.938	0.7345
9	2AQT	Superoxide dismutase [Cu-Zn]	2.921	0.7301
10	2CWX	Ribulose bisphosphate carboxylase	2.92	0.7301
11	2VEQ	Centromere DNA-binding protein complex CBF3 subunit B	2.918	0.7295
12	2CUE	Paired box protein Pax-6	2.907	0.7268
13	2NVB	NADP-dependent alcohol dehydrogenase	2.907	0.7267
14	2CST	Aspartate aminotransferase, cytoplasmic	2.896	0.724
15	1U94	Protein recA	2.869	0.7172
16	1RZO	Agglutinin	2.864	0.716
17	3FQD	5-3 exoribonuclease 2	3.407	0.6814

18	1QOX	Beta-glucosidase	3.274	0.6548
19	1XJV	Protection of telomeres protein 1	3.242	0.6484
20	1WIN	Flotillin-2	3.237	0.6474
21	1BUC	Acyl-CoA dehydrogenase, short-chain specific	3.196	0.6392
22	1OGY	Periplasmic nitrate reductase	3.136	0.6273
23	3DLJ	Beta-Ala-His dipeptidase	3.127	0.6253
24	2QVV	Fructose-1,6-bisphosphatase 1	4.31	0.6158
25	1S40	Cell division control protein 13	3.682	0.6136
26	2VIG	Short-chain specific acyl-CoA dehydrogenase, mitochondrial	3.067	0.6134
27	1J3I	Bifunctional dihydrofolate reductase-thymidylate synthase	3	0.6
28	1U08	Aminotransferase ybdL	3	0.6
29	2RNX	Histone acetyltransferase KAT2B	3	0.6
30	1EXV	Glycogen phosphorylase, liver form	2.995	0.599
31	2YW W	Aspartate carbamoyltransferase regulatory chain	2.988	0.5975
32	1Q4R	Probable protein Pop3	2.977	0.5955
33	2G18	Phycocyanobili	2.972	0.5944
34	2Z6E	Disheveled-associated activator of morphogenesis 1	2.964	0.5927
35	2GP6	3-oxoacyl-[acyl-carrier-protein] synthase 2	2.954	0.5908
36	1R0U	Uncharacterized beta-barrel protein ywiB	2.95	0.5899
37	1Y1U	Signal transducer and activator of transcription 5A	2.944	0.5888
38	3EXA	tRNA Delta(2)-isopentenylpyrophosphate transferase	2.943	0.5886
39	2AY0	Bifunctional protein putA	2.94	0.5881
40	1C9B	Transcription initiation factor IIB	2.936	0.5872
41	1I3R	H-2 class II histocompatibility antigen, E-K alpha chain	2.929	0.5857
42	2R0N	Glutaryl-CoA dehydrogenase, mitochondrial	2.925	0.585
43	2F8N	Histone H3.2	2.917	0.5834
44	2BTV	Core protein VP3	2.899	0.5798
45	2FY4	Choline O-acetyltransferase	2.891	0.5781
46	2BTY	Acetylglutamate kinase	2.889	0.5778
47	1VRN	Photosynthetic reaction center cytochrome c subunit	2.888	0.5777
48	1B0A	Bifunctional protein folD	2.887	0.5774
49	3GVI	Malate dehydrogenase	2.883	0.5767
50	2EJE	General transcription factor II-I	3.455	0.5758

Table S15. PharmMapper result for molecule **20**.

	PDB ID	Target Name	Fit Score	Normalized Fit Score
1	2FH0	Uncharacterized protein YMR074C	2.99	0.9968
2	3DG3	NONE	2.99	0.9968
3	1M4Y	ATP-dependent protease hslV	3.271	0.8178
4	1RZO	Agglutinin	3.096	0.774
5	2FM8	Surface presentation of antigens protein spaK	2.996	0.749
6	1R1T	Transcriptional repressor smtB	2.993	0.7483
7	1TAF	Transcription initiation factor TFIID subunit 9	2.987	0.7469
8	2JZ6	50S ribosomal protein L28	2.985	0.7463

9	2CST	Aspartate aminotransferase, cytoplasmic	2.983	0.7457
10	2IBJ	Cytochrome b5	2.981	0.7453
11	1JFI	Dr1-associated corepressor	2.98	0.7451
12	1WVC	Glucose-1-phosphate cytidylyltransferase	2.979	0.7447
13	1QRJ	Gag-Pro-Pol polyprotein	2.977	0.7443
14	1F81	CREB-binding protein	2.977	0.7442
15	3CA8	Protein ydcF	2.976	0.7441
16	1YED	Ig gamma-1 chain C region secreted form	2.976	0.7441
17	1JVW	Macrophage infectivity potentiator	2.973	0.7433
18	3DRA	Protein farnesyltransferase/geranylgeranyltransferase type-1 subunit alpha	2.973	0.7432
19	1W1G	3-phosphoinositide-dependent protein kinase 1	2.969	0.7422
20	3FFV	Protein syd	2.969	0.7422
21	1BG5	Glutathione S-transferase class-mu 26 kDa isozyme	2.969	0.7422
22	2QFD	Probable ATP-dependent RNA helicase DDX58	2.969	0.7421
23	1LK3	Interleukin-10	2.968	0.742
24	2OWI	Regulator of G-protein signaling 18	2.965	0.7413
25	1FRV	Periplasmic [NiFe] hydrogenase small subunit	2.965	0.7412
26	2NRO	Molybdopterin biosynthesis protein moeA	2.964	0.7409
27	2RGV	Peroxide operon regulator	2.964	0.7409
28	1IOD	Agkisacutacin subunit A	2.962	0.7406
29	2OPX	Lactaldehyde dehydrogenase	2.955	0.7387
30	1GMG	Regulatory protein rop	2.953	0.7384
31	1ZZG	Glucose-6-phosphate isomerase	2.953	0.7384
32	3EMJ	Inorganic pyrophosphatase	2.953	0.7381
33	2VRE	Delta(3,5)-Delta(2,4)-dienoyl-CoA isomerase, mitochondrial	2.952	0.7379
34	3FQD	5-3 exoribonuclease 2	3.512	0.7024
35	3HBF	Flavonoid 3-O-glucosyltransferase	3.307	0.6614
36	1I4H	Enterotoxin type A	3.293	0.6587
37	2Z6E	Disheveled-associated activator of morphogenesis 1	3.234	0.6468
38	1Q4R	Probable protein Pop3	3.229	0.6459
39	2E2E	Formate-dependent nitrite reductase complex subunit nrfG	3.225	0.645
40	1D5M	HLA class II histocompatibility antigen, DRB1-4 beta chain	3.195	0.6389
41	2DQN	Glutamyl-tRNA(Gln) amidotransferase subunit A	3.192	0.6385
42	1TXO	PP2C-family Ser/Thr phosphatase	3.164	0.6327
43	2IWQ	Multiple PDZ domain protein	3.157	0.6314
44	1WJI	Tudor domain-containing protein 3	3.767	0.6279
45	1R44	D-alanyl-D-alanine dipeptidase	3.117	0.6234
46	1LYL	Lysyl-tRNA synthetase, heat inducible	3.112	0.6225
47	1IGR	Insulin-like growth factor 1 receptor	3.111	0.6221
48	1J4A	D-lactate dehydrogenase	3.089	0.6177
49	1X65	Cold shock domain-containing protein E1	3.694	0.6156
50	3E7W	D-alanine--poly(phosphoribitol) ligase subunit 1	3.658	0.6096

Table S16. PharmMapper result for molecule **21a**.

	PDB ID	Target Name	Fit Score	Normalized Fit Score
1	1U02	NONE	2.981	0.9938
2	2UXW	Very long-chain specific acyl-CoA dehydrogenase, mitochondrial	2.981	0.9937
3	2CT7	RING finger protein 31	2.969	0.9897
4	1ROZ	Deoxyhypusine synthase	2.959	0.9863
5	1NI2	Ezrin	2.95	0.9833
6	2I15	Uncharacterized protein MG296 homolog	2.942	0.9807
7	2GZA	Type IV secretion system protein virB11	2.941	0.9803
8	2CTK	Vigilin	2.935	0.9782
9	1EVY	Glycerol-3-phosphate dehydrogenase [NAD+], glycosomal	2.934	0.9779
10	1LJ0	Cytochrome b5 type B	2.998	0.7494
11	2C9O	RuvB-like 1	2.994	0.7486
12	1P35	Early 35 kDa protein	2.988	0.747
13	1TYB	Tyrosyl-tRNA synthetase	2.984	0.7461
14	1VB6	Heme-regulated cyclic AMP phosphodiesterase	2.98	0.745
15	2BK3	Amine oxidase [flavin-containing] B	3.719	0.7438
16	2NVB	NADP-dependent alcohol dehydrogenase	2.974	0.7436
17	2INR	DNA topoisomerase 4 subunit A	2.973	0.7432
18	2JHQ	Uracil-DNA glycosylase	2.972	0.743
19	3DWL	Actin-related protein 3	2.968	0.742
20	2CR7	Paired amphipathic helix protein Sin3b	2.967	0.7417
21	2QEQ	Genome polyprotein	2.965	0.7412
22	1XKE	E3 SUMO-protein ligase RanBP2	2.964	0.741
23	1YED	Ig gamma-1 chain C region secreted form	2.963	0.7408
24	1R1T	Transcriptional repressor smtB	2.96	0.7399
25	2IBJ	Cytochrome b5	2.957	0.7392
26	2E02	Cysteine proteinase 1, mitochondrial	2.957	0.7392
27	2VEQ	Centromere DNA-binding protein complex CBF3 subunit B	2.957	0.7392
28	1U2E	2-hydroxy-6-oxononadienedioate/2-hydroxy-6-oxononatrienedioate hydrolase	2.957	0.7391
29	1IEB	H-2 class II histocompatibility antigen, E-D alpha chain	2.954	0.7386
30	1ZR4	Transposon gamma-delta resolvase	2.954	0.7385
31	1OSA	Calmodulin	2.954	0.7384
32	2HGK	Uncharacterized protein yqcC	2.953	0.7383
33	1FFT	Ubiquinol oxidase subunit 1	2.95	0.7375
34	2IX4	3-oxoacyl-[acyl-carrier-protein] synthase, mitochondrial	2.95	0.7375
35	3CRC	Protein mazG	2.947	0.7368
36	1IG3	Thiamin pyrophosphokinase 1	2.944	0.7361
37	1ZRT	Ubiquinol-cytochrome c reductase iron-sulfur subunit	2.944	0.736
38	2EW0	UPF0301 protein ACIAD0353	2.942	0.7355
39	1KU5	Archaeal histone A	2.941	0.7354
40	2GQF	Uncharacterized protein HI0933	2.941	0.7352
41	2CV8	tRNA-splicing endonuclease	2.94	0.735

42	3EMJ	Inorganic pyrophosphatase	2.94	0.735
43	2CW X	Ribulose bisphosphate carboxylase	2.94	0.7349
44	3D64	Adenosylhomocysteinase	2.938	0.7344
45	1UD9	DNA polymerase sliding clamp A	2.936	0.734
46	2GFP	Multidrug resistance protein D	2.936	0.734
47	3CT4	PTS-dependent dihydroxyacetone kinase, dihydroxyacetone-binding subunit dhaK	2.935	0.7338
48	1JFI	Dr1-associated corepressor	2.934	0.7335
49	1ES8	Type II restriction enzyme BglII	2.934	0.7334
50	1UYR	Acetyl-CoA carboxylase	2.93	0.7326

Table S17. PharmMapper result for molecule **21d**.

	PDB ID	Target Name	Fit Score	Normalized Fit Score
1	1IVH	Isovaleryl-CoA dehydrogenase, mitochondrial	3.807	0.7615
2	2I15	Uncharacterized protein MG296 homolog	2.999	0.7498
3	3RFW	NONE	2.996	0.749
4	1D06	Sensor protein fixL	2.996	0.749
5	2VEQ	Centromere DNA-binding protein complex CBF3 subunit B	2.994	0.7486
6	2AQT	Superoxide dismutase [Cu-Zn]	2.993	0.7483
7	2GQF	Uncharacterized protein HI0933	2.993	0.7482
8	2CR7	Paired amphipathic helix protein Sin3b	2.992	0.7481
9	3B8C	ATPase 2, plasma membrane-type	2.989	0.7474
10	1LJ0	Cytochrome b5 type B	2.987	0.7468
11	2NVB	NADP-dependent alcohol dehydrogenase	2.986	0.7465
12	1QZ9	Kynureninase	2.984	0.746
13	1P35	Early 35 kDa protein	2.98	0.7451
14	1P9O	Phosphopantothenate--cysteine ligase	2.979	0.7448
15	3BHY	Death-associated protein kinase 3	2.975	0.7439
16	2QEQ	Genome polyprotein	2.97	0.7426
17	2CWX	Ribulose bisphosphate carboxylase	2.967	0.7418
18	2DQN	Glutamyl-tRNA(Gln) amidotransferase subunit A	2.966	0.7414
19	2CST	Aspartate aminotransferase, cytoplasmic	2.963	0.7408
20	3HKI	Prothrombin	2.962	0.7405
21	3FMO	Nuclear pore complex protein Nup214	2.959	0.7397
22	1JL2	Ribonuclease HI	2.957	0.7391
23	1YQT	RNase l inhibitor	2.954	0.7385
24	3CPR	Dihydrodipicolinate synthase	2.954	0.7384
25	2NRO	Molybdopterin biosynthesis protein moeA	2.945	0.7363
26	2FJU	Ras-related C3 botulinum toxin substrate 1	2.932	0.733
27	1W63	AP-1 complex subunit gamma-1	2.932	0.7329
28	2OQE	Peroxisomal primary amine oxidase	2.927	0.7318
29	2PKP	Homoaconitase small subunit	2.926	0.7314
30	2OWI	Regulator of G-protein signaling 18	2.925	0.7312
31	1JQK	Carbon monoxide dehydrogenase	2.924	0.7311

32	3FFV	Protein syd	2.92	0.7299
33	1KCG	NKG2D ligand 3	2.919	0.7297
34	2VRE	Delta(3,5)-Delta(2,4)-dienoyl-CoA isomerase, mitochondrial	2.918	0.7295
35	1V2Y	U11/U12 small nuclear ribonucleoprotein 25 kDa protein	2.917	0.7292
36	1UYR	Acetyl-CoA carboxylase	2.916	0.7289
37	3CRC	Protein mazG	2.908	0.7269
38	2KA5	Putative anti-sigma factor antagonist TM_1081	3.829	0.6382
39	3BID	UPF0339 protein NMA1193/NMA1859	3.683	0.6139
40	2RNX	Histone acetyltransferase KAT2B	3.06	0.6121
41	1E51	Delta-aminolevulinic acid dehydratase	3	0.6
42	1SNL	Nucleobindin-1	2.999	0.5997
43	1J3I	Bifunctional dihydrofolate reductase-thymidylate synthase	2.998	0.5996
44	1GH7	Cytokine receptor common subunit beta	2.997	0.5995
45	1EXV	Glycogen phosphorylase, liver form	2.996	0.5993
46	2YWW	Aspartate carbamoyltransferase regulatory chain	2.985	0.597
47	1DK8	Axin-1	2.985	0.5969
48	3DF0	Calpain-2 catalytic subunit	2.984	0.5968
49	1XES	Dihydropinosylvin synthase	2.984	0.5968
50	1L0V	Fumarate reductase flavoprotein subunit	2.981	0.5962

Table S18. Pharmacophore screening results.

N molecules [a]	PDB ID	Target Name
6	2CST	Aspartate aminotransferase, cytoplasmic
5	2I15	Uncharacterized protein MG296 homolog
4	1D06	Sensor protein fixL
4	1M4Y	ATP-dependent protease hslV
4	1VBI	Dehydrogenase
4	2AQT	Superoxide dismutase [Cu-Zn]
3	1LJ0	Cytochrome b5 type B
3	1RZO	Agglutinin
3	2FH0	Uncharacterized protein YMR074C
3	2GWF	Ubiquitin carboxyl-terminal hydrolase 8
3	2GZA	Type IV secretion system protein virB11
3	3B8C	ATPase 2, plasma membrane-type
2	1DK8	Axin-1
2	1EVY	Glycerol-3-phosphate dehydrogenase [NAD+], glycosomal
2	1LFG	Lactotransferrin
2	1MJE	26S proteasome complex subunit DSS1
2	1R1T	Transcriptional repressor smtB
2	1RF8	Eukaryotic translation initiation factor 4E
2	1ROZ	Deoxyhypusine synthase

2	1U2E	2-hydroxy-6-oxononadienedioate/2-hydroxy-6-oxononatrienedioate hydrolase
2	1UJV	Membrane-associated guanylate kinase, WW and PDZ domain-containing protein 2
2	1UKK	Osmotically inducible protein C
2	1XES	Dihydropinosylvin synthase
2	2CR7	Paired amphipathic helix protein Sin3b
2	2CT7	RING finger protein 31
2	2DQN	Glutamyl-tRNA(Gln) amidotransferase subunit A
2	2GQF	Uncharacterized protein HI0933
2	2JZ6	50S ribosomal protein L28
2	2OWI	Regulator of G-protein signaling 18
2	2UXW	Very long-chain specific acyl-CoA dehydrogenase, mitochondrial
2	2VEQ	Centromere DNA-binding protein complex CBF3 subunit B
2	2VRE	Delta(3,5)-Delta(2,4)-dienoyl-CoA isomerase, mitochondrial
2	3B3D	Uncharacterized oxidoreductase ytbE
2	3BHY	Death-associated protein kinase 3
2	3DRA	Protein farnesyltransferase/geranylgeranyltransferase type-1 subunit alpha
2	3FFV	Protein syd

[a] number of molecules that have the indicated target among the best 10 proteins targets identified by the pharmacophore mapping analysis.

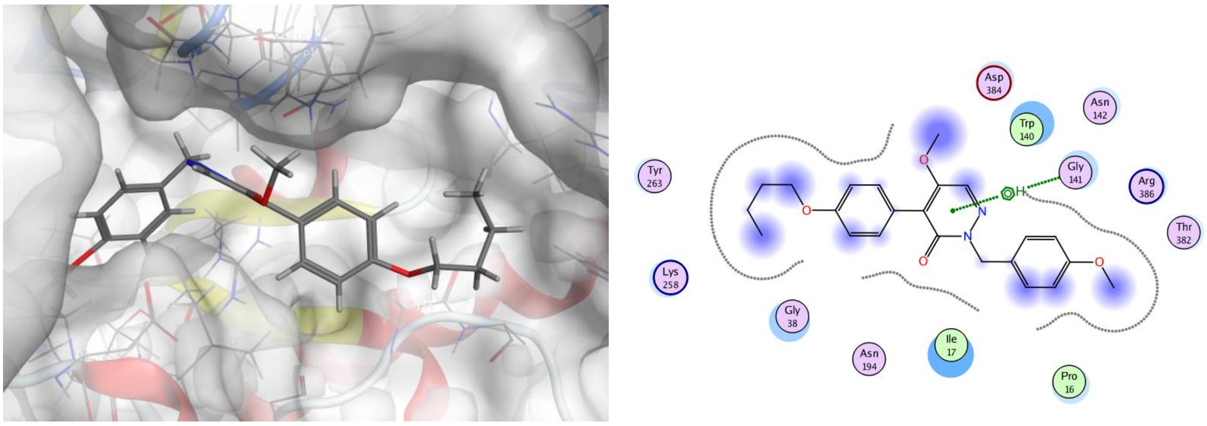

Figure S1. Docking pose of compound **1a**.

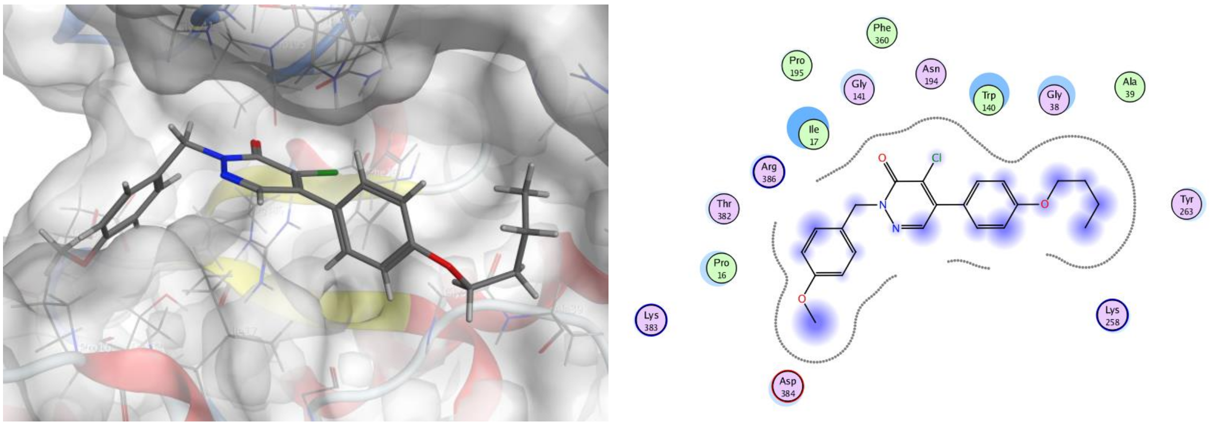

Figure S2. Docking pose of compound **2a**.

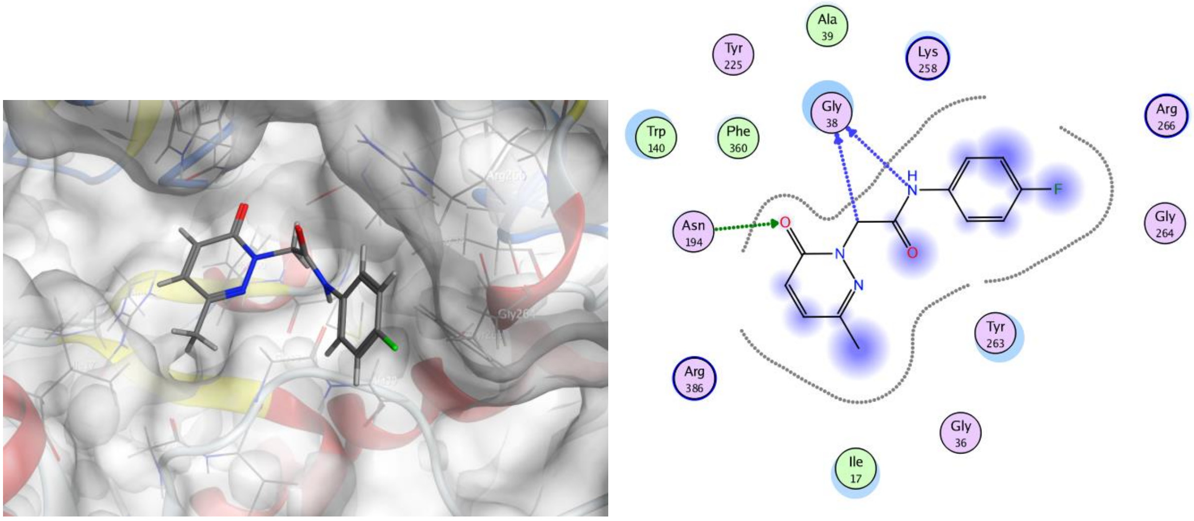

Figure S3. Docking pose of compound **3a**.

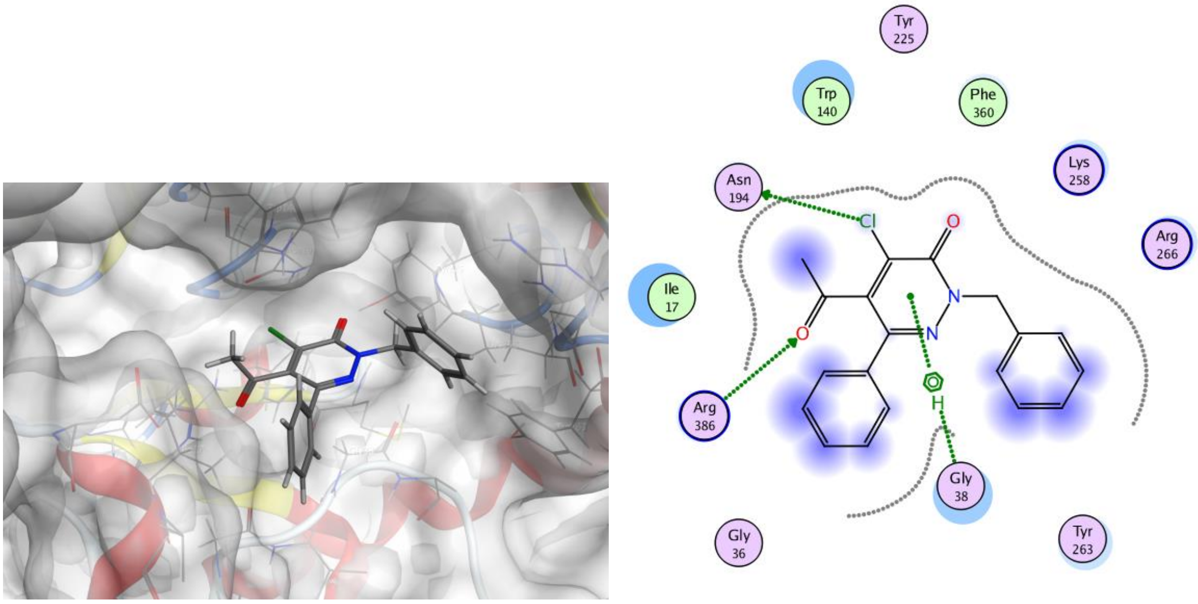

Figure S4. Docking pose of compound **5a.**

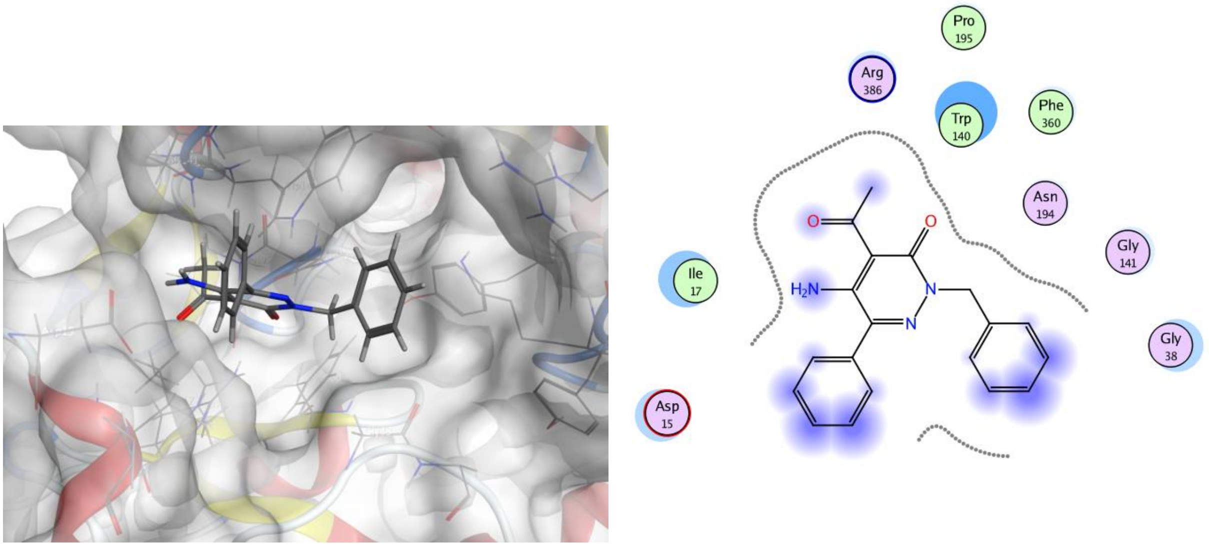

Figure S5. Docking pose of compound **7**.

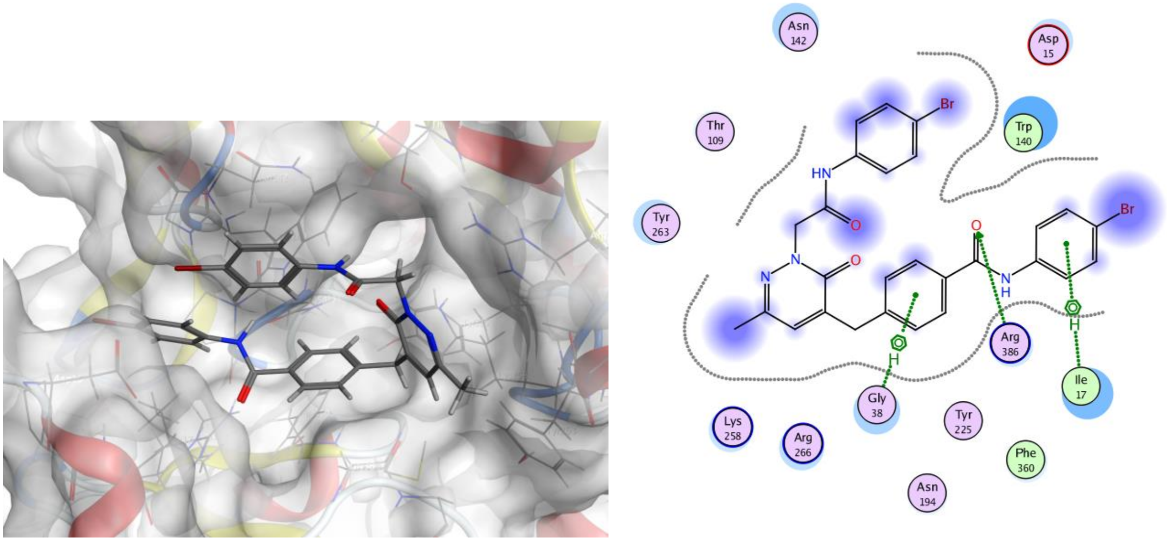

Figure S6. Docking pose of compound **8**.

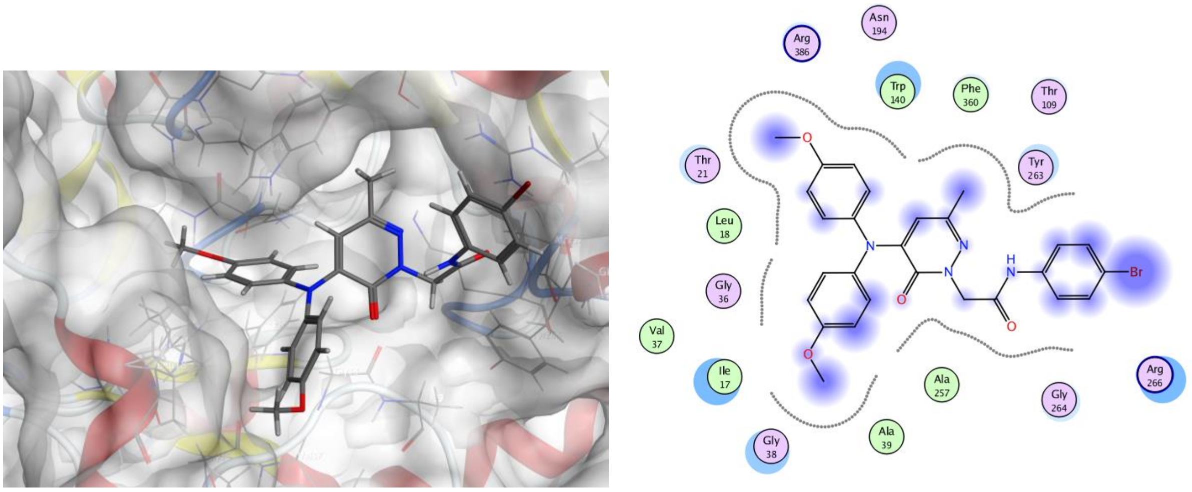

Figure S7. Docking pose of compound **9**.

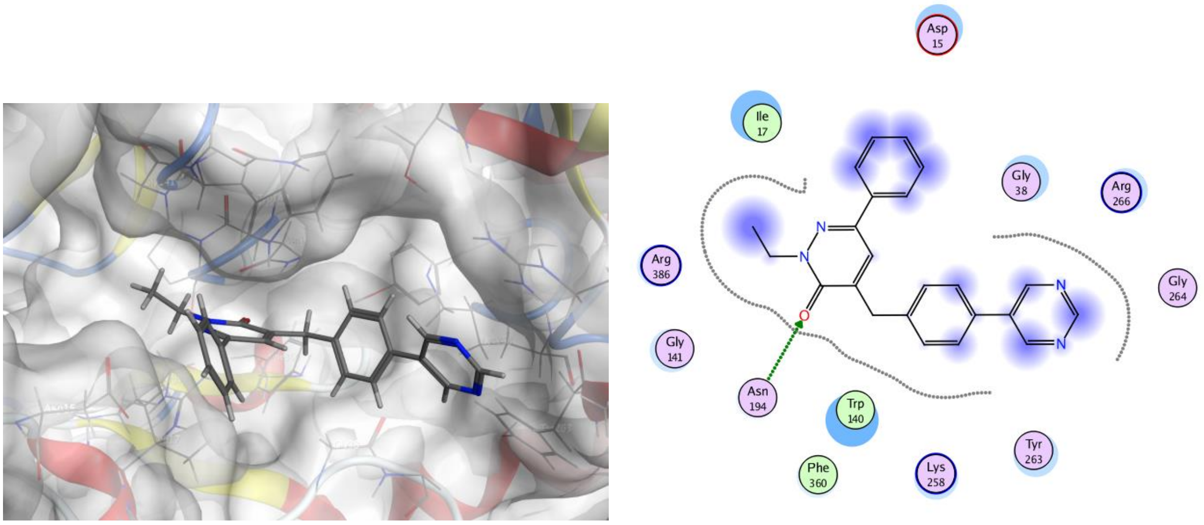

Figure S8. Docking pose of compound **10b**.

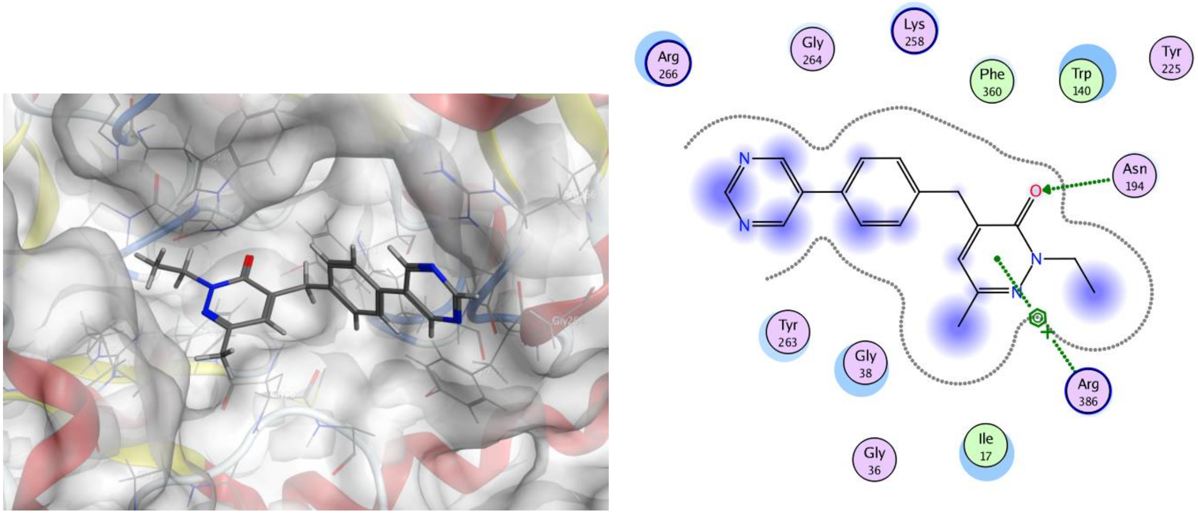

Figure S9. Docking pose of compound **10g**.

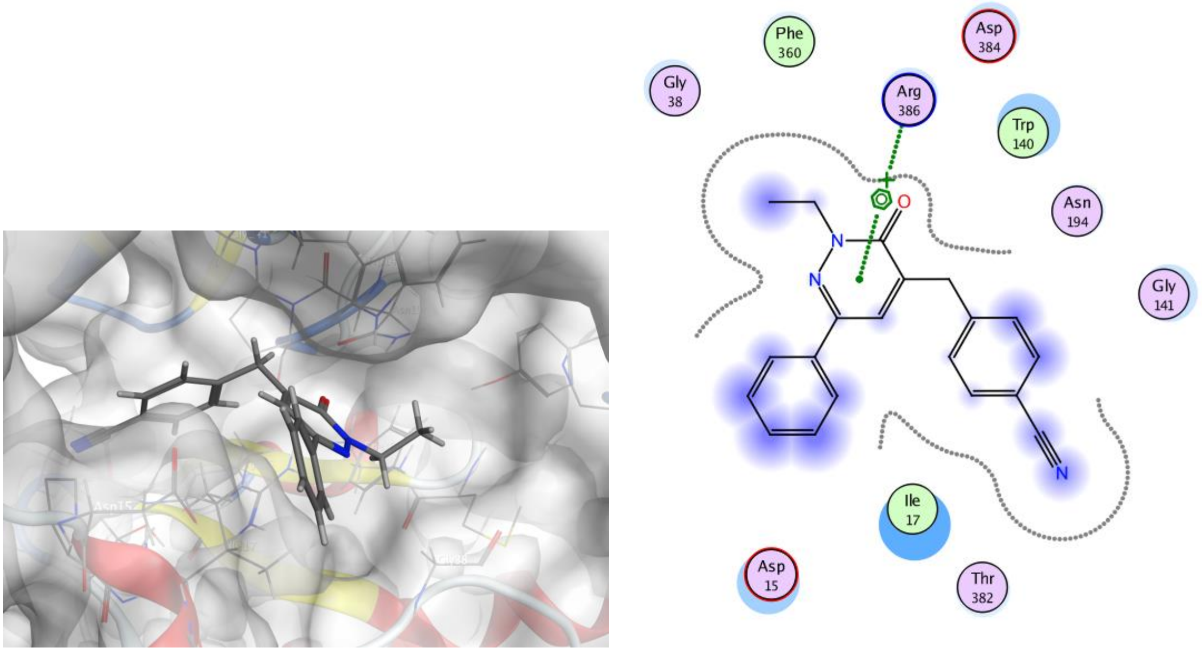

Figure S10. Docking pose of compound **11**.

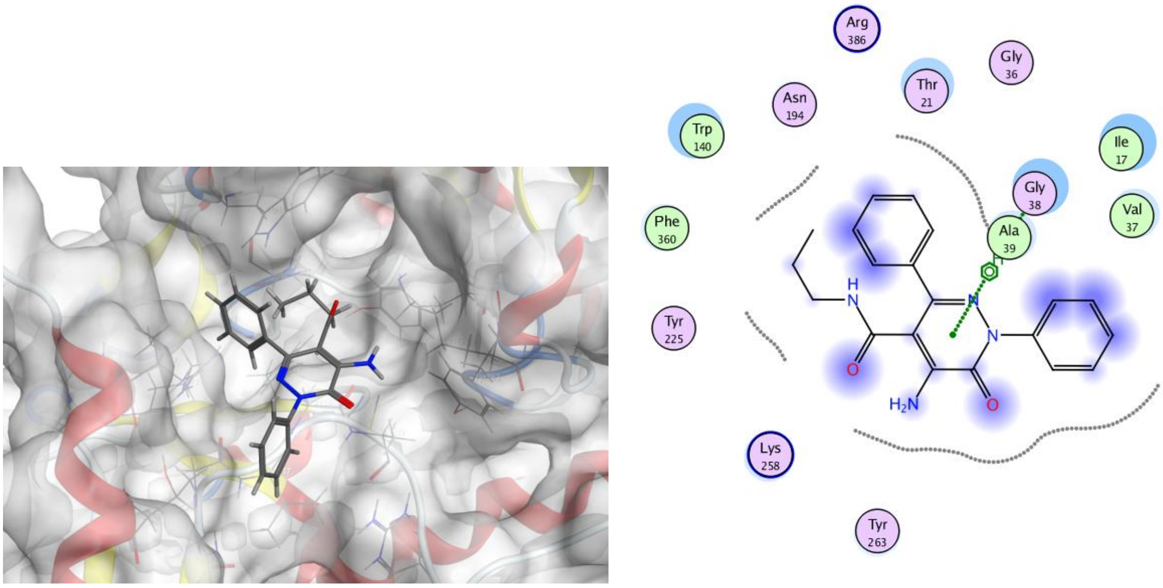

Figure S11. Docking pose of compound **13a**.

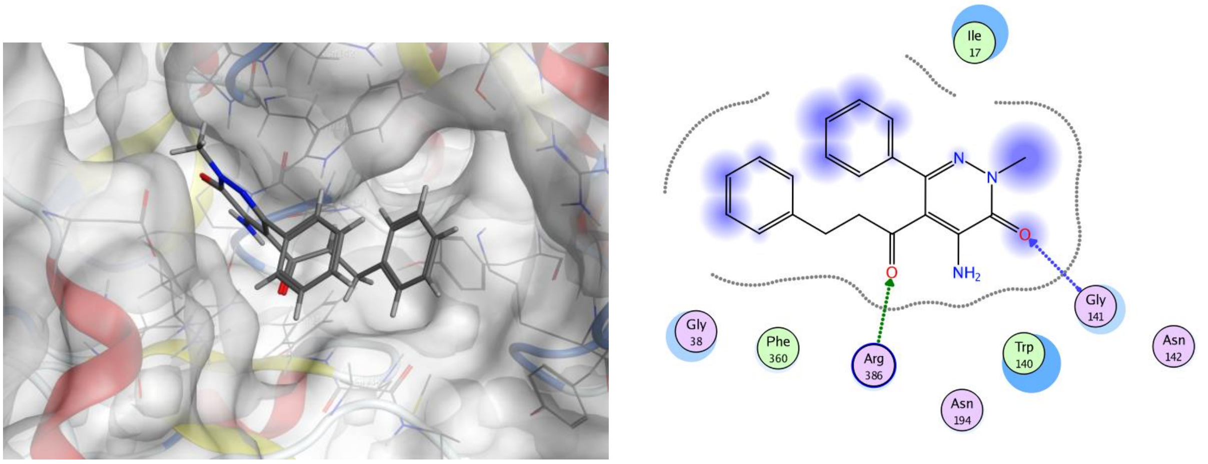

Figure S12. Docking pose of compound **15a**.

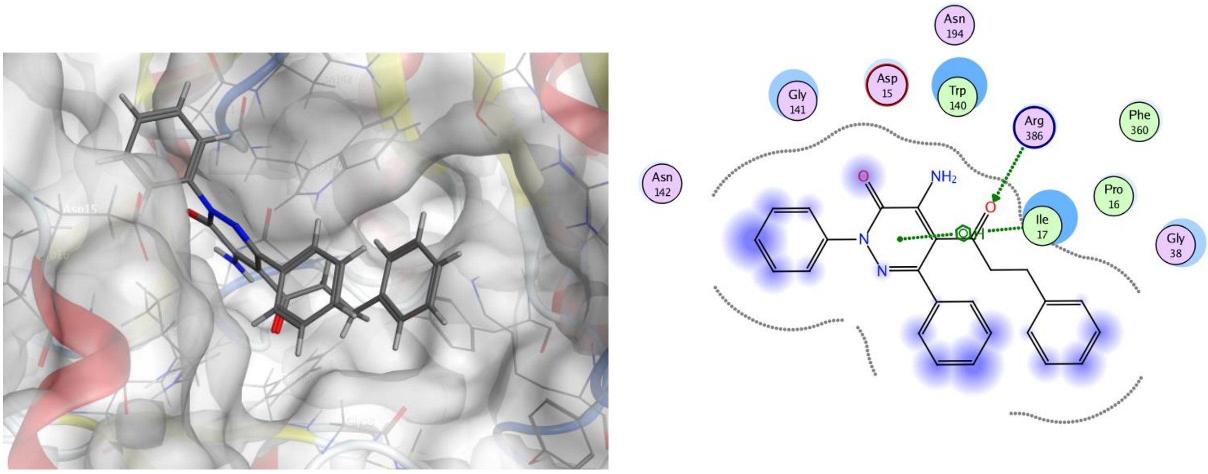

Figure S13. Docking pose of compound **15f**.

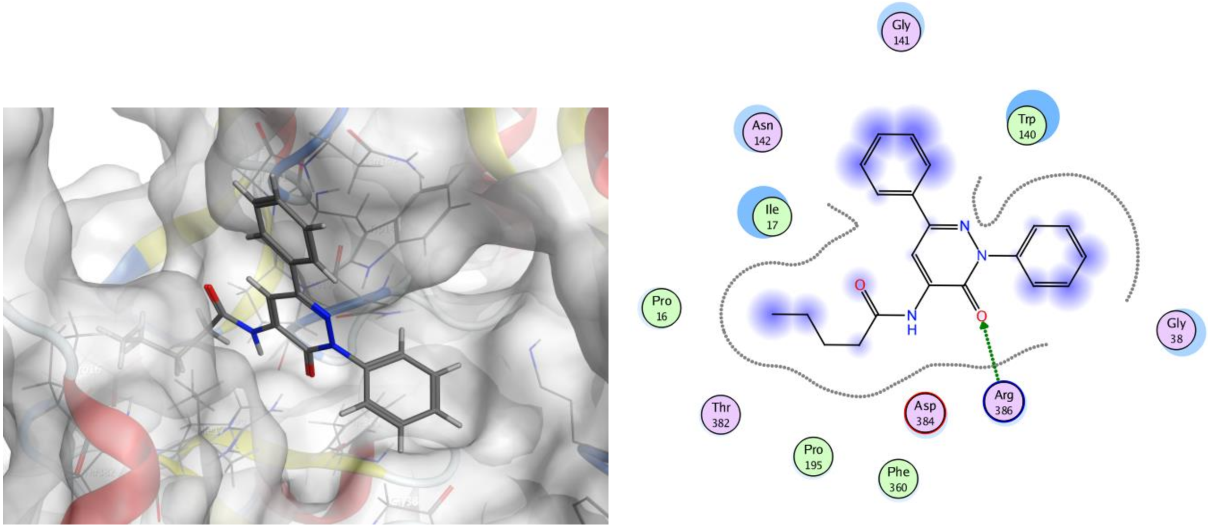

Figure S14. Docking pose of compound **17a**.

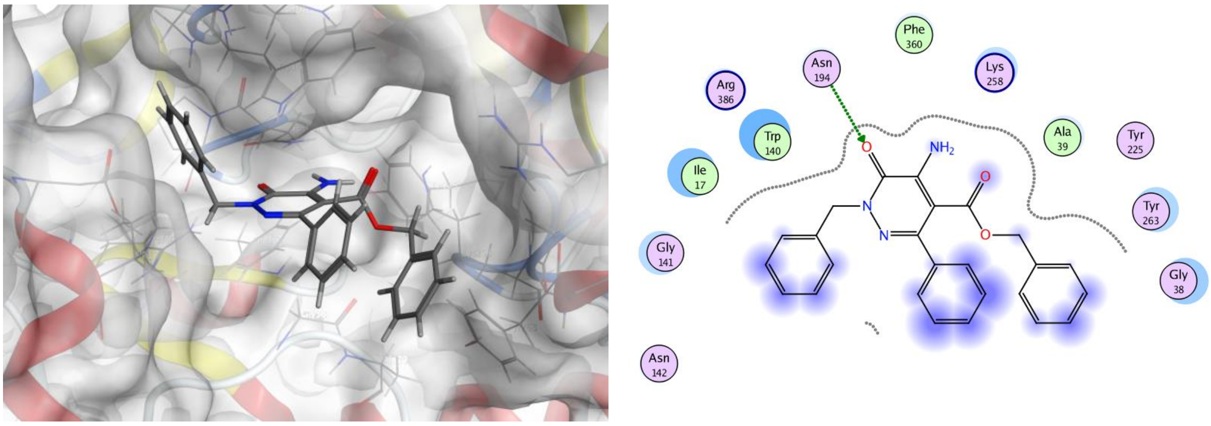

Figure S15. Docking pose of compound **20**.

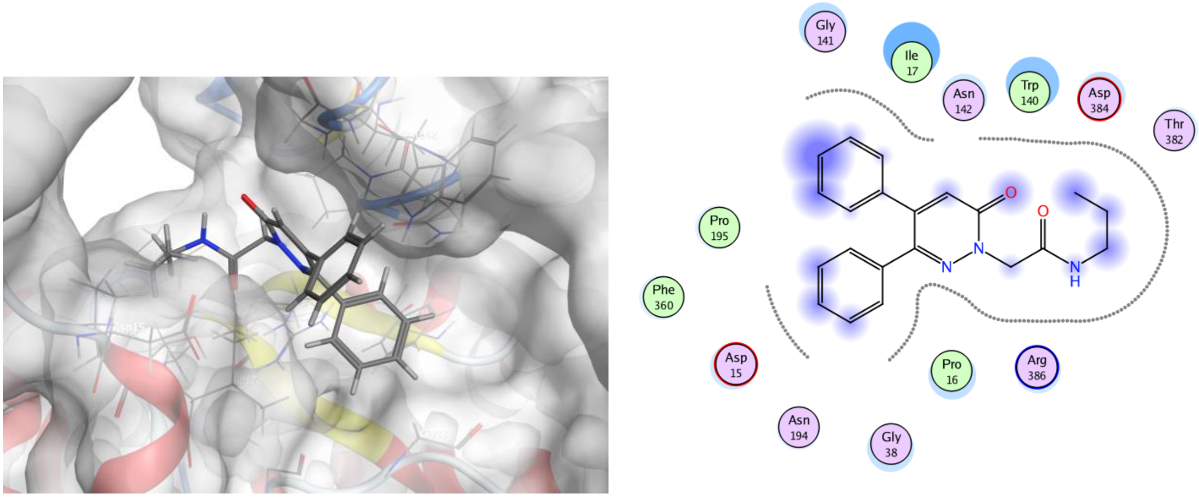

Figure S16. Docking pose of compound **21a**.

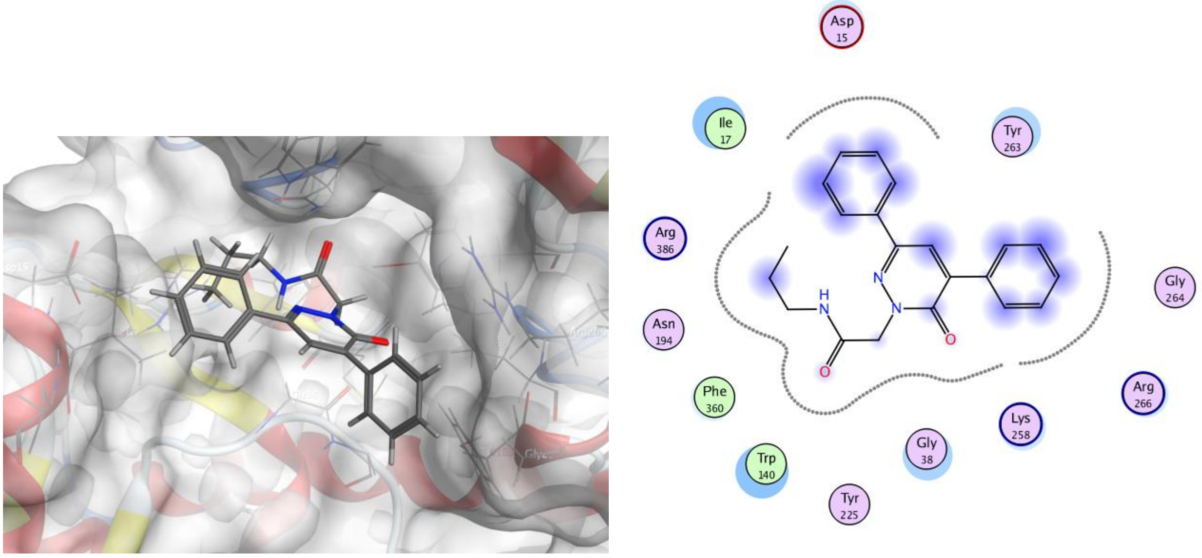

Figure S17. Docking pose of compound **21d**.

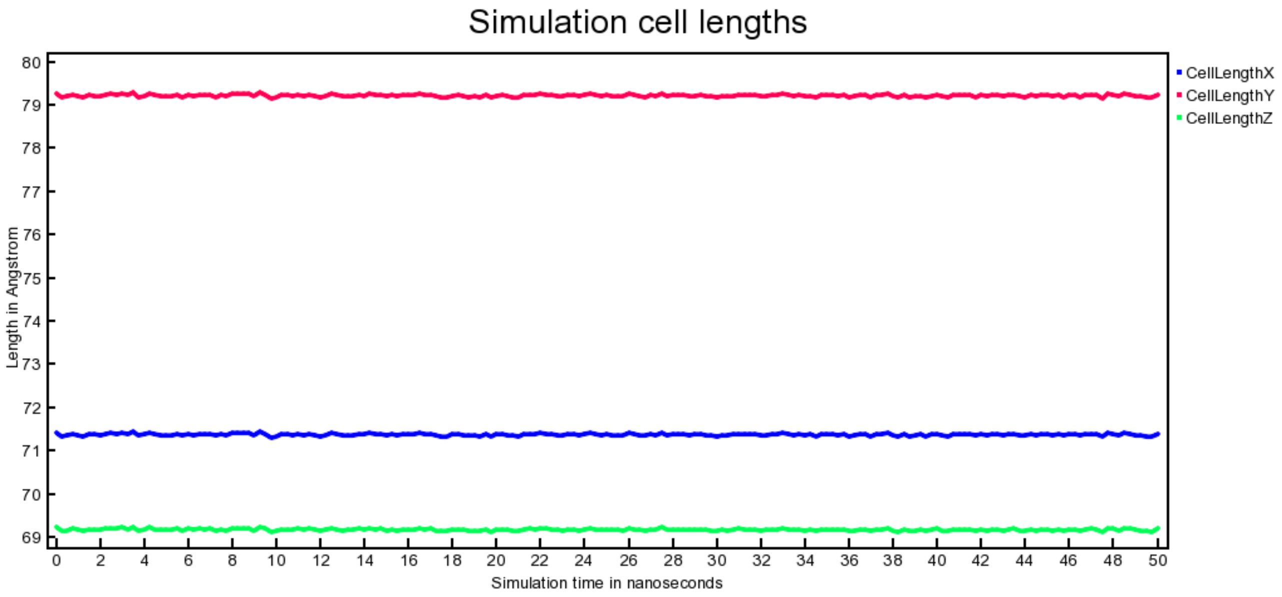

Figure S18. Simulation cell lengths [vertical axis] as a function of simulation time [horizontal axis]

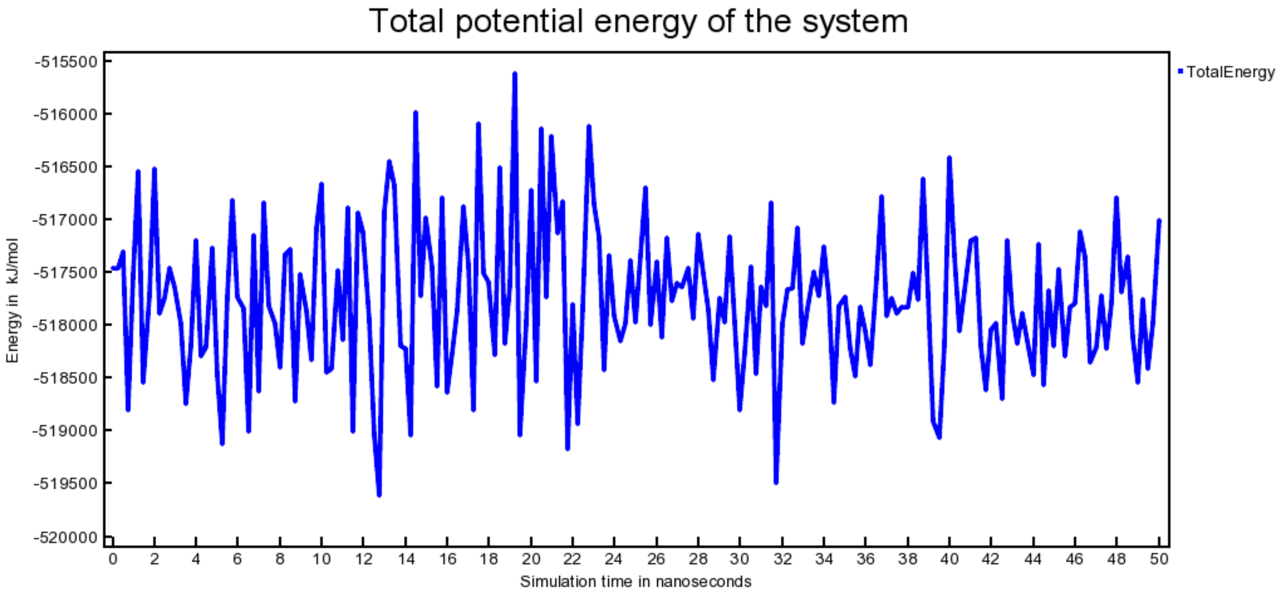

Figure S19. Total potential energy of the system [vertical axis] as a function of simulation time [horizontal axis]. Note: The first value of the plot [-639667.06], coming from the energy minimized starting structure, has been replaced with the second value of the plot [-517460.65] to show this plot with a smaller energy range and thus a higher resolution.

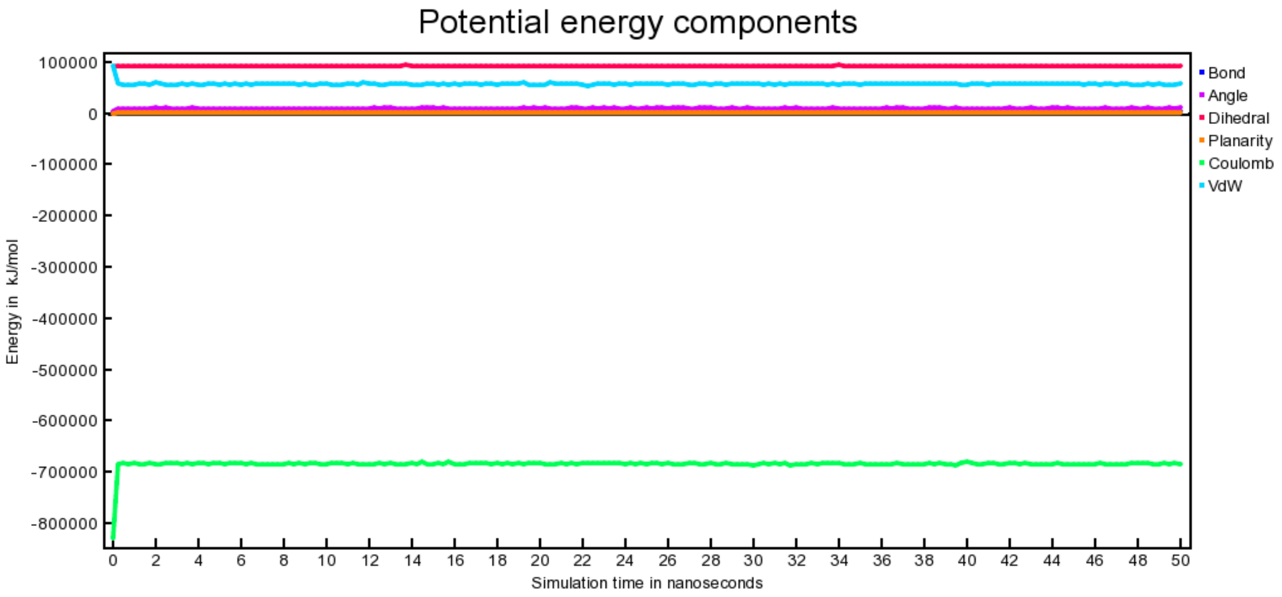

Figure S20. Potential energy components [vertical axis] as a function of simulation time [horizontal axis].

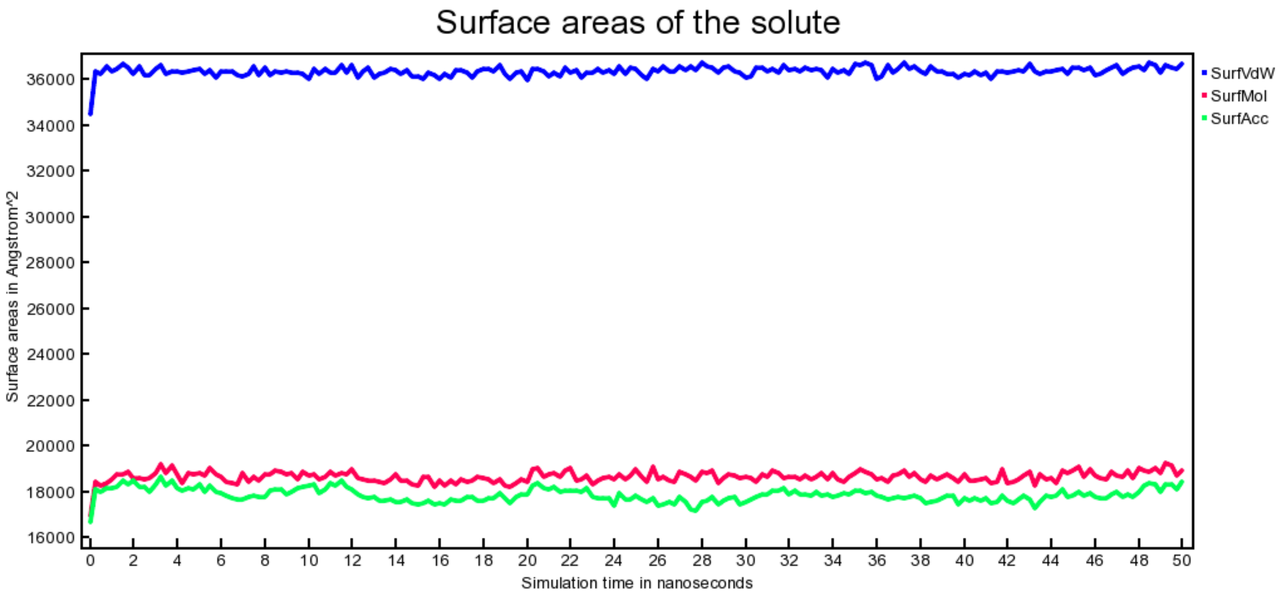

Figure S21. Surface areas of the solute [vertical axis] as a function of simulation time [horizontal axis], obtained with the command "SurfObj Solute".

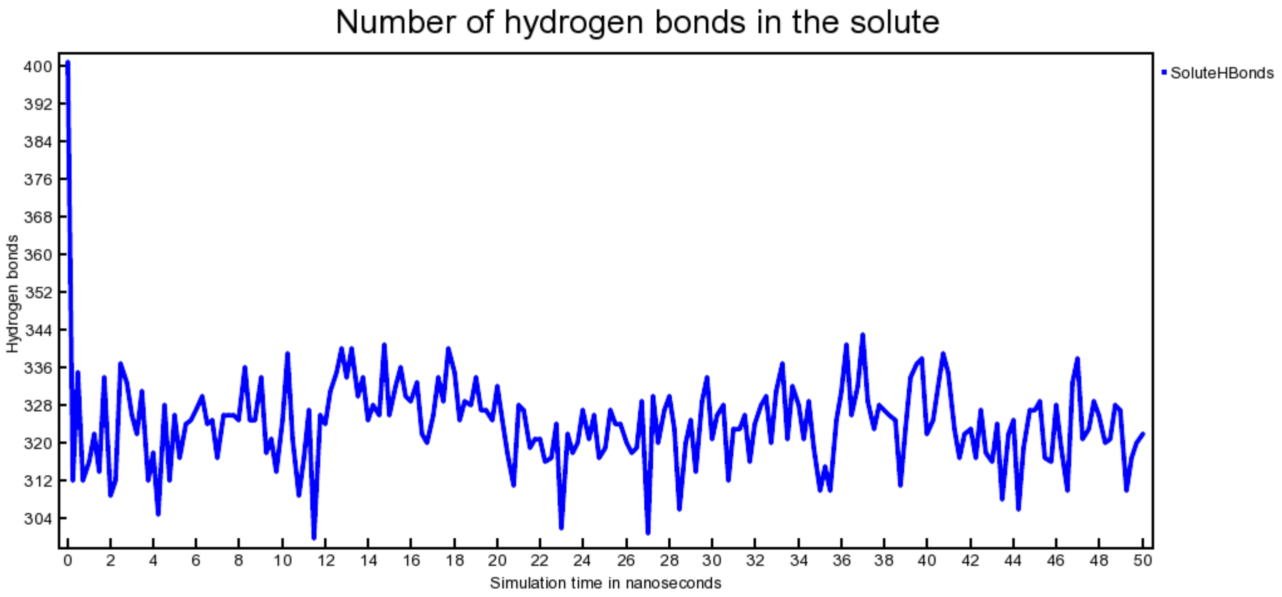

Figure S22. Number of hydrogen bonds in the solute [vertical axis] as a function of simulation time [horizontal axis].

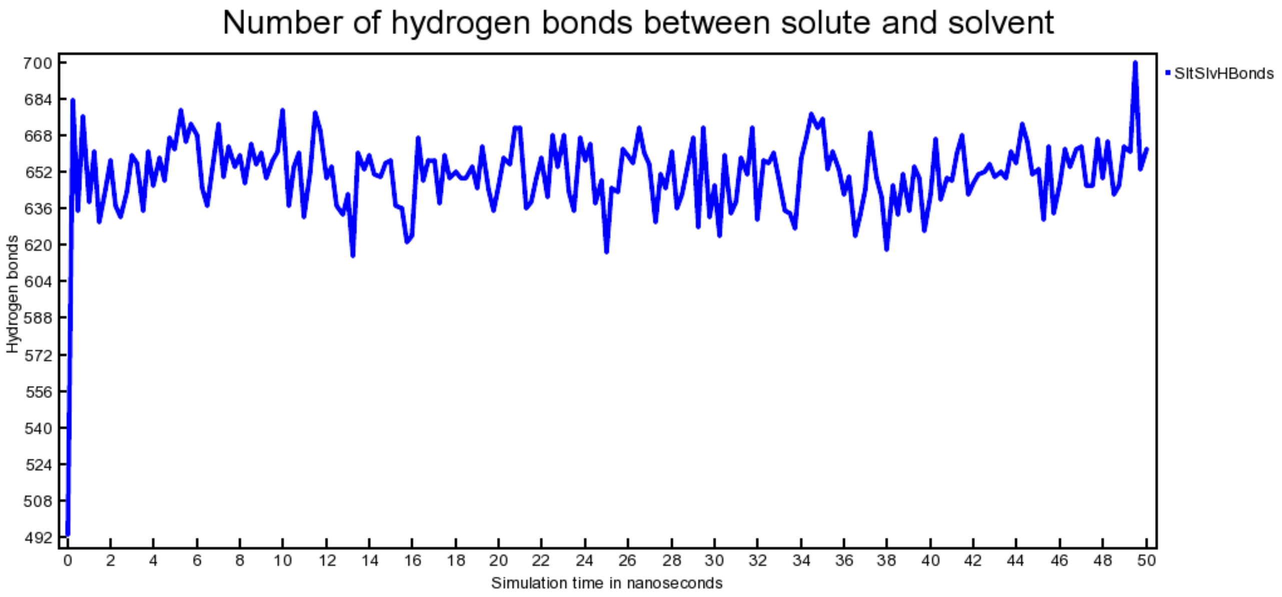

Figure S23. Number of hydrogen bonds between solute and solvent [vertical axis] as a function of simulation time [horizontal axis].

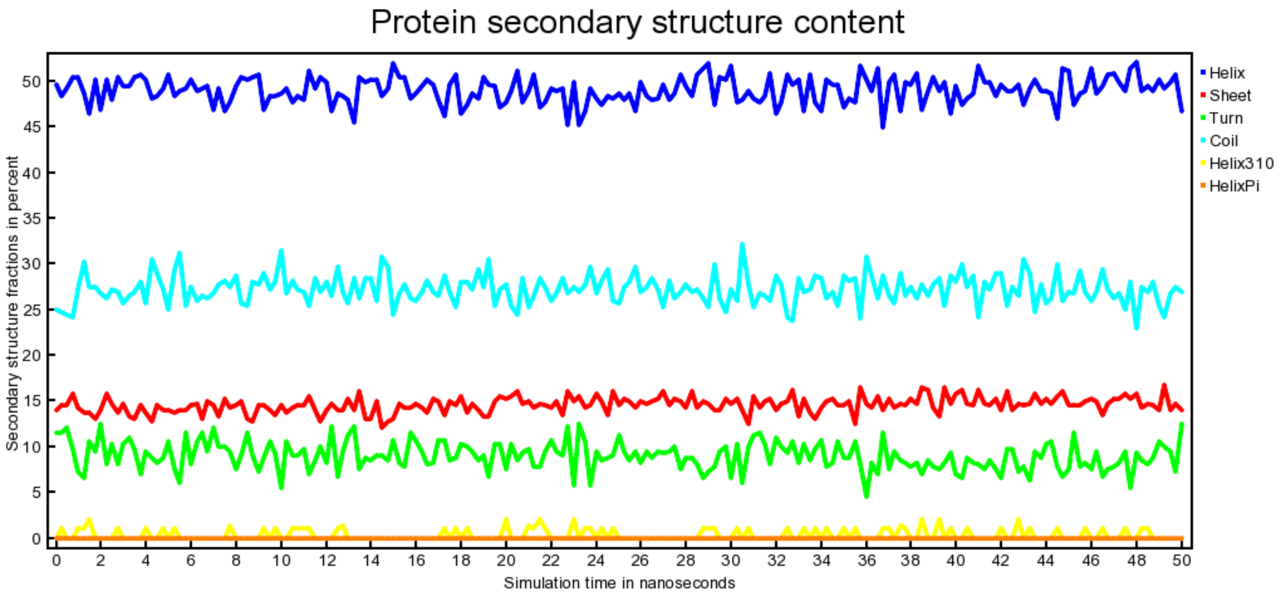

Figure S24. Protein secondary structure content [vertical axis] as a function of simulation time [horizontal axis], obtained with the command "SecStr". Note: Graph HelixPi has all zero values.

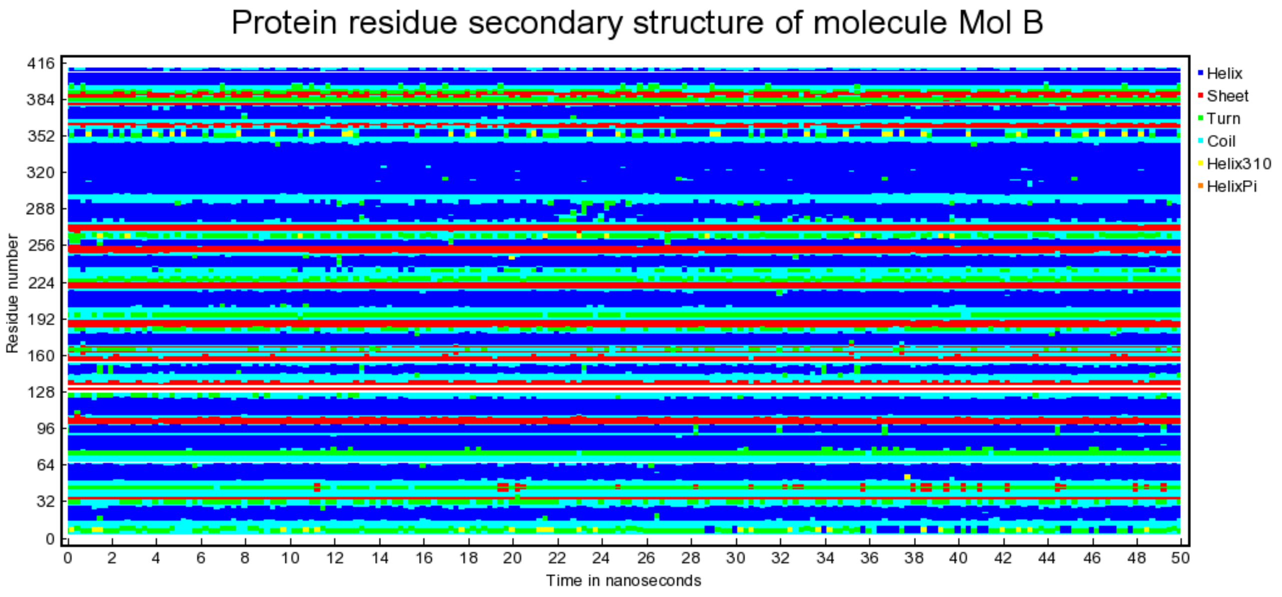

Figure S25. Protein residue secondary structure type [vertical axis] as a function of simulation time [horizontal axis].

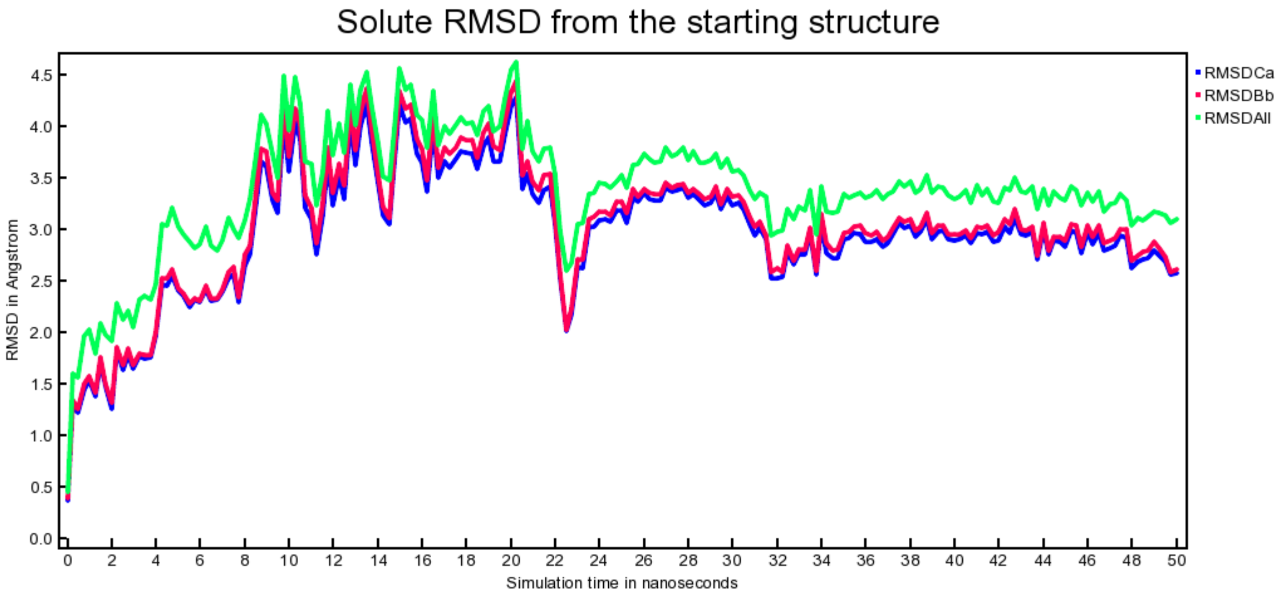

Figure S26. Solute RMSD from the starting structure [vertical axis] as a function of simulation time [horizontal axis].

Figure S27. SwissADME results for molecule **1a**.

1a

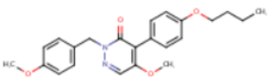

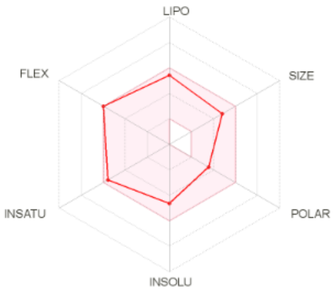

SMILES CCCCOc1ccc(cc1)c1c(OC)cnn(c1=O)Cc1ccc(cc1)OC

Physicochemical Properties	
Formula	C23H26N2O4
Molecular weight	394.46 g/mol
Num. heavy atoms	29
Num. arom. heavy atoms	18
Fraction Csp3	0.30
Num. rotatable bonds	9
Num. H-bond acceptors	5
Num. H-bond donors	0
Molar Refractivity	113.58
TPSA	62.58 Å²

Lipophilicity	
Log $P_{o/w}$ (iLOGP)	4.37
Log $P_{o/w}$ (XLOGP3)	3.97
Log $P_{o/w}$ (WLOGP)	4.15
Log $P_{o/w}$ (MLOGP)	2.87
Log $P_{o/w}$ (SILICOS-IT)	4.73
Consensus Log $P_{o/w}$	4.02

Water Solubility	
Log *S* (ESOL)	-4.65
Solubility	8.79e-03 mg/ml ; 2.23e-05 mol/l
Class	Moderately soluble
Log *S* (Ali)	-4.99
Solubility	4.08e-03 mg/ml ; 1.04e-05 mol/l
Class	Moderately soluble
Log *S* (SILICOS-IT)	-7.70
Solubility	7.93e-06 mg/ml ; 2.01e-08 mol/l
Class	Poorly soluble

Pharmacokinetics	
GI absorption	High
BBB permeant	Yes
P-gp substrate	No
CYP1A2 inhibitor	Yes
CYP2C19 inhibitor	Yes
CYP2C9 inhibitor	Yes
CYP2D6 inhibitor	Yes
CYP3A4 inhibitor	Yes
Log K_p (skin permeation)	-5.89 cm/s

Druglikeness	
Lipinski	Yes; 0 violation
Ghose	Yes
Veber	Yes
Egan	Yes
Muegge	Yes
Bioavailability Score	0.55

Medicinal Chemistry	
PAINS	0 alert
Brenk	0 alert
Leadlikeness	No; 3 violations: MW>350, Rotors>7, XLOGP3>3.5
Synthetic accessibility	3.50

Figure S28. SwissADME results for molecule **2a**.

2a

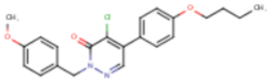

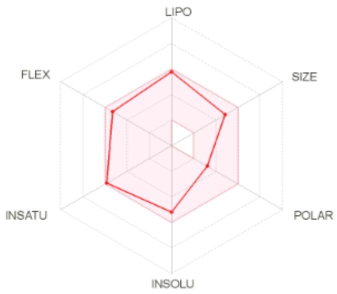

SMILES CCCCOc1ccc(cc1)c1cnn(c(=O)c1Cl)Cc1ccc(cc1)OC

Physicochemical Properties	
Formula	C22H23ClN2O3
Molecular weight	398.88 g/mol
Num. heavy atoms	28
Num. arom. heavy atoms	18
Fraction Csp3	0.27
Num. rotatable bonds	8
Num. H-bond acceptors	4
Num. H-bond donors	0
Molar Refractivity	112.10
TPSA	53.35 Å²

Lipophilicity	
Log $P_{o/w}$ (iLOGP)	4.21
Log $P_{o/w}$ (XLOGP3)	4.63
Log $P_{o/w}$ (WLOGP)	4.80
Log $P_{o/w}$ (MLOGP)	3.68
Log $P_{o/w}$ (SILICOS-IT)	5.31
Consensus Log $P_{o/w}$	4.53

Water Solubility	
Log *S* (ESOL)	-5.18
Solubility	2.65e-03 mg/ml ; 6.64e-06 mol/l
Class	Moderately soluble
Log *S* (Ali)	-5.48
Solubility	1.33e-03 mg/ml ; 3.34e-06 mol/l
Class	Moderately soluble
Log *S* (SILICOS-IT)	-8.18
Solubility	2.62e-06 mg/ml ; 6.58e-09 mol/l
Class	Poorly soluble

Pharmacokinetics	
GI absorption	High
BBB permeant	Yes
P-gp substrate	No
CYP1A2 inhibitor	Yes
CYP2C19 inhibitor	Yes
CYP2C9 inhibitor	Yes
CYP2D6 inhibitor	No
CYP3A4 inhibitor	Yes
Log K_p (skin permeation)	-5.45 cm/s

Druglikeness	
Lipinski	Yes; 0 violation
Ghose	Yes
Veber	Yes
Egan	Yes
Muegge	Yes
Bioavailability Score	0.55

Medicinal Chemistry	
PAINS	0 alert
Brenk	0 alert
Leadlikeness	No; 3 violations: MW>350, Rotors>7, XLOGP3>3.5
Synthetic accessibility	3.20

Figure S29. SwissADME results for molecule **3a**.

3a

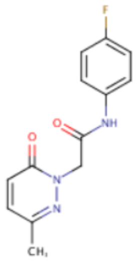

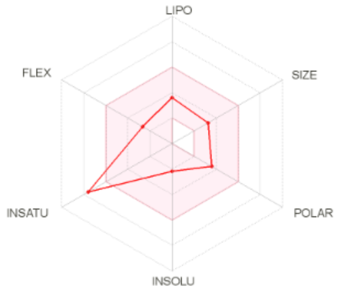

SMILES O=C(Cn1nc(C)ccc1=O)Nc1ccc(cc1)F

Physicochemical Properties	
Formula	C13H12FN3O2
Molecular weight	261.25 g/mol
Num. heavy atoms	19
Num. arom. heavy atoms	12
Fraction Csp3	0.15
Num. rotatable bonds	4
Num. H-bond acceptors	4
Num. H-bond donors	1
Molar Refractivity	68.52
TPSA	63.99 Å²

Lipophilicity	
Log $P_{o/w}$ (iLOGP)	2.37
Log $P_{o/w}$ (XLOGP3)	0.79
Log $P_{o/w}$ (WLOGP)	1.56
Log $P_{o/w}$ (MLOGP)	1.74
Log $P_{o/w}$ (SILICOS-IT)	1.87
Consensus Log $P_{o/w}$	1.67

Water Solubility	
Log *S* (ESOL)	-2.16
Solubility	1.80e+00 mg/ml ; 6.91e-03 mol/l
Class	Soluble
Log *S* (Ali)	-1.71
Solubility	5.04e+00 mg/ml ; 1.93e-02 mol/l
Class	Very soluble
Log *S* (SILICOS-IT)	-4.33
Solubility	1.23e-02 mg/ml ; 4.72e-05 mol/l
Class	Moderately soluble

Pharmacokinetics	
GI absorption	High
BBB permeant	Yes
P-gp substrate	No
CYP1A2 inhibitor	Yes
CYP2C19 inhibitor	No
CYP2C9 inhibitor	No
CYP2D6 inhibitor	No
CYP3A4 inhibitor	No
Log K_p (skin permeation)	-7.33 cm/s

Druglikeness	
Lipinski	Yes; 0 violation
Ghose	Yes
Veber	Yes
Egan	Yes
Muegge	Yes
Bioavailability Score	0.55

Medicinal Chemistry	
PAINS	0 alert
Brenk	0 alert
Leadlikeness	Yes
Synthetic accessibility	2.25

Figure S30. SwissADME results for molecule **5a**.

5a

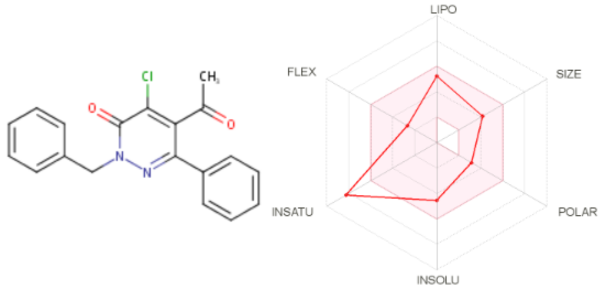

SMILES O=c1n(Cc2ccccc2)nc(c(c1Cl)C(=O)C)c1ccccc1

Physicochemical Properties	
Formula	C19H15ClN2O2
Molecular weight	338.79 g/mol
Num. heavy atoms	24
Num. arom. heavy atoms	18
Fraction Csp3	0.11
Num. rotatable bonds	4
Num. H-bond acceptors	3
Num. H-bond donors	0
Molar Refractivity	94.89
TPSA	51.96 Å²

Lipophilicity	
Log $P_{o/w}$ (iLOGP)	2.85
Log $P_{o/w}$ (XLOGP3)	3.67
Log $P_{o/w}$ (WLOGP)	3.81
Log $P_{o/w}$ (MLOGP)	3.25
Log $P_{o/w}$ (SILICOS-IT)	4.47
Consensus Log $P_{o/w}$	3.61

Water Solubility	
Log *S* (ESOL)	-4.54
Solubility	9.69e-03 mg/ml ; 2.86e-05 mol/l
Class	Moderately soluble
Log *S* (Ali)	-4.45
Solubility	1.20e-02 mg/ml ; 3.54e-05 mol/l
Class	Moderately soluble
Log *S* (SILICOS-IT)	-7.09
Solubility	2.73e-05 mg/ml ; 8.07e-08 mol/l
Class	Poorly soluble

Pharmacokinetics	
GI absorption	High
BBB permeant	Yes
P-gp substrate	No
CYP1A2 inhibitor	Yes
CYP2C19 inhibitor	Yes
CYP2C9 inhibitor	Yes
CYP2D6 inhibitor	No
CYP3A4 inhibitor	No
Log K_p (skin permeation)	-5.76 cm/s

Druglikeness	
Lipinski	Yes; 0 violation
Ghose	Yes
Veber	Yes
Egan	Yes
Muegge	Yes
Bioavailability Score	0.55

Medicinal Chemistry	
PAINS	0 alert
Brenk	0 alert
Leadlikeness	No; 1 violation: XLOGP3>3.5
Synthetic accessibility	2.93

Figure S31. SwissADME results for molecule **7**.

7

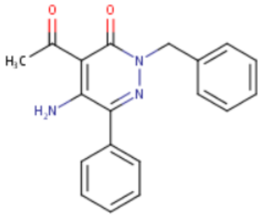

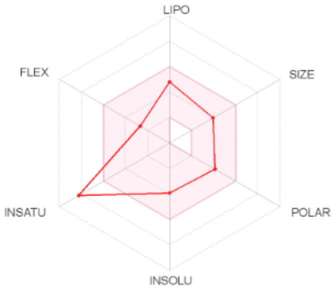

SMILES CC(=O)c1c(N)c(nn(c1=O)Cc1ccccc1)c1ccccc1

Physicochemical Properties	
Formula	C19H17N3O2
Molecular weight	319.36 g/mol
Num. heavy atoms	24
Num. arom. heavy atoms	18
Fraction Csp3	0.11
Num. rotatable bonds	4
Num. H-bond acceptors	3
Num. H-bond donors	1
Molar Refractivity	94.28
TPSA	77.98 Å²

Lipophilicity	
Log $P_{o/w}$ (iLOGP)	2.26
Log $P_{o/w}$ (XLOGP3)	2.91
Log $P_{o/w}$ (WLOGP)	2.75
Log $P_{o/w}$ (MLOGP)	2.19
Log $P_{o/w}$ (SILICOS-IT)	3.12
Consensus Log $P_{o/w}$	2.65

Water Solubility	
Log *S* (ESOL)	-3.94
Solubility	3.63e-02 mg/ml ; 1.14e-04 mol/l
Class	Soluble
Log *S* (Ali)	-4.21
Solubility	1.98e-02 mg/ml ; 6.19e-05 mol/l
Class	Moderately soluble
Log *S* (SILICOS-IT)	-6.13
Solubility	2.37e-04 mg/ml ; 7.41e-07 mol/l
Class	Poorly soluble

Pharmacokinetics	
GI absorption	High
BBB permeant	Yes
P-gp substrate	No
CYP1A2 inhibitor	No
CYP2C19 inhibitor	Yes
CYP2C9 inhibitor	Yes
CYP2D6 inhibitor	No
CYP3A4 inhibitor	No
Log K_p (skin permeation)	-6.18 cm/s

Druglikeness	
Lipinski	Yes; 0 violation
Ghose	Yes
Veber	Yes
Egan	Yes
Muegge	Yes
Bioavailability Score	0.55

Medicinal Chemistry	
PAINS	0 alert
Brenk	0 alert
Leadlikeness	Yes
Synthetic accessibility	2.99

Figure S32. SwissADME results for molecule **8**.

8

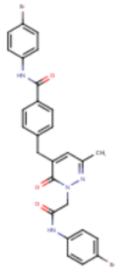

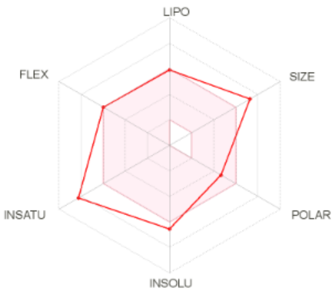

SMILES Brc1ccc(cc1)NC(=O)Cn1nc(C)cc(c1=O)Cc1ccc(cc1)C(=O)Nc1ccc(c c1)Br

Physicochemical Properties	
Formula	C27H22Br2N4O3
Molecular weight	610.30 g/mol
Num. heavy atoms	36
Num. arom. heavy atoms	24
Fraction Csp3	0.11
Num. rotatable bonds	9
Num. H-bond acceptors	4
Num. H-bond donors	2
Molar Refractivity	147.63
TPSA	93.09 Å²

Lipophilicity	
Log $P_{o/w}$ (iLOGP)	4.14
Log $P_{o/w}$ (XLOGP3)	4.83
Log $P_{o/w}$ (WLOGP)	5.18
Log $P_{o/w}$ (MLOGP)	4.48
Log $P_{o/w}$ (SILICOS-IT)	5.55
Consensus Log $P_{o/w}$	4.84

Water Solubility	
Log *S* (ESOL)	-6.57
Solubility	1.66e-04 mg/ml ; 2.72e-07 mol/l
Class	Poorly soluble
Log *S* (Ali)	-6.52
Solubility	1.85e-04 mg/ml ; 3.03e-07 mol/l
Class	Poorly soluble
Log *S* (SILICOS-IT)	-10.87
Solubility	8.21e-09 mg/ml ; 1.35e-11 mol/l
Class	Insoluble

Pharmacokinetics	
GI absorption	High
BBB permeant	No
P-gp substrate	No
CYP1A2 inhibitor	No
CYP2C19 inhibitor	Yes
CYP2C9 inhibitor	Yes
CYP2D6 inhibitor	No
CYP3A4 inhibitor	Yes
Log K_p (skin permeation)	-6.59 cm/s

Druglikeness	
Lipinski	No; 2 violations: MW>500, MLOGP>4.15
Ghose	No; 2 violations: MW>480, MR>130
Veber	Yes
Egan	Yes
Muegge	No; 1 violation: MW>600
Bioavailability Score	0.17

Medicinal Chemistry	
PAINS	0 alert
Brenk	0 alert
Leadlikeness	No; 3 violations: MW>350, Rotors>7, XLOGP3>3.5
Synthetic accessibility	3.45

Figure S33. SwissADME results for molecule **9**.

9

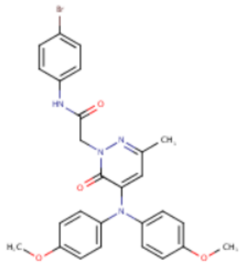

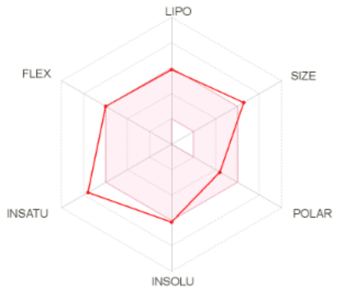

SMILES	COc1ccc(cc1)N(c1cc(C)nn(c1=O)CC(=O)Nc1ccc(cc1)Br)c1ccc(cc1)OC

Physicochemical Properties	
Formula	C27H25BrN4O4
Molecular weight	549.42 g/mol
Num. heavy atoms	36
Num. arom. heavy atoms	24
Fraction Csp3	0.15
Num. rotatable bonds	9
Num. H-bond acceptors	5
Num. H-bond donors	1
Molar Refractivity	143.93
TPSA	85.69 Å²

Lipophilicity	
Log $P_{o/w}$ (iLOGP)	4.24
Log $P_{o/w}$ (XLOGP3)	4.77
Log $P_{o/w}$ (WLOGP)	5.25
Log $P_{o/w}$ (MLOGP)	3.60
Log $P_{o/w}$ (SILICOS-IT)	4.09
Consensus Log $P_{o/w}$	4.39

Water Solubility	
Log *S* (ESOL)	-6.15
Solubility	3.88e-04 mg/ml ; 7.07e-07 mol/l
Class	Poorly soluble
Log *S* (Ali)	-6.30
Solubility	2.75e-04 mg/ml ; 5.01e-07 mol/l
Class	Poorly soluble
Log *S* (SILICOS-IT)	-9.29
Solubility	2.85e-07 mg/ml ; 5.19e-10 mol/l
Class	Poorly soluble

Pharmacokinetics	
GI absorption	High
BBB permeant	No
P-gp substrate	No
CYP1A2 inhibitor	No
CYP2C19 inhibitor	Yes
CYP2C9 inhibitor	Yes
CYP2D6 inhibitor	No
CYP3A4 inhibitor	Yes
Log K_p (skin permeation)	-6.26 cm/s

Druglikeness	
Lipinski	Yes; 1 violation: MW>500
Ghose	No; 2 violations: MW>480, MR>130
Veber	Yes
Egan	Yes
Muegge	Yes
Bioavailability Score	0.55

Medicinal Chemistry	
PAINS	0 alert
Brenk	0 alert
Leadlikeness	No; 3 violations: MW>350, Rotors>7, XLOGP3>3.5
Synthetic accessibility	3.76

Figure S34. SwissADME results for molecule **10b**.

10b

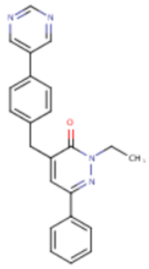

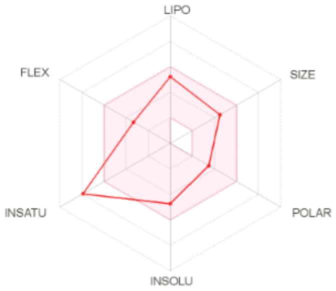

SMILES CCn1nc(cc(c1=O)Cc1ccc(cc1)c1cncnc1)c1ccccc1

Physicochemical Properties	
Formula	C23H20N4O
Molecular weight	368.43 g/mol
Num. heavy atoms	28
Num. arom. heavy atoms	24
Fraction Csp3	0.13
Num. rotatable bonds	5
Num. H-bond acceptors	4
Num. H-bond donors	0
Molar Refractivity	110.48
TPSA	60.67 Å²

Lipophilicity	
Log $P_{o/w}$ (iLOGP)	3.49
Log $P_{o/w}$ (XLOGP3)	3.65
Log $P_{o/w}$ (WLOGP)	3.98
Log $P_{o/w}$ (MLOGP)	3.07
Log $P_{o/w}$ (SILICOS-IT)	4.74
Consensus Log $P_{o/w}$	3.79

Water Solubility	
Log *S* (ESOL)	-4.73
Solubility	6.89e-03 mg/ml ; 1.87e-05 mol/l
Class	Moderately soluble
Log *S* (Ali)	-4.61
Solubility	8.98e-03 mg/ml ; 2.44e-05 mol/l
Class	Moderately soluble
Log *S* (SILICOS-IT)	-8.68
Solubility	7.63e-07 mg/ml ; 2.07e-09 mol/l
Class	Poorly soluble

Pharmacokinetics	
GI absorption	High
BBB permeant	Yes
P-gp substrate	No
CYP1A2 inhibitor	Yes
CYP2C19 inhibitor	Yes
CYP2C9 inhibitor	Yes
CYP2D6 inhibitor	No
CYP3A4 inhibitor	Yes
Log K_p (skin permeation)	-5.96 cm/s

Druglikeness	
Lipinski	Yes; 0 violation
Ghose	Yes
Veber	Yes
Egan	Yes
Muegge	Yes
Bioavailability Score	0.55

Medicinal Chemistry	
PAINS	0 alert
Brenk	0 alert
Leadlikeness	No; 2 violations: MW>350, XLOGP3>3.5
Synthetic accessibility	3.25

Figure S35. SwissADME results for molecule **10g**.

10g

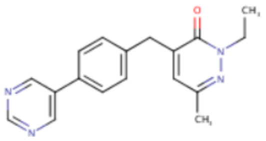

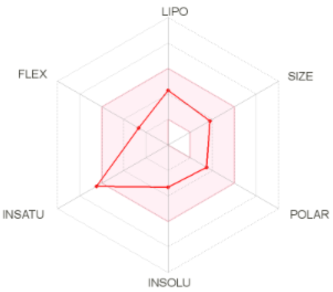

SMILES CCn1nc(C)cc(c1=O)Cc1ccc(cc1)c1cncnc1

Physicochemical Properties	
Formula	C18H18N4O
Molecular weight	306.36 g/mol
Num. heavy atoms	23
Num. arom. heavy atoms	18
Fraction Csp3	0.22
Num. rotatable bonds	4
Num. H-bond acceptors	4
Num. H-bond donors	0
Molar Refractivity	90.01
TPSA	60.67 Å²

Lipophilicity	
Log $P_{o/w}$ (iLOGP)	2.97
Log $P_{o/w}$ (XLOGP3)	1.99
Log $P_{o/w}$ (WLOGP)	2.62
Log $P_{o/w}$ (MLOGP)	2.17
Log $P_{o/w}$ (SILICOS-IT)	3.69
Consensus Log $P_{o/w}$	2.69

Water Solubility	
Log *S* (ESOL)	-3.31
Solubility	1.51e-01 mg/ml ; 4.92e-04 mol/l
Class	Soluble
Log *S* (Ali)	-2.89
Solubility	3.94e-01 mg/ml ; 1.29e-03 mol/l
Class	Soluble
Log *S* (SILICOS-IT)	-6.59
Solubility	7.80e-05 mg/ml ; 2.55e-07 mol/l
Class	Poorly soluble

Pharmacokinetics	
GI absorption	High
BBB permeant	Yes
P-gp substrate	No
CYP1A2 inhibitor	No
CYP2C19 inhibitor	Yes
CYP2C9 inhibitor	Yes
CYP2D6 inhibitor	No
CYP3A4 inhibitor	Yes
Log K_p (skin permeation)	-6.76 cm/s

Druglikeness	
Lipinski	Yes; 0 violation
Ghose	Yes
Veber	Yes
Egan	Yes
Muegge	Yes
Bioavailability Score	0.55

Medicinal Chemistry	
PAINS	0 alert
Brenk	0 alert
Leadlikeness	Yes
Synthetic accessibility	2.79

Figure S36. SwissADME results for molecule **11**.

11

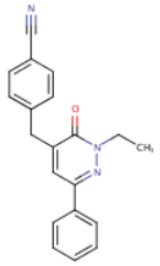

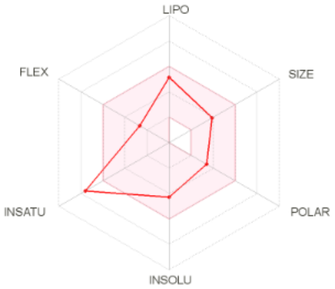

SMILES CCn1nc(cc(c1=O)Cc1ccc(cc1)C#N)c1ccccc1

Physicochemical Properties	
Formula	C20H17N3O
Molecular weight	315.37 g/mol
Num. heavy atoms	24
Num. arom. heavy atoms	18
Fraction Csp3	0.15
Num. rotatable bonds	4
Num. H-bond acceptors	3
Num. H-bond donors	0
Molar Refractivity	94.17
TPSA	58.68 Å²

Lipophilicity	
Log $P_{o/w}$ (iLOGP)	3.24
Log $P_{o/w}$ (XLOGP3)	3.46
Log $P_{o/w}$ (WLOGP)	3.39
Log $P_{o/w}$ (MLOGP)	2.98
Log $P_{o/w}$ (SILICOS-IT)	4.33
Consensus Log $P_{o/w}$	3.48

Water Solubility	
Log *S* (ESOL)	-4.27
Solubility	1.71e-02 mg/ml ; 5.42e-05 mol/l
Class	Moderately soluble
Log *S* (Ali)	-4.37
Solubility	1.33e-02 mg/ml ; 4.23e-05 mol/l
Class	Moderately soluble
Log *S* (SILICOS-IT)	-7.03
Solubility	2.92e-05 mg/ml ; 9.27e-08 mol/l
Class	Poorly soluble

Pharmacokinetics	
GI absorption	High
BBB permeant	Yes
P-gp substrate	No
CYP1A2 inhibitor	Yes
CYP2C19 inhibitor	Yes
CYP2C9 inhibitor	Yes
CYP2D6 inhibitor	No
CYP3A4 inhibitor	No
Log K_p (skin permeation)	-5.77 cm/s

Druglikeness	
Lipinski	Yes; 0 violation
Ghose	Yes
Veber	Yes
Egan	Yes
Muegge	Yes
Bioavailability Score	0.55

Medicinal Chemistry	
PAINS	0 alert
Brenk	0 alert
Leadlikeness	Yes
Synthetic accessibility	3.03

Figure S37. SwissADME results for molecule **13a**.

13a

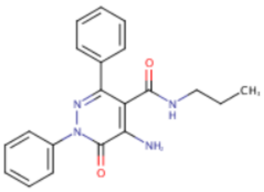

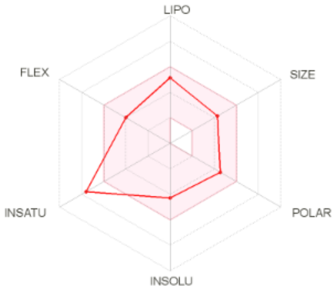

SMILES CCCNC(=O)c1c(nn(c(=O)c1N)c1ccccc1)c1ccccc1

Physicochemical Properties	
Formula	C20H20N4O2
Molecular weight	348.40 g/mol
Num. heavy atoms	26
Num. arom. heavy atoms	18
Fraction Csp3	0.15
Num. rotatable bonds	6
Num. H-bond acceptors	3
Num. H-bond donors	2
Molar Refractivity	102.28
TPSA	90.01 Å²

Lipophilicity	
Log $P_{o/w}$ (iLOGP)	2.44
Log $P_{o/w}$ (XLOGP3)	3.46
Log $P_{o/w}$ (WLOGP)	2.63
Log $P_{o/w}$ (MLOGP)	2.55
Log $P_{o/w}$ (SILICOS-IT)	2.72
Consensus Log $P_{o/w}$	2.76

Water Solubility	
Log *S* (ESOL)	-4.30
Solubility	1.76e-02 mg/ml ; 5.06e-05 mol/l
Class	Moderately soluble
Log *S* (Ali)	-5.03
Solubility	3.24e-03 mg/ml ; 9.29e-06 mol/l
Class	Moderately soluble
Log *S* (SILICOS-IT)	-6.57
Solubility	9.42e-05 mg/ml ; 2.70e-07 mol/l
Class	Poorly soluble

Pharmacokinetics	
GI absorption	High
BBB permeant	No
P-gp substrate	No
CYP1A2 inhibitor	No
CYP2C19 inhibitor	No
CYP2C9 inhibitor	Yes
CYP2D6 inhibitor	No
CYP3A4 inhibitor	Yes
Log K_p (skin permeation)	-5.97 cm/s

Druglikeness	
Lipinski	Yes; 0 violation
Ghose	Yes
Veber	Yes
Egan	Yes
Muegge	Yes
Bioavailability Score	0.55

Medicinal Chemistry	
PAINS	0 alert
Brenk	0 alert
Leadlikeness	Yes
Synthetic accessibility	3.21

Figure S38. SwissADME results for molecule **15a**.

15a

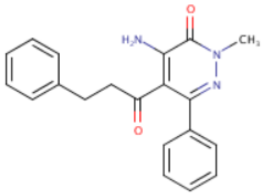

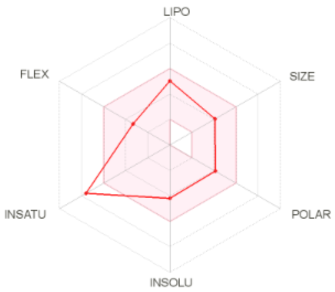

SMILES O=c1n(C)nc(c(c1N)C(=O)CCc1ccccc1)c1ccccc1

Physicochemical Properties	
Formula	C20H19N3O2
Molecular weight	333.38 g/mol
Num. heavy atoms	25
Num. arom. heavy atoms	18
Fraction Csp3	0.15
Num. rotatable bonds	5
Num. H-bond acceptors	3
Num. H-bond donors	1
Molar Refractivity	99.09
TPSA	77.98 Å²

Lipophilicity	
Log $P_{o/w}$ (iLOGP)	2.63
Log $P_{o/w}$ (XLOGP3)	3.31
Log $P_{o/w}$ (WLOGP)	2.85
Log $P_{o/w}$ (MLOGP)	2.42
Log $P_{o/w}$ (SILICOS-IT)	3.50
Consensus Log $P_{o/w}$	2.94

Water Solubility	
Log *S* (ESOL)	-4.20
Solubility	2.13e-02 mg/ml ; 6.38e-05 mol/l
Class	Moderately soluble
Log *S* (Ali)	-4.62
Solubility	7.93e-03 mg/ml ; 2.38e-05 mol/l
Class	Moderately soluble
Log *S* (SILICOS-IT)	-6.53
Solubility	9.93e-05 mg/ml ; 2.98e-07 mol/l
Class	Poorly soluble

Pharmacokinetics	
GI absorption	High
BBB permeant	Yes
P-gp substrate	No
CYP1A2 inhibitor	No
CYP2C19 inhibitor	Yes
CYP2C9 inhibitor	Yes
CYP2D6 inhibitor	No
CYP3A4 inhibitor	No
Log K_p (skin permeation)	-5.98 cm/s

Druglikeness	
Lipinski	Yes; 0 violation
Ghose	Yes
Veber	Yes
Egan	Yes
Muegge	Yes
Bioavailability Score	0.55

Medicinal Chemistry	
PAINS	0 alert
Brenk	0 alert
Leadlikeness	Yes
Synthetic accessibility	3.19

Figure S39. SwissADME results for molecule **15f**.

15f

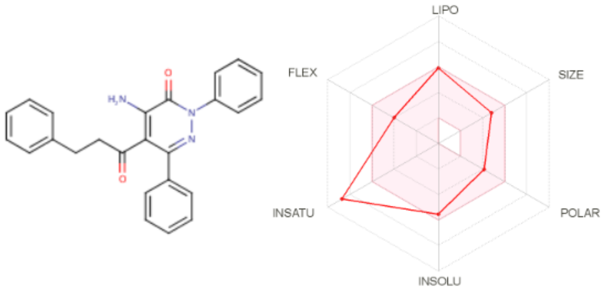

SMILES O=c1c(N)c(C(=O)CCc2ccccc2)c(nn1c1ccccc1)c1ccccc1

Physicochemical Properties	
Formula	C25H21N3O2
Molecular weight	395.45 g/mol
Num. heavy atoms	30
Num. arom. heavy atoms	24
Fraction Csp3	0.08
Num. rotatable bonds	6
Num. H-bond acceptors	3
Num. H-bond donors	1
Molar Refractivity	119.16
TPSA	77.98 Å²

Lipophilicity	
Log $P_{o/w}$ (iLOGP)	3.44
Log $P_{o/w}$ (XLOGP3)	4.86
Log $P_{o/w}$ (WLOGP)	4.31
Log $P_{o/w}$ (MLOGP)	3.55
Log $P_{o/w}$ (SILICOS-IT)	4.56
Consensus Log $P_{o/w}$	4.14

Water Solubility	
Log *S* (ESOL)	-5.55
Solubility	1.12e-03 mg/ml ; 2.82e-06 mol/l
Class	Moderately soluble
Log *S* (Ali)	-6.23
Solubility	2.32e-04 mg/ml ; 5.86e-07 mol/l
Class	Poorly soluble
Log *S* (SILICOS-IT)	-8.61
Solubility	9.71e-07 mg/ml ; 2.45e-09 mol/l
Class	Poorly soluble

Pharmacokinetics	
GI absorption	High
BBB permeant	No
P-gp substrate	No
CYP1A2 inhibitor	Yes
CYP2C19 inhibitor	Yes
CYP2C9 inhibitor	Yes
CYP2D6 inhibitor	No
CYP3A4 inhibitor	Yes
Log K_p (skin permeation)	-5.26 cm/s

Druglikeness	
Lipinski	Yes; 0 violation
Ghose	Yes
Veber	Yes
Egan	Yes
Muegge	Yes
Bioavailability Score	0.55

Medicinal Chemistry	
PAINS	0 alert
Brenk	0 alert
Leadlikeness	No; 2 violations: MW>350, XLOGP3>3.5
Synthetic accessibility	3.45

Figure S40. SwissADME results for molecule **17a**.

17a

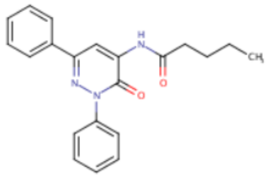

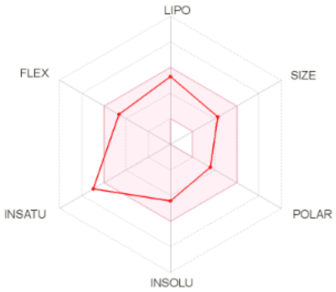

SMILES CCCCC(=O)Nc1cc(nn(c1=O)c1ccccc1)c1ccccc1

Physicochemical Properties	
Formula	C21H21N3O2
Molecular weight	347.41 g/mol
Num. heavy atoms	26
Num. arom. heavy atoms	18
Fraction Csp3	0.19
Num. rotatable bonds	7
Num. H-bond acceptors	3
Num. H-bond donors	1
Molar Refractivity	104.00
TPSA	63.99 Å²

Lipophilicity	
Log $P_{o/w}$ (iLOGP)	3.63
Log $P_{o/w}$ (XLOGP3)	3.76
Log $P_{o/w}$ (WLOGP)	3.84
Log $P_{o/w}$ (MLOGP)	3.32
Log $P_{o/w}$ (SILICOS-IT)	3.83
Consensus Log $P_{o/w}$	3.67

Water Solubility	
Log *S* (ESOL)	-4.41
Solubility	1.34e-02 mg/ml ; 3.86e-05 mol/l
Class	Moderately soluble
Log *S* (Ali)	-4.80
Solubility	5.55e-03 mg/ml ; 1.60e-05 mol/l
Class	Moderately soluble
Log *S* (SILICOS-IT)	-7.33
Solubility	1.62e-05 mg/ml ; 4.66e-08 mol/l
Class	Poorly soluble

Pharmacokinetics	
GI absorption	High
BBB permeant	Yes
P-gp substrate	No
CYP1A2 inhibitor	Yes
CYP2C19 inhibitor	Yes
CYP2C9 inhibitor	Yes
CYP2D6 inhibitor	No
CYP3A4 inhibitor	Yes
Log K_p (skin permeation)	-5.75 cm/s

Druglikeness	
Lipinski	Yes; 0 violation
Ghose	Yes
Veber	Yes
Egan	Yes
Muegge	Yes
Bioavailability Score	0.55

Medicinal Chemistry	
PAINS	0 alert
Brenk	0 alert
Leadlikeness	No; 1 violation: XLOGP3>3.5
Synthetic accessibility	3.09

Figure S41. SwissADME results for molecule **20**.

20

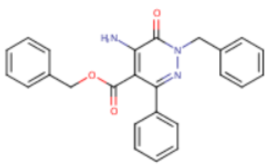

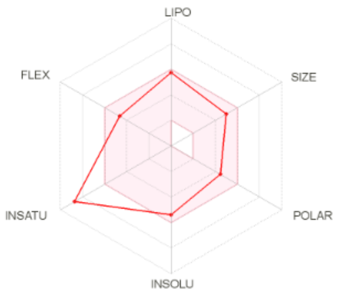

SMILES O=C(c1c(nn(c(=O)c1N)Cc1ccccc1)c1ccccc1)OCc1ccccc1

Physicochemical Properties	
Formula	C25H21N3O3
Molecular weight	411.45 g/mol
Num. heavy atoms	31
Num. arom. heavy atoms	24
Fraction Csp3	0.08
Num. rotatable bonds	7
Num. H-bond acceptors	4
Num. H-bond donors	1
Molar Refractivity	119.85
TPSA	87.21 Å²

Lipophilicity	
Log $P_{o/w}$ (iLOGP)	3.50
Log $P_{o/w}$ (XLOGP3)	4.57
Log $P_{o/w}$ (WLOGP)	3.75
Log $P_{o/w}$ (MLOGP)	3.55
Log $P_{o/w}$ (SILICOS-IT)	4.09
Consensus Log $P_{o/w}$	3.89

Water Solubility	
Log *S* (ESOL)	-5.38
Solubility	1.71e-03 mg/ml ; 4.16e-06 mol/l
Class	Moderately soluble
Log *S* (Ali)	-6.12
Solubility	3.09e-04 mg/ml ; 7.50e-07 mol/l
Class	Poorly soluble
Log *S* (SILICOS-IT)	-8.34
Solubility	1.89e-06 mg/ml ; 4.60e-09 mol/l
Class	Poorly soluble

Pharmacokinetics	
GI absorption	High
BBB permeant	No
P-gp substrate	No
CYP1A2 inhibitor	No
CYP2C19 inhibitor	Yes
CYP2C9 inhibitor	Yes
CYP2D6 inhibitor	No
CYP3A4 inhibitor	No
Log K_p (skin permeation)	-5.57 cm/s

Druglikeness	
Lipinski	Yes; 0 violation
Ghose	Yes
Veber	Yes
Egan	Yes
Muegge	Yes
Bioavailability Score	0.55

Medicinal Chemistry	
PAINS	0 alert
Brenk	0 alert
Leadlikeness	No; 2 violations: MW>350, XLOGP3>3.5
Synthetic accessibility	3.52

Figure S42. SwissADME results for molecule **21a**.

21a

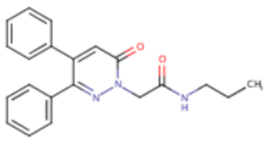

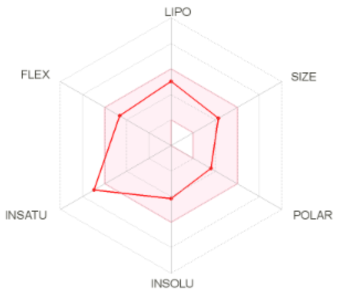

SMILES CCCNC(=O)Cn1nc(c2ccccc2)c(cc1=O)c1ccccc1

Physicochemical Properties	
Formula	C21H21N3O2
Molecular weight	347.41 g/mol
Num. heavy atoms	26
Num. arom. heavy atoms	18
Fraction Csp3	0.19
Num. rotatable bonds	7
Num. H-bond acceptors	3
Num. H-bond donors	1
Molar Refractivity	102.86
TPSA	63.99 Å²

Lipophilicity	
Log $P_{o/w}$ (iLOGP)	3.09
Log $P_{o/w}$ (XLOGP3)	3.32
Log $P_{o/w}$ (WLOGP)	3.10
Log $P_{o/w}$ (MLOGP)	2.78
Log $P_{o/w}$ (SILICOS-IT)	3.83
Consensus Log $P_{o/w}$	3.22

Water Solubility	
Log *S* (ESOL)	-4.14
Solubility	2.54e-02 mg/ml ; 7.31e-05 mol/l
Class	Moderately soluble
Log *S* (Ali)	-4.34
Solubility	1.59e-02 mg/ml ; 4.57e-05 mol/l
Class	Moderately soluble
Log *S* (SILICOS-IT)	-7.33
Solubility	1.62e-05 mg/ml ; 4.66e-08 mol/l
Class	Poorly soluble

Pharmacokinetics	
GI absorption	High
BBB permeant	Yes
P-gp substrate	No
CYP1A2 inhibitor	No
CYP2C19 inhibitor	Yes
CYP2C9 inhibitor	Yes
CYP2D6 inhibitor	No
CYP3A4 inhibitor	Yes
Log K_p (skin permeation)	-6.06 cm/s

Druglikeness	
Lipinski	Yes; 0 violation
Ghose	Yes
Veber	Yes
Egan	Yes
Muegge	Yes
Bioavailability Score	0.55

Medicinal Chemistry	
PAINS	0 alert
Brenk	0 alert
Leadlikeness	Yes
Synthetic accessibility	3.31

Figure S43. SwissADME results for molecule **21d**.

21d

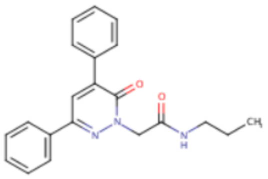

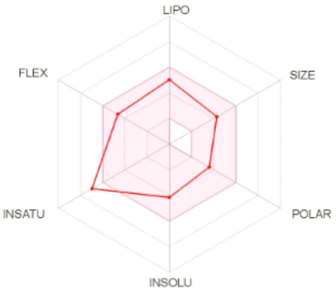

SMILES CCCNC(=O)Cn1nc(cc(c1=O)c1ccccc1)c1ccccc1

Physicochemical Properties	
Formula	C21H21N3O2
Molecular weight	347.41 g/mol
Num. heavy atoms	26
Num. arom. heavy atoms	18
Fraction Csp3	0.19
Num. rotatable bonds	7
Num. H-bond acceptors	3
Num. H-bond donors	1
Molar Refractivity	102.86
TPSA	63.99 Å²

Lipophilicity	
Log $P_{o/w}$ (iLOGP)	3.18
Log $P_{o/w}$ (XLOGP3)	3.32
Log $P_{o/w}$ (WLOGP)	3.10
Log $P_{o/w}$ (MLOGP)	2.78
Log $P_{o/w}$ (SILICOS-IT)	3.83
Consensus Log $P_{o/w}$	3.24

Water Solubility	
Log *S* (ESOL)	-4.14
Solubility	2.54e-02 mg/ml ; 7.31e-05 mol/l
Class	Moderately soluble
Log *S* (Ali)	-4.34
Solubility	1.59e-02 mg/ml ; 4.57e-05 mol/l
Class	Moderately soluble
Log *S* (SILICOS-IT)	-7.33
Solubility	1.62e-05 mg/ml ; 4.66e-08 mol/l
Class	Poorly soluble

Pharmacokinetics	
GI absorption	High
BBB permeant	Yes
P-gp substrate	No
CYP1A2 inhibitor	No
CYP2C19 inhibitor	Yes
CYP2C9 inhibitor	Yes
CYP2D6 inhibitor	No
CYP3A4 inhibitor	Yes
Log K_p (skin permeation)	-6.06 cm/s

Druglikeness	
Lipinski	Yes; 0 violation
Ghose	Yes
Veber	Yes
Egan	Yes
Muegge	Yes
Bioavailability Score	0.55

Medicinal Chemistry	
PAINS	0 alert
Brenk	0 alert
Leadlikeness	Yes
Synthetic accessibility	3.23

Table S19. pkCSM absorption results

mol	Water solubility	Caco2 permeability	Intestinal absorption (human)	Skin Permeability	P-glycoprotein substrate	P-glycoprotein I inhibitor	P-glycoprotein II inhibitor
**1a**	-5.127	1.075	100	-2.73	No	Yes	Yes
**2a**	-5.72	1.087	99.635	-2.712	No	Yes	Yes
**3a**	-2.944	1.441	95.475	-2.673	No	No	No
**5a**	-4.58	1.407	98.203	-2.701	No	No	Yes
**7a**	-3.761	0.893	95.774	-2.751	No	No	Yes
**8**	-5.603	1.01	87.741	-2.736	Yes	Yes	Yes
**9**	-4.621	1.183	95.913	-2.735	Yes	Yes	Yes
**10b**	-5.176	1.028	100	-2.726	No	Yes	Yes
**10g**	-3.335	1.355	99.99	-2.448	No	No	No
**11**	-4.673	1.341	98.693	-2.557	No	Yes	Yes
**13a**	-4.269	1.034	94.435	-2.737	Yes	Yes	Yes
**15a**	-4.175	1.199	97.888	-2.751	No	Yes	Yes
**15f**	-4.74	1.029	96.488	-2.735	Yes	Yes	Yes
**17a**	-4.442	1.052	91.796	-2.738	Yes	Yes	Yes
**20a**	-4.815	1.17	96.381	-2.735	Yes	Yes	Yes
**21a**	-4.844	0.909	97.177	-2.705	Yes	Yes	Yes
**21d**	-4.773	0.856	95.71	-2.708	Yes	Yes	Yes

Table S20. pkCSM distribution results

mol	VDss (human)	Fraction unbound (human)	BBB permeability	CNS permeability
**1a**	0.1	0.202	-0.451	-2.46
**2a**	0.242	0.167	-0.372	-2.155
**3a**	-0.45	0.302	-0.157	-2.902
**5a**	-0.139	0.121	0.628	-2.016
**7a**	-0.223	0.131	0.001	-2.191
**8**	-0.318	0.045	-0.452	-1.898
**9**	-0.122	0.181	-1.149	-2.285
**10b**	0.035	0.267	0.395	-2.03
**10g**	-0.094	0.207	0.388	-2.297
**11**	-0.045	0.134	0.084	-1.947
**13a**	-0.563	0.042	0.14	-2.362
**15a**	-0.445	0.022	-0.119	-2.256
**15f**	-1.036	0.17	0.09	-1.978
**17a**	-0.184	0.086	0.166	-2.02
**20a**	-0.799	0.098	-0.013	-2.122
**21a**	-0.24	0.201	0.263	-2.33
**21d**	-0.243	0.179	0.336	-2.291

Table S21. pkCSM metabolism results

mol	CYP2D6 substrate	CYP3A4 substrate	CYP1A2 inhibitior	CYP2C19 inhibitior	CYP2C9 inhibitior	CYP2D6 inhibitior	CYP3A4 inhibitior
**1a**	No	Yes	Yes	Yes	Yes	No	Yes
**2a**	No	Yes	Yes	Yes	Yes	No	Yes
**3a**	No	No	Yes	No	No	No	No
**5a**	No	Yes	Yes	Yes	Yes	No	No
**7a**	No	Yes	Yes	Yes	No	No	Yes
**8**	No	Yes	No	Yes	Yes	No	Yes
**9**	No	Yes	No	Yes	Yes	No	Yes
**10b**	No	Yes	Yes	Yes	Yes	No	Yes
**10g**	No	Yes	Yes	Yes	Yes	No	Yes
**11**	No	Yes	Yes	Yes	Yes	No	No
**13a**	No	Yes	Yes	Yes	No	No	Yes
**15a**	No	Yes	Yes	Yes	Yes	No	Yes
**15f**	No	Yes	Yes	Yes	Yes	No	Yes
**17a**	No	Yes	Yes	Yes	Yes	No	Yes
**20a**	No	Yes	Yes	Yes	Yes	No	Yes
**21a**	No	Yes	Yes	Yes	Yes	No	Yes
**21d**	No	Yes	Yes	Yes	Yes	No	Yes

Table S22. pkCSM excretion results

mol	Total Clearance	Renal OCT2 substrate
**1a**	0.353	No
**2a**	0.304	No
**3a**	0.527	No
**5a**	0.237	Yes
**7a**	0.189	No
**8**	-0.79	No
**9**	-0.422	No
**10b**	1.161	No
**10g**	1.087	Yes
**11**	0.564	No
**13a**	0.286	No
**15a**	0.331	No
**15f**	0.195	No
**17a**	0.223	No
**20a**	0.563	No
**21a**	0.558	No
**21d**	0.438	No

Table S23. pkCSM toxicity results

mol	AMES toxicity	Max. tolerated dose (human)	hERG I inhibitor	hERG II inhibitor	Oral Rat Acute Toxicity (LD50)	Oral Rat Chronic Toxicity (LOAEL)	Skin Sensitisation	Minnow toxicity
**1a**	No	0.632	No	Yes	2.455	1.119	No	-1.537
**2a**	No	0.629	No	Yes	2.411	1.005	No	-2.409
**3a**	No	1.017	No	No	2.301	1.996	No	1.424
**5a**	No	0.56	No	Yes	2.258	1.165	No	-0.459
**7a**	No	0.36	No	Yes	3.057	0.988	No	0.542
**8**	No	0.556	No	Yes	2.615	0.727	No	-1.355
**9**	No	0.525	No	Yes	3.089	0.434	No	0.162
**10b**	No	0.759	No	Yes	2.233	0.507	No	-0.965
**10g**	Yes	-0.136	No	No	2.064	1.299	No	1.439
**11**	No	0.383	No	No	2.058	1.574	No	-0.022
**13a**	No	0.502	No	Yes	2.91	1.116	No	0.861
**15a**	No	0.089	No	Yes	2.627	1.227	No	-1.131
**15f**	Yes	0.452	No	Yes	3.137	0.538	No	-2.663
**17a**	Yes	0.205	No	Yes	2.93	0.81	No	-1.849
**20a**	Yes	0.445	No	Yes	3.175	0.7	No	-2.595
**21a**	Yes	0.73	No	Yes	1.929	1.268	No	-0.053
**21d**	Yes	0.79	No	Yes	1.919	1.267	No	-0.315

4. References

For Refs [18] and [23-34] in SI, see reference list in main manuscript.
